# Reprogramming of the Aurantinin Polyketide Assembly Line to Synthesize Auritriacids by Excising an Atypical Enoyl‐CoA Hydratase Domain

**DOI:** 10.1002/advs.202401708

**Published:** 2024-07-12

**Authors:** Dacheng Wang, Huijin Mao, Zelian Zhao, Lilu Liu, Yihua Chen, Pengwei Li

**Affiliations:** ^1^ State Key Laboratory of Microbial Resources Institute of Microbiology Chinese Academy of Sciences Beijing 100101 China; ^2^ University of Chinese Academy of Sciences Beijing 100049 China; ^3^ School of Life Sciences Yunnan University Kunming 650500 China

**Keywords:** assembly line, aurantinin, enoyl‐CoA hydratase, polyketide synthase, reprogamming

## Abstract

Modular polyketide synthases (PKSs) are capable of synthesizing diverse natural products with fascinating bioactivities. Canonical enoyl‐CoA hydratases (ECHs) are components of the *β*‐branching cassette that modifies the polyketide chain by adding a *β*‐methyl branch. Herein, it is demonstrated that the deletion of an atypical ECH_Q_ domain (featuring a Q^280^ residue) of Art21, a didomain protein contains an ECH_Q_ domain and a thioesterase (TE) domain, reprograms the polyketide assembly line from synthesizing tetracyclic aurantinins (ARTs) to bicyclic auritriacids (ATAs) with much lower antibacterial activities. Genes encoding the ECH_Q_‐TE didomain proteins distribute in many PKS gene clusters from different bacteria. Significantly, the ART PKS machinery can be directed to make ARTs, ATAs, or both of them by employing appropriate ECH_Q_‐TE proteins, implying a great potential for using this reprogramming strategy in polyketide structure diversification.

## Graphical abstract

It is demonstrated that the deletion of Art21‐ECH domain can reprogram the ART polyketide assembly line to synthesize ATAs with much lower antibacterial activities. Genes encoding ECH_Q_‐TE proteins are observed in a number of polyketide gene clusters, indicating that such a reprogramming strategy is adopted by many different bacteria to expand the chemical diversity of polyketides.

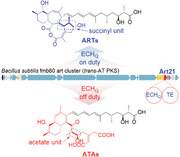

## 1 Introduction

A great number of natural products with impressive biological activities, e.g. erythromycin (antibiotic), lovastatin (cholesterol‐lowering), and avermectin (anti‐parasite), are synthesized by modular polyketide synthases (PKSs), which use ketosynthase (KS) domains to catalyze continuous Claisen condensation reactions to assemble the polyketide chain.^[^ ^1^ ^]^ The extending units are acyl carrier protein (ACP)‐tethered malonyl or *α*‐substituted malonyl building blocks loaded by acyltransferase (AT) domains.^[^ ^2^ ^]^ In addition to the three essential domains (KS, AT, and ACP) for polyketide chain extension, a module may also contain ketoreductase (KR), dehydratase (DH), enoyl reductase (ER), and methyltransferase (MT) domains that modify the newly synthesized *β*‐ketothioester intermediates. A thioesterase (TE) domain is usually found at the end of the assembly line to offload the mature polyketide chain.^[^ ^3^ ^]^ Except for the *cis*‐AT PKSs that have an AT domain in each module, there are also numerous *trans*‐AT PKSs, in which AT is a standalone protein that transfers CoA‐activated acyl units to the ACP domains of modular PKSs *in trans*.^[^ ^4^ ^]^

**Figure 1:**
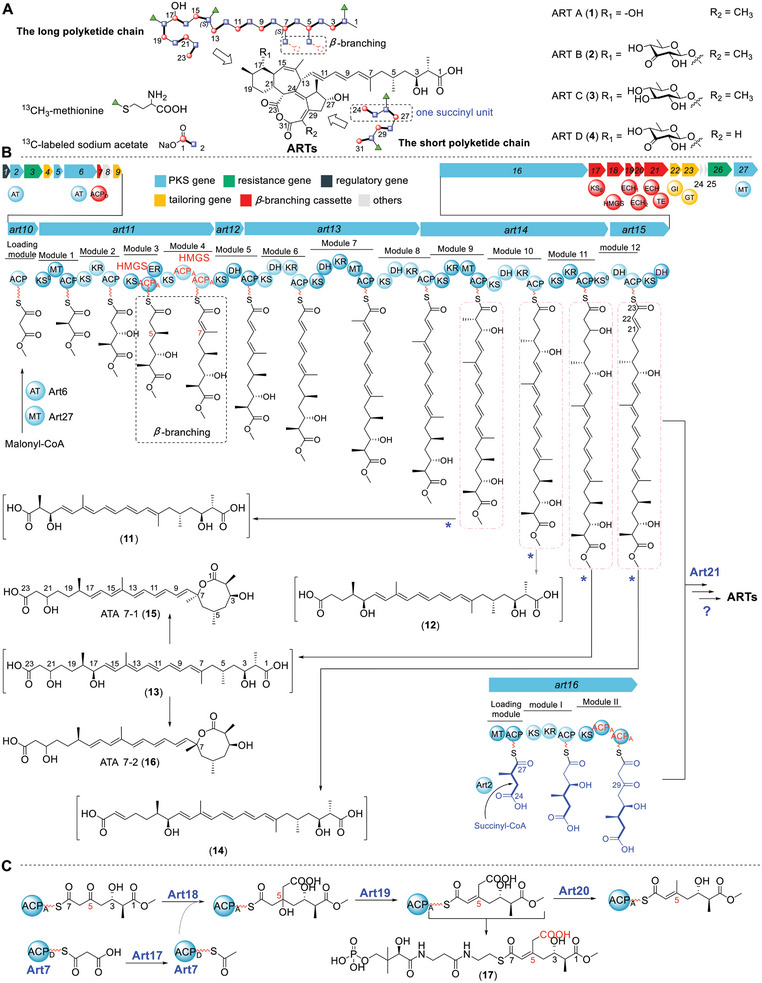
Biosynthesis of ARTs by the ART assembly line with the presence of Art21‐ECH. A) Structures and the isotope labeling pattern of ARTs. Red circles represent carbons labeled by [1‐^13^C] sodium acetate, blue squares represent carbons labeled by [2‐^13^C] sodium acetate, and green triangles represent carbons labeled by [^13^CH_3_] methionine; B) The *art* biosynthetic gene cluster and the two‐chain fusion biosynthesis model of ARTs. The blue asterisks indicate two hydrolysis reactions that release the polyketide chain from the thioester bond and hydrolyze the terminal methylester bond by Art9; C) Representative *β*‐branching process occurred on C‐5. ACP_D_, donor ACP; ACP_A,_ accepter ACP. **17** represents the phosphopantetheine attached substrate of Art20.

Aurantinins (ARTs) from *Bacillus* are a group of antibiotics composed of a unique 6/7/8/5‐fused tetracyclic ring system and a fatty acid side chain with a triene moiety.^[^ ^5^ ^]^ The major component, ART B (**2**), is derived from ART A (**1**) by attaching a 3‐keto‐6‐deoxy‐*β*‐d‐glucose to its 17‐OH. ART C (**3**) and ART D (**4**) are two congeners of ART B isolated as minor components.^[^ ^6^ ^]^ Interestingly, ^13^C isotope labeling studies suggested the incorporation of a four‐carbon succinyl unit into the ART skeleton (**Figure** **1**A). Based on this, a two‐chain fusion model including a long and a short polyketide chains was proposed for the formation of ART skeleton (Figure 1B).^[^ ^7^ ^]^ In our previous study, we demonstrated that, in *Bacillus Subtilis* fmb60, ARTs are synthesized by *trans*‐AT PKSs, utilizing an unusual online methyl esterification mechanism to initiate polyketide biosynthesis by an *O*‐methyltransferase, Art27 (Figure 1B). At the late biosynthetic stage, the methyl ester is hydrolyzed to release the carboxyl terminus of ARTs by an esterase, Art9.^[^ ^8^ ^]^

For the *art* gene cluster, we noted that it has a redundant copy of enoyl‐CoA hydratase (ECH) gene that usually participates in the *β*‐branching process (Figure 1B). Here, we show that the atypical ECH domain of Art21, a didomain ECH‐TE protein, is not involved in *β*‐branching, but is indispensable for the succinyl unit incorporation of ARTs. When Art21‐ECH was deleted, the ART biosynthetic machinery was switched from synthesizing ARTs using both malonyl and succinyl unit to assembling auritriacids (ATAs) using only malonyl unit. Furthermore, the cross complementation studies revealed that Art21‐like proteins from different bacteria of two phyla were able to influence the ART polyketide assembly line as Art21, implying a wide distribution of this polyketide assembly line reprogramming phenomenon.

## 2 Results

### 2.1 Art21‐ECH is an Atypcial ECH

According to the isotope labeling studies, the C‐5 and C‐7 methyl groups of ART were proposed to be incorporated via *β*‐branching (Figure 1A).^[^ ^8^ ^]^ Consistent with this, the ACPs of ART PKS module 3 and 4 are all *β*‐branching acceptor ACPs that possess a featuring GXDSX_5_W motif (Figure S1, Supporting Information).^[^ ^9^ ^]^ Meanwhile, all necessary components of a canonical *β*‐branching cassette can be found in the *art* gene cluster, including genes that encoding donor ACP (*art7*),^[^ ^10^ ^]^ specific KS^0^ (*art17*), 3‐Hydroxy‐3‐methylglutaryl synthase (*art18*), and two ECHs (ECH_1_ for dehydration and ECH_2_ for decarboxylation) (Figure 1B).^[^ ^11^ ^]^ To our surprise, there are three ECH genes (*art19*, *art20*, and *art21*) in the *art* gene cluster, which aroused our interests to study their functions.

Multiple sequence alignments suggested Art19 and Art20 as typical ECH_1_^[^ ^12^ ^]^ and ECH_2_,^[^ ^13^ ^]^ respectively (Figures S2 and S3, Supporting Information). They display high similarities with the identified ECH pairs, e.g. Art19 and Art20 share 53.1% and 69.2% identities with the ECH_1_ (PksH) and ECH_2_ (PksI) involved in bacillaene biosynthesis, respectively.^[^ ^14^ ^]^ When they were deleted individually (Figure S4 and Tables S1 and S2, Supporting Information), both of the *B. Subtilis* Δ*art19* and Δ*art20* mutants were incapable of producing ARTs or any detectable intermediates (Figure 3A). In addition, in vitro enzymatic assays of Art19 and Art20 using 3‐hydroxy‐3‐methylglutaryl coenzyme A (HMG‐CoA) as a substrate verified that Art19 and Art20 had the ECH_1_ dehydration and the ECH_2_ decarboxylation activities, respectively (Figure 1C; Figure S5, Supporting Information).^[^ ^14^ ^]^

**Figure 2:**
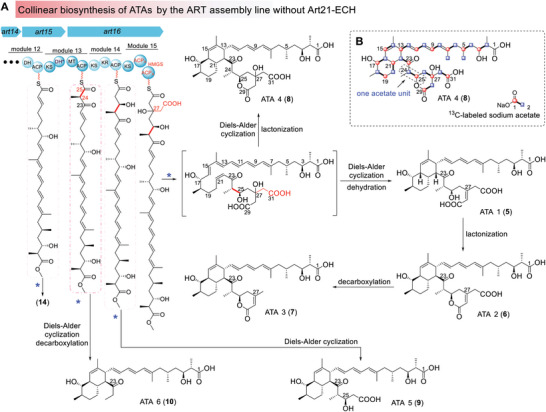
Biosynthesis of ATAs by the ART assembly line without Art21 (ECH_Q_‐TE). A) The proposed collinear biosynthesis pathway of ATAs. The blue asterisk indicates two hydrolysis reactions on the thioester bond and the methylester bond; B) Isotope labeling pattern of ATA 4 (**8**), red circles represent carbons labeled by [1‐^13^C] sodium acetate and blue squares represent carbons labeled by [2‐^13^C] sodium acetate.

**Figure 3:**
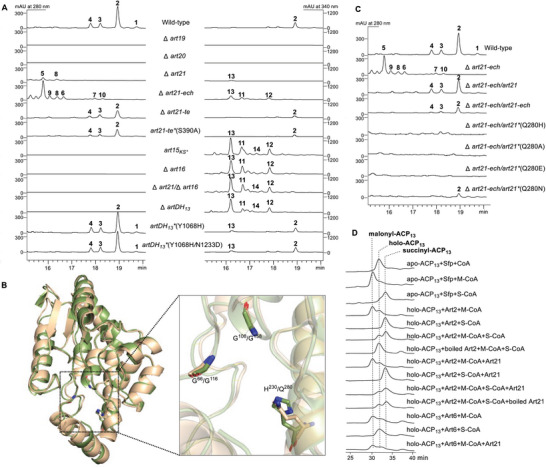
HPLC profiles of different *B. Subtilis* mutant and complementation strains and in vitro enzymatic assays of Art21. A) HPLC analysis of *B. Subtilis* wild‐type and the mutant strains; B) Structural alignment of the structures of Art21‐ECH (wheat, AlphaFold predicted) and PksI (green, PDB No:4q1h) and visualization of the active site of Art21‐ECH (G^116^, G^158^, Q^280^) and PksI (G^66^, G^108^, H^230^); C) HPLC analysis of different complementation strains of *B. Subtilis* Δ*art21‐ech*; D) HPLC analysis of the Art21 assays toward Art2 or Art6‐catalyzed acyltransfer reactions. The succinyl‐ACP_13_ and malonyl‐ACP_13_ standards were prepared by incubating apo‐ACP_13_ with Sfp and succinyl‐CoA (S‐CoA) and malonyl‐CoA (M‐CoA), respecitvely.

Art21 is a didomain protein containing an ECH domain and a TE domain. The *N*‐terminal ECH domain (Art21‐ECH, residues 1 to 299) shares 56.1% and 58.5% identities with Art20 and PksI,^[^ ^15^ ^]^ respectively. Of note, the key catalytic histidine residue that is essential for the decarboxylation activity of ECH_2_ is replaced by a glutamine (Q^280^) in Art21‐ECH, indicating that this atypcial ECH domain acts an unusual role (Figure S3, Supporting Information). The *C*‐terminal TE domain (Art21‐TE, residues 300 to 603) shares 44.4% identity with KtzF, a type II TE involved in kutznerides biosynthesis.^[^ ^16^ ^]^ When the whole *art21* gene was in‐frame deleted (Figure S4 and Table S2, Supporting Information), the mutant strain, *B. Subtilis* Δ*art21*, could not synthesize ARTs any more but accumulated compounds **5**–**10** possessing the characteristic UV absorption of a triene moiety (**Figures** **2**A and **3**A; Figure S6, Supporting Information), indicating that they are intermediates or shunt products of ART biosynthesis.

To dissect the two different domains of Art21, we constructed three mutant strains *B. Subtilis* Δ*art21‐ech*, Δ*art21‐te*, and *art21‐te* ^*^(S390A) (Figure S7, Supporting Information). In *B. Subtilis* Δ*art21‐ech*, the Art21‐ECH domain was inactivated by deleting residues E^72^–L^265^; in *B. Subtilis* Δ*art21‐te*, the Art21‐TE domain was deleted by excising residues S^300^–D^602^; in *B. Subtilis art21‐te* ^*^(S390A), the catalytic Ser residue essential to type II TE (S^390^ of Art21‐TE domain) was point mutated to Ala. When fermented in the ART production conditions, *B. Subtilis* Δ*art21‐ech* lost the ability to synthesize ARTs and accumulated considerable amounts of compounds **5**–**10**. Both *B. Subtilis* Δ*art21‐te* and *art21‐te* ^*^(S390A) produced ARTs at much lower levels but did not accumulate **5**–**10** (Figure 3A). Taken collectively, we proposed that compounds **5**–**10** will be synthesized when Art21‐ECH is absent, and Art21‐TE may act as a type II TE that ensures the high catalytic efficiency of PKS assembly line by removing the aberrant extension units from ACPs.^[^ ^17^ ^]^

### 2.2 Collinear Biosynthesis of ATAs by ART PKSs in the Absence of Art21‐ECH

Isolation of compounds **5–10** was carried out using *B. Subtilis* Δ*art21‐ech* as a producer. Spectroscopic analyses of the main product (**5**) (Figure S8 and Table S3, Supporting Information) revealed that it has the same fatty acid side chain as ARTs but lacks the complicated tetracyclic ring system (**Figure** **2**A). Instead, **5** has a 6/6‐bicyclic ring moiety and a second side chain that forks at C‐27 and ends with two carboxylic acids. It was therefore named as auritriacid‐1 (ATA1) based on the fact that it has three carboxylic acid groups, which is rare for natural products. During this process, we noticed that **5** is not stable. It underwent a spontaneous lactonization to form **6** (ATA2), and then a slow decarboxylation to generate **7** (ATA3) (Figures S9–S11 and Tables S4 and S5, Supporting Information). Unlike **5**, **8** (ATA4) is stable at room temperature with a C‐27 hydroxyl group and a lactonized terminus (Figure S12 and Table S6, Supporting Information). *B. Subtilis* Δ*art21‐ech* also produced two congeners of **5** with shorter chains. Compound **9** (ATA5) ends at C‐27 and lacks the two branched carboxymethyl ends of **5** (Figure S13 and Table S7, Supporting Information); the side chain of compound **10** (ATA6) (Figure S14 and Table S8, Supporting Information) is even shorter and ends with a methyl group instead of a carboxylic terminus (Figure 2A).

Based on the domain organization of ART assembly line and the structures of ATAs, we proposed that ATAs are synthesized by ART PKSs in a one‐polyketide‐chain manner that conforms to the collinearity rule well (Figure 2A). After the first 12 modules establish the ACP_12_‐tethered intermediate, module 13 extends the polyketide chain by two carbons using malonyl‐CoA as a building block and then adds a methyl group at C‐24 by the MT_13_ domain. Compound **10** will be generated if the resultant polyketide chain is hydrolyzed from ACP_13_ and undergoes a spontaneous decarboxylation at C‐25, and then the terminal methyl ester will be hydrolyzed by Art9 to release the methyl group from C‐1.^[^ ^8^ ^]^ The ACP_13_‐tethered polyketide may undergo one more round of elongation and ketoreduction by module 14, and **9** will be generated if it is hydrolyzed from ACP_14_ and modified by Art9. At last, module 15 will extend the ACP_14_ ‐tethered polyketide chain by two carbons. Subsequently, the *β*‐branching enzymes will add an carboxymethyl branch at C‐27. Consistent with this, the two ACP domains of module 15 are both *β*‐branching acceptor ACPs with a GXDSX_5_W motif (Figure S1, Supporting Information). **8** will be obtained if the polyketide intermediate is hydrolyzed from the assembly line, modified by Art9, and lactonized. At last, the main ATA product (**5**) will be generated if the polyketide intermediate is released from the assembly line after it is dehydrated by ECH_1_ (Art19) and modified by Art9. Compounds **6** and **7** are derived from **5** by spontaneous lactonization and decarboxylation. The isotope labeling studies on ATAs (**5**, **6**, and **8**) (Figure 2B; Figures S8C, S10D, and S12D, Supporting Information) suggested that their skeletons are installed in a consecutive two‐carbon extension manner and involve no succinyl unit, which also support the one‐polyketide‐chain model of ATA biosynthesis.

### 2.3 ARTs and ATAs are Synthesized by the Same PKS Machinery

When comparing the one‐polyketide‐chain model of ATAs with the two‐chain fusion model of ARTs, in which KS_13_ is assumed to connect the long chain with the short chain synthesized by Art16 using one succinyl and two malonyl units,^[^ ^8^ ^]^ we were tentative in proposing that ARTs and ATAs are both installed by the ART PKSs. To avoid that some genes beyond the *art* gene cluster participate in ART biosynthesis, we analyzed the genome of *B. Subtilis* fmb60 carefully but failed to find any other PKS genes. Thus, we inactivated KS_13_ by point mutagenesis at its active site residue (C595A) to afford B. Subtilis art15_KS*_, which could not produce either ARTs or ATAs but accumulated a series of products with the characteristic UV absorption of pentaene (**11–14**) (**Figure** **3**A). Compounds **11**–**14** are quite instable. When we tried to isolate the main product **13**, we observed a quick conversion of **13**–**15** and **16** (Figure S15, Supporting Information), whose molecular weights are both 18 Da less than **13** (Figures S16–S18, Supporting Information) and will interconvert to each other during the isolation process. Fortunately, by controlling the purification conditions strictly, we got enough amount of **15** and **16** for structure elucidation and assigned them as two isomers with an 8‐membered lactone ring (Figure 1B; Figures S16 and S17 and Tables S9 and S10, Supporting Information). We proposed that when Art15‐KS was inactivated, the ACP_11_ tethered intermediate was offloaded from the assembly line and converted to **13** by excising its terminal methyl group by Art9. Compound **13** underwent a^[^ ^1^, ^11^ ^]^ sigmatropic like shift and a spontaneous lactonization to form **15** and **16**. Based on the MS data (Figure S18, Supporting Information), compounds **11**, **12**, and **14** were proposed to be analogues of **13** that are generated from the ACP_9_‐, ACP_10_‐, and ACP_12_‐tethered intermediates via similar hydrolysis reactions (Figure 1B). These results implied that KS_13_ is indispensable for the biosynthetic processes that convert the ACP_12_‐tethered polyketide intermediate to generate ARTs and ATAs.

To investigate that whether Art16 participates in those biosynthetic processes, an in‐frame deletion mutant of the 8.0‐kb *art16* gene was constructed (Figure S4, Supporting Information). *B. Subtilis* Δ*art16* was unable to synthesize any ARTs or ATAs and accumulated **11–14** as well as B. Subtilis art15_KS*_. We also deleted gene *art16* in *B. Subtilis* Δ*art21‐ech* and the resultant double mutant strain *B. Subtilis* Δ*art21‐ech/*Δ*art16* displayed the same metabolic profile as *B. Subtilis* Δ*art16* (Figure 3A). Collectively, our results are in agreement with the proposal that both ARTs and ATAs are synthesized by the ART PKS machinery, and the absence of Art21‐ECH switches the PKS machinery from synthesizing ARTs with an unusual succinyl unit to making ATAs following the collinearity rule.

### 2.4 Residue Q^280^ is Important to the Reprogramming Activity of Art21‐ECH

To gain deep insights into Art21, we tried to complement the *B. Subtilis* Δ*art21‐ech* mutant by expressing *art21* or *art21‐ech in trans*. HPLC analysis showed that the introduction of either *art21* or *art21‐ech* (residues 1–299) into Δ*art21‐ech* could shut off the production of ATAs and restore the synthesis of ARTs, verifying that the Art21‐ECH domain is essential to ART production (Figure 3C).

Further analysis of the Art21‐ECH domain (residues 1–299) with Alphafold2 revealed that its overall structure aligned well with the structures of well‐characterized ECH_2_ proteins, e.g. PksI (PDB No:4q1h) (Figure 3B).^[^ ^15^ ^]^ PksI stabilizes the polyketide substrate by forming an oxyanion hole using the backbone amides of two glycine residues (G^66^ and G^108^) and initiates the decarboxylation reaction with H^230^ (Figure S3, Supporting Information). However, docking analysis of Art21‐ECH with **17** (Figure 1C; Figure S19, Supporting Information), the *β*‐branching intermediate attached to ACP_3_, revealed that **17** could not bind to Art21‐ECH as to typical ECH_2_ (e.g., Art20), and the histidine residue, as aforementioned, is replaced by a glutamine (Q^280^). To study the influence of Q^280^ on the reprogramming acitvity, we first mutated it back to histidine and tried to complement *B. Subtilis* Δ*art21‐ech* with Art21^*^(Q280H). To our surprise, neither ARTs nor ATAs was produced in *B. Subtilis* Δ*art21‐ech*/*art21* ^*^(Q280H) (Figure 3C; Figure S20, Supporting Information), suggesting that Art21^*^(Q280H) could derail ATA synthesis as Art21 but failed in reprogramming the polyketide assembly line to make ARTs. Such a phenomenon was also observed for *art21* ^*^(Q280A), further supporting that Art21 can block ATA production and the Q^280^ residue is necessary for enabling ART biosynthesis. Subsequently, Art21 Q^280^ was mutated to glutamic acid (Q280E) and asparagine (Q280N) and tested for *B. Subtilis* Δ*art21‐ech* complementation. Art21^*^(Q280E) only shut off the production of ATAs, while Art21^*^(Q280N) could go further and restore ART production to a certain level, indicating that the terminal amide group of residue 280 is important to the reprogramming activity (Figure 3C). We used HMG‐CoA as a substrate to set the *β*‐branching enzymatic assays of Art19 (ECH_1_) and Art21 (to replace Art20, the typical ECH_2_), which showed that Art21‐ECH cannot catalyze the decarboxylation as Art20. We also tested Art21^*^(Q280H) in the same enzymatic assay and failed to observe the ECH_2_ decarboxylation activity (Figure S5, Supporting Information). Overall, we proposed that Art21‐ECH domain can block ATA synthesis at module 13 and direct the assembly line to ART production, and the Q^280^ residue of Art21‐ECH is important to its reprogramming activity.

One plausible way of Art21 modulating polyketide biosynthesis is to act as a gatekeeper that prohibits malonyl unit loading to ACP_13_ and force Art15‐16 to install a chain with a succinyl unit. There are two AT genes, *art2* and *art6*, in the *art* gene cluster. Previous studies suggested Art2 as a succinyl transferase and Art6 as a malonyl transferase.^[^ ^18^ ^]^ The enzymatic studies revealed that Art2 could load succinyl onto ACP_13_ efficiently and also showed considerable activity toward malonyl‐CoA, while Art6 could only load malonyl onto ACP_13_. When Art21 was added to the enzymatic assays, no obvious change on the AT activities of Art2 or Art6 was observed from HPLC and intact protein MS analyses (Figure 3D; Figures S21 and S22 and Table S11, Supporting Information), implying that Art21 does not control the biosynthetic program by directing acyl transfer of ACP_13_.

Another possibility is that Art21 interacts with module 13 to block the Claisen condensation reaction that uses malonyl as an extending unit and leads the polyketide assembly line to synthesize ARTs. We compared the four domains of module 13 with domains from the other Art modules carefully (Tables S12–S15, Supporting Information) and noted that the similarity between DH_13_ with the other Art DHs are fairly low (< 28% identities). Furthermore, in DH_13_, the conserved histidine‐aspartate dyad of typical DH active site is substituted by Tyr^1068^ and Asn^1233^, respectively, suggesting that it is a dysfunctional DH (Figure S23, Supporting Information). To probe the function of DH_13_, we mutated Tyr^1068^ to histidine to construct *B. Subtilis artDH_13_* ^*^(Y1068H), in which the production of ARTs was slightly reduced and a small amount of **13** was accumulated (Figure 3A). When we further replaced Asn^1233^ with aspartate, the double mutated strain *B. Subtilis artDH_13_* ^*^(Y1068H/N1233D) had a similar metabolic profile as that of *B. Subtilis artDH_13_* ^*^(Y1068H). However, when the DH_13_ domain was removed by excising residues 1067 to 1360 of Art15, neither ARTs nor ATAs was produced, while **11–14** were massively accumulated (Figure 3A). It seems that, although DH_13_ is not an active dehydratase, this domain is indispensable for the biosynthesis of ARTs. It is reminiscent of the branching (B) domain that participates in the installation of *δ*‐lactone and glutarimide moieties in polyketides.^[^ ^19^ ^]^ The B domain features a double hot dog fold for typical DH domains, but lacks the histidine‐aspartate dyad. It was proposed to act a structural role to keep the KS‐B‐ACP module functional.^[^ ^19^ ^]^

### 2.5 Characterization of the Homologous Proteins of Art21

**Figure 4:**
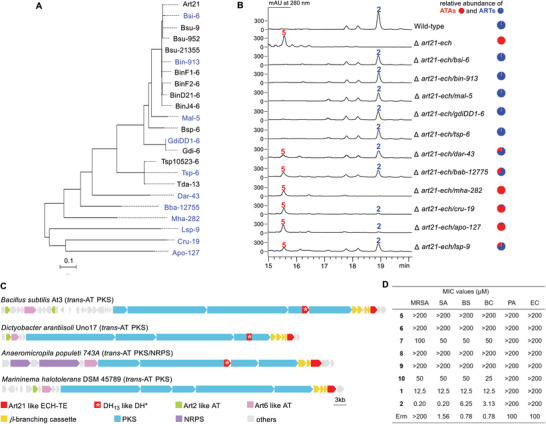
Distribution and in vivo characterization of the ECH_Q_‐TE proteins. A) Phylogenetic analysis of the ECH_Q_‐TE proteins, blue ones were picked for the complementation of *B. Subtilis* Δ*art21‐ech*. B) HPLC analysis of the complementation activties of different ECH_Q_‐TE proteins toward *B. Subtilis* Δ*art21‐ech*; C) Representative biosynthetic gene clusters containing a gene encoding the ECH_Q_‐TE protein; D) Antibacterial activities of ATAs and ARTs. Methicillin‐resistant *Staphylococcus aureus* strain 113 (MRSA), *Staphylococcus aureus* ATCC 6538 (SA), *Bacillus subtilis* BS168 (BS), *Bacillus cereus* CGMCC 1. 0230 (BC), *Pseudomonas aeruginosa* (PA), *Escherichia coli* CGMCC 1. 12873 (EC), erythromycin (Erm).

We used Art21‐ECH (residues 1–299) as a probe to search the NCBI database with BlastP (> 40% identity and > 60% coverage) and obtained 48 single domain ECHs and 23 ECH‐TE didomain proteins. All of the single domain ECHs were putative typical ECH_2_ with the conserved catalytic His residue (Figure S24, Supporting Information); in contrast, all of the 23 ECH‐TEs possess the featuring glutamine residue as Q^280^ in Art21‐ECH (Figure S25, Supporting Information), indicating that all of the atypical ECHs as Art21‐ECH exist as ECH‐TE didomain proteins. (**Figure** **4**A; Table S16, Supporting Information). Therefore, we named these homologous proteins as ECH_Q_‐TEs.

Except one protein (Dar43) from Chloroflexi, all of the other 22 ECH_Q_‐TE proteins (including Art21) are from Firmicutes. A phylogenetic analysis of the ECH_Q_‐TE proteins revealed that the orthologues from bacteria with close evolutionary relationship are unusually clustered in a same clade (Figure 4A). We chose 11 genes from varied bacteria genus, optimized their codons for *B. Subtilis*, and synthesized them for *B. Subtilis* Δ*art21‐ech* complementation. HPLC detection revealed that Bsi6, Bin913, Mal5, GdiDD1‐6, and Tsp6 (> 65% identities with Art21) could abolish ATA synthesis and restored the production of ARTs (Figure 4B). For the other six Art21‐like proteins (40% to 65% identities with Art21), five of them (Dar43, Bha12755, Lsp9, Cru19, and Apo127) could restore ART production to different levels, but none of them block ATA biosynthesis. This interesting observation suggested that blocking ATA production is not a prerequisite for starting ART biosynthesis and the ART assembly line is able to synthesize the two different polyketide skeletons simultaneously. In the case of Mha282, production of ATAs was decreased, but no ART was generated (Figure 4B). In summary, our results showed that most of the didomain ECH_Q_‐TE proteins can reprogram the ART polyketide assembly line, which aroused our interests in inspecting their gene clusters carefully.

All of the 23 ECH_Q_‐TE encoding genes locate just behind the *β*‐branching gene cassette that follows a PKS with at least two *β*‐branching acceptor ACP domains at its C‐terminus (Figure 4C; Figure S26, Supporting Information). Unfortunately, due to the poor sequencing qualities, only 6 of the 23 gene clusters, including the *art* gene cluster, are likely to be complete; the other 17 clusters are truncated at one or both ends (Figure S26, Supporting Information). Even though, DH^*^ domains that like Art‐DH_13_ with mutations on the conserved histidine‐aspartate dyad were discovered in 8 of the 23 clusters (Figures S26A and S27, Supporting Information). In addition, homologues of *art2* that encodes ATs prefer non‐malonyl unit could be observed in 7 gene clusters (Figure S28, Supporting Information). Considering that 10 of the 11 tested ECH_Q_‐TE proteins could enable ART biosynthesis in the cross complementation of *B. Subtilis* Δ*art21‐ech*, we assumed that the ECH_Q_‐TE proteins act similar roles as *art21* in their own gene clusters. Of note, 2 of the 23 clusters also contain non‐ribosomal peptide synthetase genes (Figure S26A, Supporting Information), further expanding the chemical space that may be explored with this set of polyketide assembly line reprogramming tools.

### 2.6 Antibacterial Activities of ATAs and ARTs

In the end, we tested the antibacterial activities of ATAs (**5**–**10**) together with ART A (**1**) and B (**2**) (Figure 4D). Compounds **5**, **6**, **8**, and **9** with carboxylic acid ends at both side chains barely showed any inhibition activities against all of the six tested bacteria. While, ATAs (**7** and **10**) that only have one free carboxyl terminus exhibited weak inhibition activities against the Gram‐positive bacteria. ARTs (**1** and **2**) had the same antibacterial spectra as **7** and **10** and displayed much stronger inhibition activities. Those results may lead an assumption of why bacteria evolve genes encoding the ECH_Q_‐TE proteins in PKS gene clusters. In the case of ART PKSs, the presence of Art21‐ECH prevents the assembly line from producing ATAs and directs it to synthesize a better antibiotic ART A, which can be further glycosylated to generate ART B, a powerful weapon that can help the producer to cope with the other bacteria.

## 3 Conclusion

In conclusion, we demonstrated that the deletion of Art21‐ECH_Q_ is able to reprogram the ART polyketide assembly line from synthesizing ARTs to producing ATAs with much lower antibacterial activities. The specific Q^280^ residue of Art21‐ECH_Q_ are important to the programming process, while the esterase activity of Art21‐TE domain is not necessary. Moreover, we found ECH_Q_ exist as a domain of the ECH_Q_‐TE didomain proteins in nature, and occur in a number of PKS and PKS/NRPS gene clusters. Many of them also exhibited reprogramming activity toward the ART polyketide assembly line, implying a wide distribution of this reprogramming strategy in polyketide biosynthesis. Significantly, the ART PKS machinery can be controlled to synthesize ARTs with a tetracyclic ring system, ATAs with a bicyclic ring system, or both of them by employing appropriate ECH_Q_‐TE proteins, demonstrating a great potential of applying this reprogramming strategy in polyketide structure diversification. Our findings set a stage for further studies regarding the detailed reprogramming mechanism of Art21‐ECH_Q_, which may enable us to utilize this type of atypical ECH_Q_ proteins as powerful synthetic biology tools to expand the chemical space of polyketides.

## 4 Experimental Section

### Bacterial Strains and Plasmids

All bacteria strains and plasmids used in this study are listed in Table S1 (Supporting Information).

### DNA Manipulation and Sequence Analysis

General DNA manipulations were performed as described.^[^ ^20^ ^]^ The primers used in this study (Table S2, Supporting Information) were synthesized by Generay (Shanghai, China). The PCR enzymes PrimeSTAR DNA polymerase (Takara, Japan) and Taq DNA polymerase (TransGene, Beijing, China) were used according to the manufacturers’ instructions. Common DNA sequencing was performed by TianYiHuiYuan Co. (Beijing, China). Electroporation of *B. subtilis* was performed as described.^[^ ^21^ ^]^ Gene function annotations were performed with BLAST (http://www.ncbi.nlm.nih.gov/blast). Multiple alignments were carried out using SnapGene 6.0.2 (https://www.statistical‐analysis.top/SnapGene/Details.html). Searching of the biosynthetic gene clusters containing *art21* homologues were performed with ClusterBlast of antiSMASH (https://antismash.secondarymetabolites.org/). Phylogenetic analysis was carried out with MEGA 6.06 (https://www.megasoftware.net/).

### Construction of B. subtilis Δart19, Δart20, Δart21, Δart21‐ech, Δart21‐te, ΔartDH_13_, Δart16, and Δart21‐ech/Δart16

The mutants were constructed by in‐frame deletion of each of the targeted genes (Figures S4 and S7, Supporting Information). For the construction of *B. subtilis* Δ*art19*, two 0.9‐kb DNA fragments flanking the *art19* gene were amplified with primer pairs 19‐L‐F/19‐L‐R and 19‐R‐F/19‐R‐R, respectively. The two fragments were inserted into the *Hin*dIII site of pRN5101 using one‐step cloning strategy to generate plasmid pRN5101::*art19*‐UD. After introduction of pRN5101::*art19*‐UD into *B. subtilis* fmb60, the erythromycin resistant transformants were selected as the single‐crossover mutants, and then cultured in LB without antibiotics at 37 °C overnight to induce the second crossover. After that, the erythromycin sensitive colonies were screened out by replicating colonies on plates with or without erythromycin. The sensitive colonies were verified as *B. subtilis* Δ*art19* mutant strains by PCR with primer pair 19‐L‐F/19‐R‐R and validated by DNA sequencing.

The other seven *B. subtilis* fmb60 mutants were constructed in similar ways as that of *B. subtilis* Δ*art19*, except the primers used for cloning the two flanking DNA fragments of the target genes or domains as well as primers for verification of the mutant constructions were different. Two 0.9‐kb fragments upstream and downstream of gene *art20* were amplified using primer pairs 20‐L‐F/20‐L‐R and 20‐R‐F/20‐R‐R, respectively. Two 0.9‐kb fragments flanking gene *art21* were amplified using primer pairs 21‐L‐F/21‐L‐R and 21‐R‐F/21‐R‐R, respectively. The 0.9‐kb upstream and 0.8‐kb downstream fragments flanking *art21‐ech* were amplified using primer pairs ECH‐L‐F/ECH‐L‐R and ECH‐R‐F/ECH‐R‐R, respectively. The 0.9‐kb upstream and 1.0‐kb downstream fragments flanking *art21‐te* were amplified using primer pairs TE‐L‐F/TE‐L‐R and TE‐R‐F/TE‐R‐R, respectively. Two 1.0‐kb fragments flanking *artDH_13_* were amplified using primer pairs DH‐L‐F/DH‐L‐R and DH‐R‐F/DH‐R‐R, respectively. Two 1.0‐kb fragments flanking *art16* were amplified using primer pairs 16‐L‐F/16‐L‐R and 16‐R‐F/16‐R‐R, respectively. For construction of the *B. subtilis* Δ*art21‐ech/*Δ*art16* double mutant, the *art21‐ech* of the Δ*art16* mutant strain was in‐frame deleted in a similar way. Successful constructions of *B. subtilis* Δ*art20*, Δ*art21*, and Δ*art21‐ech* (fragment from E^72^ to L2^65^ was in‐frame deleted), Δ*art21‐te* (fragment from S^300^ to D^602^ was in‐frame deleted), Δ*artDH_13_* (fragment from E^1067^ to L^1360^ was in‐frame deleted), Δ*art16* and Δ*art21‐ech/*Δ*art16* were verified by PCR with primer pairs 20‐L‐F/20‐R‐R, 21‐L‐F/21‐R‐R, ECH‐L‐F/ECH‐R‐R, TE‐L‐F/TE‐R‐R, DH‐L‐F/DH‐R‐R, 16‐L‐F/16‐R‐R, and ECH‐L‐F/ECH‐R‐R respectively. Correct in‐frame deletion of each of the targeted genes was validated by DNA sequencing.

### Site‐Directed Mutagenesis of Art21‐TE Domain and the KS and DH Domains of Art15

The conserved catalytic Ser^390^ residue of the TE domain of Art21 was mutated to Ala by site‐directed mutagenesis. The 1.0‐kb fragment upstream and 1.0‐kb fragment downstream the Ser^390^ codon of *art21* (with 43‐bp overlapping at the Ser^390^ codon) were amplified with primer pairs TEm‐L‐F/TEm‐L‐R and TEm‐R‐F/TEm‐R‐R, respectively. In primers TEm‐L‐R and TEm‐R‐F, the Ser^390^ codon (TCG) was replaced by an Ala codon (GCC). The two fragments were inserted into the *Hin*dIII site of pRN5101 using one‐step cloning strategy to generate plasmid pRN5101::*art21‐te*‐PM, which was introduced into *B. subtilis* fmb60 via electroporation. The erythromycin resistant transformants were selected as the single‐crossover mutants, which were cultured in LB without antibiotics at 37 °C overnight to induce the second crossover. The erythromycin sensitive colonies were screened out by replicating colonies on plates with or without erythromycin and were verified as the desired *B. subtilis art21‐te* ^*^(S390A) mutants by sequencing the fragments amplified by PCR with primer pair TEm‐L‐F/TEm‐R‐R.

For Art15, to mutate the conserved Cys^595^ residue of KS domain to Ala and the Tyr^1068^ residue of DH domain to His, they were constructed in a similar fashion as that of *art21‐te* ^*^(S390A) mutant. Briefly, two 0.9‐kb fragments flanking the Cys^595^ codon of *art15* (with 46‐bp overlapping at the Cys^595^ codon (TGT), which was changed to Ala codon (GGC)) were amplified with primer pairs KSm‐L‐F/KSm‐L‐R and KSm‐R‐F/KSm‐R‐R, respectively. The 1.0‐kb upstream and 0.9‐kb downstream fragments of the Tyr^1068^ codon of *art15* (with 42‐bp overlapping at the Tyr^1068^ codon (TAT), which was changed to His codon (CAC)) were amplified with primer pairs Ym‐L‐F/Ym‐L‐R and Ym‐R‐F/Ym‐R‐R, respectively. Mutant *B. subtilis artDH_13_* ^*^(Y1068H) was used to construct the double mutated strain *B. Subtilis artDH_13_* ^*^(Y1068H/N1233D). To mutate Asn^1233^ to Asp in *B. subtilis artDH_13_* ^*^(Y1068H), two 1.0‐kb fragments flanking the Asn^1233^ codon of *art15* (with 42‐bp overlapping at the Asn^1233^ codon (AAT), which was changed to Asp codon (GAC)) were amplified with primer pairs Nm‐L‐F/Nm‐L‐R and Nm‐R‐F/Nm‐R‐R, respectively. Successful constructions of B. subtilis art15_KS__*_ *, artDH_13_* ^*^(Y1068H), and *artDH_13_* ^*^(Y1068H/N1233D) were validated by DNA sequencing.

### Construction of the *B. subtilis* Δart21‐ech Complementation strains

The 0.9‐kb *art21‐ech* encoding fragment was PCR cloned from the genomic DNA of *B. subtilis* fmb60 with primer pair ECH‐F/ECH‐R and inserted into the *Bam*HI site of plasmid pHY300P*_aprN_* to construct pHY300P*_aprN_*::*art21‐ech*.^[^ ^21^ ^]^ The complementation strains *B. subtilis* Δ*art21‐ech/art21‐ech* was obtained by introducing pHY300P*_aprN_*::*art21‐ech* into the Δ*art21‐ech* mutant via electroporation. The complementation strains *B. subtilis* Δ*art21‐ech/art21* was constructed in similar ways as that of Δ*art21‐ech/art21‐ech*.

The fragments containing mutant genes art21*(Q280H), art21*(Q280A), art21*(Q280E), and art21*(Q280N) were amplified using pHY300P*_aprN_*::*art21* as the template with primer pairs ECH‐H‐F/ECH‐H‐R, ECH‐A‐F/ECH‐A‐R, ECH‐E‐F/ECH‐E‐R, and ECH‐N‐F/ECH‐N‐R, respectively. Four plasmids pHY300P*_aprN_*::*art21* ^*^(Q280H), pHY300P*_aprN_*::*art21* ^*^(Q280A), pHY300P*_aprN_*::*art21* ^*^(Q280E)*, and* pHY300P*_aprN_*::*art21* ^*^(Q280N) were constructed by one step cloning strategy and introduced into the Δ*art21‐ech* mutant individually via electroporation, resulting in complementation strains *B. subtilis* Δ*art21‐ech/art21* ^*^(Q280H), Δ*art21‐ech/art21* ^*^(Q280A), Δ*art21‐ech/art21* ^*^(Q280E), and Δ*art21‐ech/art21* ^*^(Q280N), respectively. The ECH‐TE like protein encoding genes from different bacteria were synthesized and cloned to plasmid pHY300P*_aprN_* by XiangHong Biotechnology Co., LTd (Beijing, China) and were introduced into the Δ*art21‐ech* mutant via electroporation to afford different complementation strains.

### Spectroscopic Analysis

HPLC analysis of fermentation product was carried out with an Apollo C18 column (5 µm, 4.6 mm × 250 mm, Alltech, IL, USA) on a Shimadzu HPLC system (Shimadzu, Kyoto, Japan). The detection wavelength was 280 nm. The column was developed with acetonitrile and water containing 0.1% formic acid at a flow rate of 1 mL min^−1^. Percentage of acetonitrile was changed using the following gradient: 0–2 min, 30%–30%; 2–32 min, 30%–80%; 32–33 min, 80%–100%; 33–38 min, 100%; 38–39 min, 100%–30%; and 39–45 min, 30%.

LC‐HRMS analyses were performed on an Agilent 1260/6460 Triple‐Quadrupole LC/MS system (Santa Clara, CA, USA) with the electrospray ionization source. NMR spectra were recorded at room temperature on a Bruker Advance 500 or 800 m NMR spectrometer (Billerica, MA, USA). MS and NMR data were analyzed using Qualitative Analysis B.07.00 and TopSpin 4.0.7, respectively.

### Production and Isolation of ATAs and Related Shunt Products

For the production of ATAs, *B. subtilis ∆art21‐ech* mutant was cultured in medium BPY (0.5% beef extract, 1.0% peptone, 0.5% yeast extract, 1% glucose, 0.5% NaCl, pH 7.0) as described.^[^ ^22^ ^]^ The well‐prepared seed culture was inoculated (3% v/v) into the production medium (Landy medium: 4.2% glucose, 1.4% L‐sodium glutamate, 0.05% MgSO_4_, 0.05% KCl, 0.1% KH_2_PO_4_, 0.000015% FeSO_4_, 0.0005% MnSO_4_, 0.000016% CuSO_4_, pH 7.0) and cultured at 33 °C, 200 rpm for 52 h. After centrifugation, the cell debris was discarded and the supernatant was extracted with equal volume of ethyl acetate for three times. The ethyl acetate fractions were concentrated *in vacuo* and subjected to a reversed‐phased C18 column (8.0 cm × 30.0 cm) eluted with acetonitrile/water gradient (20/80, 40/60, 60/40, and 80/20, v/v) sequentially. The 40/60 fraction containing ATA 1–4 was concentrated *in vacuo*, lyophilized, and further fractionated using preparative HPLC (Cartridge (PrepHT)‐C18, 5 µm, 21.2 mm × 250 mm, Agilent, CA, USA). The column was eluted with acetonitrile/water (v/v) containing 0.1% formic acid at a flow rate of 10 mL min^−1^, and the percentage of acetonitrile was changed using the following gradient: 0–40 min, 40%–55%. The 60/40 fraction containing ATA 5 and ATA 6 was treated similarly except that the preparative HPLC C‐18 column was eluted using the following gradient: 0–30 min, 50%–70%.

All compounds were further refined by semi‐preparative HPLC (Zorbax SB‐C18, 5 µm, 9.4 mm × 250 mm, Agilent, CA, USA), and the column was eluted with acetonitrile/water (v/v) containing 0.1% formic acid at a flow rate of 3.5 mL min^−1^. For ATA 1 (**5**) purification, the percentage of acetonitrile was changed using the following gradient: 0–17.5 min, 45%–49.3%. Of note, it was founded that **5** was very unstable and quickly changed to ATA 2 (**6**), which was further converted to ATA 3 (**7**) (Figure S9, Supporting Information). For the purification of **6** (2.1 mg) and **7** (3.6 mg), the percentage of acetonitrile was changed using the following gradient: 0–17 min, 43%–52.5% and 0–13 min, 47.5%–52.3%, respectively. For ATA 4 (**8**, 1.8 mg) purification, the percentage of acetonitrile was changed using the following gradient: 0–13 min, 52%–57.6%. For the purification of ATA 5 (**9**, 1.0 mg) and ATA 6 (**10**, 1.1 mg), the percentage of acetonitrile was changed using the following gradient: 0–30 min, 55%–55% and 0–20 min, 55%–61.5%, respectively.

Compounds **11**–**14** are very unstable. During isolation, all four compounds changed very quickly. Compound **13** changed to **15** and **16** within 2 h (Figure S15, Supporting Information). To obtain **15** and **16**, the ethyl acetate fractions *B. subtilis ∆art16* were concentrated *in vacuo*, subjected to a reversed‐phased C18 column (8.0 cm × 30.0 cm), and eluted with acetonitrile/water gradient (20/80, 40/60, 60/40, and 80/20, v/v). The 40/60 fraction containing **13** was collected and concentrated *in vacuo*. Most of **13** was changed to **15** and **16** during this process. Compound **15** and **16** were then purified by semi‐preparative HPLC (Cartridge (PrepHT)‐C18, 5 µm, 21.2 mm × 250 mm, Agilent, CA, USA). The column was eluted with acetonitrile/water (v/v) containing 0.1% formic acid at a flow rate of 10 mL min^−1^ using the following gradient: 0–50 min, 55% to 78%. About 1.1 mg **15** and 1.8 mg **16** were obtained.

### Isotope Labeling of **5**, **6**, and **8**

The seed culture of *B. subtilis* Δ*art21‐ech* was prepared as aforementioned. Eighteen milligrams of [1‐^13^C] sodium acetate or [2‐^13^C] sodium acetate (filter‐sterilized) was added to 0.2 L culture after culturing for 32 h, and again after culturing for 42 h in the fermentation medium. The fermentations were stopped after 52 h, and the supernatants were collected for compound isolation. Compounds **5** (0.2 and 0.1 mg), **6** (0.4 and 0.3 mg), and **8** (0.2 and 0.2 mg) isotope labeled by [1‐^13^C] and [2‐^13^C] sodium acetate, respectively, were isolated for NMR data collection.

### Structure Elucidation of **9**

All ATAs were isolated as white powder. As one of the most stable products, structure of **9** was assigned first. Compound **9** revealed a molecular formula of C_33_H_50_O_8_ with nine degrees of unsaturation from its [M + H]^+^ ion at m/z 575.3578 (C_33_H_51_O_8_ requires 575.3578) and [M + NH_4_]^+^ ion at m/z 592.3846 (C_33_H_55_N_1_O_8_ requires 592.3844) in high‐resolution electrospray ionization mass spectrometry (HR‐ESI‐MS) (Figure S13, Supporting Information). ^1^H and ^13^C NMR of **9** were collected and listed in Table S7 (Supporting Information), and the planar structure of **9** was assigned by COSY, HSQC, and HMBC NMR analyses (Figure S13, Supporting Information). The key COSY correlations 2‐CH_3_/H‐2/H‐3/H‐4/H‐5/5‐CH_3_/H‐6, H‐8/H‐9/H‐10/H‐11/H‐12/H‐13 /H‐22/H‐21/H‐16/H‐17/H‐18/18‐CH_3_/H‐19/H‐20, and H‐26/H‐25/H‐24/24‐CH_3_ connect them as separated fragments. The planar structure of **9** was constructed by key HMBC signals. Signals between C‐1/H‐2, C‐1/H‐3, C‐6/7‐CH_3_, C‐7/7‐CH_3_, and C‐8/7‐CH_3_ set C‐1 to C‐12 a triene side chain unit on C‐13, exactly consistent with the triene side chain of ARTs. Signals between C‐15/14‐CH_3_, C‐13/14‐CH_3_, and C‐14/14‐CH_3_, combining with COSY signals H‐13/H‐22/H‐21/H‐16/H‐17/H‐18/18‐CH_3_/H‐19/H‐20 set a 6/6 fused bicyclic ring system. HMBC signal between C‐27/H‐26 sets a terminal carboxyl group. HMBC signals between C‐23/H‐22, C‐23/H‐24 and C‐23/H‐25 connect the bicyclic ring with the short side chain and constructed the planar structure of **9**. ROESY was employed to establish the relative configuration of **9**. 18‐CH_3_/H‐17, H‐17/H‐21, H‐17/H‐24, and H‐13/H‐24 ROESY correlations place them on the same face of the bicyclic ring, while H‐16/H‐22 locates them on the other side of the bicyclic ring. As ATAs and ARTs were synthesized by the same assembly line, and be modified by the same set of KR and MT domains,^[^ ^23^ ^]^ the absolute configuration of **9** was proposed as Figure 2 and Figure S13 (Supporting Information) following the stereochemistry of **A**, which was verified by comparing experimental electronic circular dichroism (ECD) spectrum of **9** with its theoretical ECD calculated (Figure S13H, Supporting Information) using time‐dependent density functional theory (TDDFT).

### Computational ECD Calculation Methods

Both energy minimization and conformational analysis of **9** were carried out with the Conflex 7.0a software using built‐in MMFF94 force field.^[^ ^24^ ^]^ For ECD calculation, optimization and frequency analyses for conformers were carried out using dispersion corrected density functional theory (DFT‐D3BJ) at b3lyp/6‐311G(d, p) level with tight convergence criteria, and with IEFPCM solvent model using acetonitrile as solvent. The electronic circular dichroism (ECD) were calculated using the time‐dependent density‐functional theory (TD‐DFT) method at b3lyp/6‐311G(d, p) level with the IEFPCM solvent (acetonitrile) model.^[^ ^25^ ^]^ All of the optimization and frequency analyses for ECD calculation of conformers were executed with Gaussian9 software. The Boltzmann distribution and Gibbs free energy at 298.15 K of conformers were generated according to the analyses of Gaussian9 output files by Shermo 2.3 software.^[^ ^26^ ^]^ Experimental ECD spectrum of **9** in acetonitrile was compared with its theoretical ECD, which conformed it's absolute configuration, indicating that ATAs inherit the stereochemistry features of ARTs.

### Structure Elucidation of **8**

Compound **8** revealed a molecular formula of C_37_H_54_O_10_ with eleven degrees of unsaturation from its [M+H]^+^ ion at m/z 659.3793 (C_37_H_55_O_10_ requires 659.3790) in HR‐ESI‐MS (Figure S12, Supporting Information). The structure of **8** was assigned maily based on its NMR data (Table S6 and Figure S12, Supporting Information). The key COSY correlations 2‐CH_3_/H‐2/H‐3/H‐4/H‐5/5‐CH_3_/H‐6, H‐8/H‐9/H‐10/H‐11/H‐12/H‐13/H‐22/H‐21/H‐16/H‐17/H‐18/18‐CH_3_/H‐19/H‐20, and H‐26/H‐25/H‐24/24‐CH_3_ connect them as separated fragments. The planar structure of **8** was constructed by key HMBC signals. Signals between C‐1/H‐2, C‐1/H‐3, C‐6/7‐CH_3_, C‐7/7‐CH_3_, and C‐8/7‐CH_3_ set C‐1 to C‐12 as a triene side chain. Signals between C‐15/14‐CH_3_, C‐13/14‐CH_3_, and C‐14/14‐CH_3_, combining with COSY signals set a 6/6 fused bicyclic ring system. HMBC signals between C‐27/H‐26, C‐27/H‐25, C‐27/H‐28, and C‐29/H‐28 set a lactone ring. HMBC signals between C‐27/H‐30 and C‐30/H‐28 set the carboxymethyl branch at C‐27. And, the signals between C‐23/H‐22, C‐23/H‐24, and C‐23/H‐25 connect the bicyclic ring with the lactone ring. ROESY was employed to establish the relative configuration of **8**. The 18‐CH_3_/H‐17, 18‐CH_3_/H‐21, H‐17/H‐21, and H‐13/H‐21 ROESY correlations place them on the same face of the bicyclic ring, while H‐18/H‐16, H‐16/H‐22, and H‐12/H‐22 ROESY correlations locate them at the other side of the bicyclic ring. Since compounds **8** and the other ATAs are synthesized by the same PKS machinery for **9** and ARTs, it is proposed that they have the same configuration as **9**.

### Structure Elucidation of **6**

Compound **6** revealed a molecular formula of C_37_H_52_O_9_ with twelve degrees of unsaturation from its [M+H]^+^ ion at m/z 641.3681 (C_37_H_53_O_9_ requires 641.3684) and [M+NH_4_]^+^ ion at m/z 658.3953 (C_37_H_57_N_1_O_9_ requires 658.3950) in HR‐ESI‐MS (Figure S10, Supporting Information). ^1^H and ^13^C NMR data of **6** were listed in Table S4 (Supporting Information). The planar structure of **6** was assigned by COSY, HSQC, HMBC NMR analyses (Figure S10, Supporting Information). When comparing with the NMR data of compound **8**, it is clearly noted differences of the chemical shifts of C‐27 (155.71 ppm), C‐28 (117.46 ppm), and H‐28 (5.92 ppm, s) that set a double bond between C‐27 and C‐28.

### Structure Elucidation of **5**

Compound **5** revealed a molecular formula of C_37_H_54_O_10_ with eleven degrees of unsaturation from its [M+H]^+^ ion at m/z 659.3790 (C_37_H_55_O_10_ requires 659.3790) and [M+NH_4_]^+^ ion at m/z 676.4054 (C_37_H_59_N_1_O_10_ requires 676.4055) in HR‐ESI‐MS (Figure S8, Supporting Information). It was very unstable during the separation and purification processes, it underwent a spontaneous lactonization to generate **6**, which further underwent a slowly auto‐decarboxylation to afford **7** (Figure S9, Supporting Information). Luckily, the NMR data of **5** samples (purified at low temperature, dark, and under nitrogen protection) of ^13^C isotope label feeding experiments allowed to assign its structure. ^1^H and ^13^C NMR data of **5** were listed in Table S3 (Supporting Information). The planar structure of **5** was assigned by COSY, HSQC, and HMBC NMR data (Figure S8, Supporting Information). Compared with **6**, **5** has two more proton and one more oxygen (C_37_H_52_O_9_), NMR data also supports that the lactone ring is not formed in **5**.

### Structure Elucidation of **7**

Compound **7** revealed a molecular formula of C_36_H_52_O_7_ with eleven degrees of unsaturation from its [M+H]^+^ ion at m/z 597.3785 (C_36_H_53_O_7_ requires 597.3786) and [M+NH_4_]^+^ ion at m/z 614.4051 (C_36_H_57_N_1_O_7_ requires 614.4051) in HR‐ESI‐MS (Figure S11, Supporting Information). ^1^H and ^13^C NMR of **7** were listed in Table S5 (Supporting Information). The planar structure of **7** was further assigned by COSY, HSQC, and HMBC NMR analyses (Figure S11, Supporting Information) and compared with the NMR data of compound **6**. The only difference between of **7** and **6** is that **7** lacks the C‐31 carboxyl group, which is supported by the C‐30 (21.65 ppm) and H‐30 (2.02 ppm, s, 3H) signals of a methyl group.

### Structure Elucidation of **10**

Compound **10** revealed a molecular formula of C_30_H_46_O_5_ with eight degrees of unsaturation from its [M+H]^+^ ion at m/z 487.3418 (C_30_H_47_O_5_ requires 487.3418) in HR‐ESI‐MS (Figure S14, Supporting Information). ^1^H and ^13^C NMR of **10** were listed in Table S8 (Supporting Information). The planar structure of **10** was assigned by COSY, HSQC, and HMBC NMR analyses (Figure S14, Supporting Information). **10** has a shorter side chain ending at C‐25. The key COSY correlations H‐25/H‐24 and HMBC signals between C‐23/H‐22, C‐23/H‐24, and C‐23/H‐25 connect the bicyclic ring with the short side chain.

### Structure Elucidation of **15**

Compound **15** revealed a molecular formula of C_28_H_42_O_6_ with eight degrees of unsaturation from its [M+H]^+^ ion at m/z 475.3058 (C_28_H_43_O_6_ requires 475.3054) in HR‐ESI‐MS (Figure S16, Supporting Information). ^1^H and ^13^C NMR of **15** were collected and listed in Table S9 (Supporting Information). The planar structure of **15** was assigned by further analysis on COSY, HSQC, and HMBC NMR data (Figure S16, Supporting Information). The key COSY correlations 2‐CH_3_/H‐2/H‐3/H‐4/H‐5/5‐CH_3_/H‐6, H‐8/H‐9/H‐10/H‐11/H‐12/H‐13 and H‐15/H‐16/H‐17/H‐18/18‐CH_3_/H‐19/H‐20/H‐21/H‐22 connect them as separated fragments. The planar structure of **15** was then constructed by the key HMBC signals. Signals between C‐1/H‐2, C‐1/H‐3, C‐6/7‐CH_3_, C‐7/7‐CH_3_, C‐8/7‐CH_3_, C‐13/14‐CH_3_, C‐14/14‐CH_3_, and C‐15/14‐CH_3_ set C‐1 to C‐23 as a long pentaene fatty acid chain. Specifically, the molecular formula of **15** (C_28_H_42_O_6_) is 18 Da less than the proposed intermediate **13**, implying a spontaneous dehydration of **15**. ROESY correlations between 2‐H and 7‐CH_3_ suggests that this dehydration takes place between C‐1 and C‐7 and forms a lactone ring. The relative configuration of **15** was established by ROESY correlations between H‐2/7‐CH_3_ and 5‐CH_3_/7‐CH_3_, which is consistent with the configuration of the side chains of ARTs and ATAs.

### Structure Elucidation of **16**

Compound **16** revealed a molecular formula of C_28_H_42_O_6_ with eight degrees of unsaturation from its [M+H]^+^ ion at m/z 475.3055 (C_28_H_43_O_6_ requires 475.3054) in HR‐ESI‐MS (Figure S17, Supporting Information). ^1^H and ^13^C NMR of **16** were listed in Table S10 (Supporting Information). The COSY, HSQC, and HMBC NMR analyses (Figure S17, Supporting Information) suggested that **16** had the same planar structure as **15**. The only difference between **16** and **15** was that the configurations at C‐7, which was supported by the correlation between H‐5/7‐CH_3_ in the ROESY data of **16**.

### Protein Expression and Purification

The 1.8‐kb *art21* gene was amplified using the *B. subtilis* fmb60 genome as a template with primer pair 28‐21‐F/28‐21‐R. It was inserted into the *Nde*I and *Bam*HI sites of pET28a to afford pET28a::*art21*. The point mutation plasmid pET28a::*art21* ^*^Q280H was obtained by PCR amplification with primer pair ECH‐H‐F/ECH‐H‐R using plasmid pET28a::*art21* as a template. The plasmids were verified by DNA sequencing and transformed into *E. coli* BL21 (DE3). The resultant recombinant strains were inoculated into LB medium with 50 µg mL^−1^ kanamycin and cultured overnight at 37 °C, 220 rpm. The seed culture was used to inoculate LB with 50 µg mL^−1^ kanamycin at 1% ratio (v/v) and grown at 37 °C, 220 rpm until OD_600_ reached 0.6. Expression of *N*‐His_6_‐tagged Art21 and Art21^*^Q280H were induced by the addition of 100 µm isopropyl‐*β*‐thiogalactoside (IPTG) and further cultured at 16 °C,160 rpm for 16 h.

The cells were harvested by centrifugation (4 °C, 4000 rpm, 30 min) and re‐suspended in lysis buffer (5 mm imidazole, 20 mm Tris‐HCl, 500 mm NaCl, pH 7.9). After lysed by ultrasonication (5 s break, 7 s pause, 30 min in total) in an ice bath, the cell debris was removed by centrifugation (4 °C, 10 000 rpm, 30 min). The supernatant was loaded onto a Ni‐NTA affinity column equilibrated with lysis buffer, washed with washing buffer (60 mm imidazole, 20 mm Tris‐HCl, 500 mm NaCl, pH 7.9), and followed by elution buffer (500 mm imidazole, 20 mm Tris‐HCl, 500 mm NaCl, pH 7.9). The target collection was desalted and exchanged to HEPEs buffer (20 mm HEPEs, 100 mm NaCl, 20% glycerol, pH 7.9) using a PD‐10 column. Finally, the purified protein was concentrated by centrifugal filter (Millipore, MA, USA) and stored at −80 °C with 20% glycerol. Protein concentrations were measured with a BCA protein assay kit (Takara, Dalian, China).

The 2.4‐kb *art6* gene was amplified using the *B. subtilis* fmb60 genome as a template with primer pair 28‐6‐F/28‐6‐R and was inserted into the *Nde*I and *Bam*HI restriction sites of pET28a to afford pET28a::*art6*. The 0.8‐kb *art19* gene and 0.8‐kb *art20* gene were amplified using primer pairs 28‐19‐F/28‐19‐R and 28‐20‐F/28‐20‐R, respectively. Plasmids pET28a::*art19* and pET28a::*art20* were constructed in a similar way to pET28a::*art6*. The expression and purification procedures of *N*‐His_6_‐tagged Art6, Art19, and Art20 were the same as those of Art21. The other two proteins, Art2 and ACP_13_, were expressed and purified as described.^[^ ^18^ ^]^

### In Vitro Assays of ECH_1_ and ECH_2_

The 50 µL reaction mixture consists of Tris‐HCl buffer (20 mm Tris‐HCl, 150 mm NaCl, 10% glycerol, pH 7.5), 1 mm HMG‐CoA, 5 µm Art19, 5 µm Art20 or Art21 or Art21^*^(Q280H). The reaction was incubated at 37 °C for 10 h before it was quenched by adding an equal volume of acetonitrile. After centrifugation, the supernatant was subjected to LC‐MS analysis. The reaction mixture containing PksH (ECH_1_ with dehydration activity) and PksI (ECH_2_ with decarboxylation activity) were utilized as positive controls.

HPLC analyses were carried out with an Apollo C18 column (5 µm, 4.6 mm × 250 mm, Alltech, IL, USA) on a Shimadzu HPLC system (Shimadzu, Kyoto, Japan). The column was developed with acetonitrile and water containing 0.1% formic acid at a flow rate of 1 mL min^−1^. Percentage of acetonitrile was changed using the following gradient: 0–35 min, 5%–25%; 35–36 min, 25%–100%; 36–45 min, 100%; 45–46 min, 100%–5%; 46–55 min, 5%. The detection wavelength was 254 nm. LC‐HRMS analysis was performed on an Agilent 1260/6460 Triple‐Quadrupole LC/MS system (Santa Clara, CA, USA) with the electrospray ionization source.

### In Vitro Assays of Art21 Toward acyl Transfer Reactions

To test the influence of Art21 on related acyl transfer reactions, the 50 µL rection mixtures containing 20 mm HEPEs (pH 7.9), 1 mm MgCl_2_, 5 µm Art2 or Art6, 5 µm Art21, 100 µm holo‐ACP_13_, 150 µm malonyl‐CoA or 150 µm succinyl‐CoA were incubated at 30 °C for 2 h. Two standard samples, malonyl‐ACP_13_ and succinyl‐ACP_13_, were prepared by loading phosphopantetheinylated malonyl and succinyl onto apo‐ACP_13_ using Sfp (a phosphopantetheinyl transferase with high substrate promiscuity from *B. subtilis*), respectively.^[^ ^26^ ^]^ The assays with boiled Art21 was utilized as negative controls. After incubation, the mixture was immediately cooled on ice and subjected with HPLC analysis.

### HPLC Analysis of the Art21 Assays

HPLC detections were carried out using an Epic C18 column (5 µm, 4.6 mm × 250 mm, Alltech, IL, USA) on a Shimadzu HPLC system (Shimadzu, Kyoto, Japan). The column was developed using mobile phases consisting of water with 0.1% trifluoroacetic acid (solvent A) and acetonitrile with 0.1% trifluoroacetic acid (solvent B) at a flow rate of 0.4 mL min^−1^. The ratio of solvent B was changed as the following gradient: 0–45 min, 50%–54%; 45–46 min, 54–100%; 46–60 min, 100%; 60–62 min, 100–50%; and 62–75 min, 50%. Samples were monitored at 210 nm.

### Intact Protein Mass Analysis of ACP_13_ Loading

After lyophilization, samples were re‐dissolved in water containing 0.1% formic acid. The mass spectrometry analysis of different samples was performed with Orbitrap Fusion Tribrid mass spectrometer (Thermo Fisher Scientifc, MA, USA). About 1 µg of protein was loaded onto a trap column (C18, 3 µm particles, 150 µm ID, 3 cm length, Dr. Maisch GmbH) and separated using an analytical column (C18, 1.9 µm particles, 150 µm ID, 15 cm length, Dr. Maisch GmbH) at a flow rate of 400 nL min^−1^ with a 45 min LC gradient composed of Solvent A (water with 0.1% formic acid (v/v)) and Solvent B (acetonitrile with 0.1% formic acid (v/v)). The gradient was 20–80% B for 40 min, and finally 100% B for 5 min at a flow rate of 400 nL min^−1^. The proteins eluting from the column were ionized using a NSI source (positive ion: 2200 V, ion transfer tube temperature: 320 °C, maximum injection time: 100 ms) in positive‐ion mode. The data was acquired in the Orbitrap at a resolution setting of 15 000 within scan range *m/z* 800–3000. The monoisotopic masses for intact proteins were determined as previously descried,^[^ ^27^ ^]^ the raw data was deconvoluted with the built‐in function of Xcalibur 4.3 (Thermo Fisher Scientifc, MA, USA) within 1017–1669 *m/z* mass range. The conversion rates for ACP_13_ loading are estimated using the following formula: Area_acyl‑ACP13_/(Area_holo‑ACP13_+ Area_acyl‑ACP13_).

### Antibacterial Assays

The antibacterial assays of ARTs and ATAs were performed with a micro‐broth dilution method in the 96 wells culture plate according to the protocol from the Standard of National Committee for Clinical Laboratory.^[^ ^28^ ^]^ Erythromycin was used as a positive control. The tested bacteria in this assay were incubated in Mueller‐Hinton (MH) broth and normalized to an optical density of 10^6^ colony forming units (CFU) mL^−1^. The detected substances were diluted using the double‐dilution method in 96‐well plates of MH broth. After incubation at 37 °C for 20 h, the minimal inhibitory concentration (MIC) was defined as the minimum concentration of the antibacterial substance at which no growth of the tested organisms could be detected.

### Protein Structural Prediction and Molecular Docking

AlphaFold2 (ColabFold v1.5.5) was adopted to predict the structures of Art20 and Art21‐ECH, and results unrelaxed_rank_001 were picked for further analysis.^[^ ^29^ ^]^ Structure alignment and comparison was performed with Pymol 2.5.5. The receptor structures of Art20 and Art21 were optimized using molecular dynamics simulations. The initial ligand position in the complex conformation was selected as the docking site, with dimensions of 22 Å (enclosing box) and 10 Å (ligand diameter midpoint box). Other parameters were set to default. The ligand was processed with LigPrep, optimized using the OPLS4 force field, and kept neutral. Molecular docking was performed using Glide at standard precision, with the receptor kept rigid and the ligand flexible, using default settings. For constrained docking, additional Position/NOE constraints were applied at His^240^ (Art20) and Gln^280^ (Art21), with minimum and maximum NOE distances set to 1 and 5.5 Å, respectively. Due to the polarity of His^240^ and Gln^280^, polar interactions between the residues and the ligand (as donor or acceptor) were specified. Other settings remained consistent with unconstrained docking.

## Conflict of Interest

The authors declare no conflict of interest.

## Supporting information

Supporting Information

## Data Availability

The data that support the findings of this study are available in the supplementary material of this article.
