# Supplementary material for: Reprogramming of the Aurantinin Polyketide Assembly Line to Synthesize Auritriacids by Excising an Atypical Enoyl‐CoA Hydratase Domain
Source: Adv Sci (Weinh). 2024 Jul 12;11(35):2401708. doi: 10.1002/advs.202401708 (PMC11425284; doi:10.1002/advs.202401708)
Supplement: Supplementary file 1 — Supporting Information [file ADVS-11-2401708-s001.pdf]

Supporting Information

Reprogramming of the Aurantinin Polyketide Assembly Line to Synthesize Auritriacids by Excising an Atypical Enoyl-CoA Hydratase Domain

Dacheng Wang, [‡] *Huijin Mao,* [‡] *Zelian Zhao, Lilu Liu, Yihua Chen* and Pengwei Li**

Content

Table S1. Bacterial strains and plasmids. 4

Table S2. Primers used in this study. 5

Table S3. NMR data of ATA 1 (**5**), ^{13}C NMR (125 M Hz) and ^{1}H NMR (500 M Hz) in MeOH-*d*$_4$. 7

Table S4. NMR data of ATA 2 (**6**), ^{13}C NMR (125 M Hz) and ^{1}H NMR (500 M Hz) in MeOH-*d*$_4$. 8

Table S5. NMR data of ATA 3 (**7**), ^{13}C NMR (125 M Hz) and ^{1}H NMR (500 M Hz) in MeOH-*d*$_4$. 9

Table S6. NMR data of ATA 4 (**8**), ^{13}C NMR (125 M Hz) and ^{1}H NMR (500 M Hz) in MeOH-*d*$_4$. 10

Table S7. NMR data of ATA 5 (**9**), ^{13}C NMR (125 M Hz) and ^{1}H NMR (500 M Hz) in MeOH-*d*$_4$. 11

Table S8. NMR data of ATA 6 (**10**), ^{13}C NMR (125 M Hz) and ^{1}H NMR (500 M Hz) in MeOH-*d*$_4$. 12

Table S9. NMR data of compound **15**, ^{13}C NMR (125 M Hz) and ^{1}H NMR (500 M Hz) in DMSO-*d*$_6$. 13

Table S10. NMR data of compound **16**, ^{13}C NMR (200 M Hz) and ^{1}H NMR (800 M Hz) in DMSO-*d*$_6$. 14

Table S11. The conversion rates of malonyl- and succinyl-ACP$_{13}$ in different assays. 15

Table S12. Percent Identity (%) between Art KS domains. 16

Table S13. Percent Identity (%) between Art DH domains. 17

Table S14. Percent Identity (%) between Art MT domains. 17

Table S15. Percent Identity (%) between Art ACP domains. 18

Table S16. Information of the ECH$_Q$-TE proteins. 19

Figure S1. Sequence analysis of Art ACP domains. 20

Figure S2. Sequence analysis of Art19. 21

Figure S3. Sequence analysis of Art20 and Art21-ECH. 21

Figure S4. Construction of *B. subtilis* Δ*art19*, Δ*art20*, Δ*art21*, and Δ*art16*. 22

Figure S5. The enzymatic assays of ECH$_1$ and ECH$_2$. 23

Figure S6. Comparison of the UV-visible spectroscopy data of ARTs and ATAs. 24

Figure S7. Construction of *B. subtilis* Δ*artDH*$_{13}$, Δ*art21-ech*, and Δ*art21-te*. 25

Figure S8. Spectral data of ATA 1 (**5**). 26

Figure S9. Evaluation of the stability of compound **5**. 32

Figure S10. Spectral data of ATA 2 (**6**). 33

Figure S11. Spectral data of ATA 3 (**7**). 40

Figure S12. Spectral data of ATA 4 (**8**). 45

Figure S13. Spectral data of ATA 5 (**9**). 52

Figure S14. Spectral data of ATA 6 (**10**). 59

Figure S15. Evaluation of the stability of compound **13**. 65

Figure S16. Spectral data of **15**. 66

Figure S17. Spectral data of **16**. 72

Figure S18. Absorbance and HR-ESI-MS data of compounds **11**-**14**. 78

Figure S19. Comparison of the active sites of Art20 and Art21-ECH$_Q$ domain. 79

Figure S20. HPLC profiles of the *B. subtilis* wild-type and different mutant strains under both 280 nm and 340 nm. 80

Figure S21. SDS-PAGE analyses of Art2,Art6, ACP$_{13}$,Art19,Art20,Art21,Art21*(Q280H),PksH and PksI. 81

Figure S22. Intact protein mass spectra data of the enzymatic assays that load different acyl-CoA to holo-ACP$_{13}$ by Art2 and Art6 with or without the presence of Art21. 80

Figure S23. Multiple sequence alignment of Art DH domains. 82

Figure S24. Multiple Sequence alignment of the single domain ECH proteins. 83

Figure S25. Multiple Sequence alignment of the ECH$_Q$-TE proteins. 84

Figure S26. The biosynthetic gene clusters that contain an ECH$_Q$-TE encoding gene. 85

Figure S27. Multiple sequence alignment of DH domains from the 23 biosynthetic gene clusters with an ECH_Q-TE encoding gene 87
Figure S28. Phylogenetic analysis of the acyltransferases from the 23 biosynthetic gene clusters with an ECH_Q-TE encoding gene. ... 88
References 89

Table S1. Bacterial strains and plasmids.

**Strains or plasmids**	**Characteristics***	**Reference or source**
Escherichia coli		
DH5α	General cloning host	Invitrogen
BL21 (DE3)	Host for protein expression	Novagen
Bacillus		
B. subtilis fmb60	Aurantinins producing wild-type strain	1
**Plasmids**		
pRN5101	Erm^r, Amp^r, used for inactivation of genes or TE domain	2
pHY300P$_{aprN}$	Tet^r, Amp^r, used for expressing genes in *B. subtilis* fmb60	2
pHY300P$_{aprN}$::*art21-ech*	Tet^r, Amp^r, used for complementing *art21-ech* to mutant Δ*art21-ech*	This work
pHY300P$_{aprN}$::*art21*	Tet^r, Amp^r, used for complementing gene *art21* to mutant Δ*art21-ech*	This work
pHY300P$_{aprN}$::*art21**(Q280H)	Tet^r, Amp^r, used for complementing gene *art21**(Q280H) to mutant Δ*art21-ech*	This work
pHY300P$_{aprN}$::*art21**(Q280A)	Tet^r, Amp^r, used for complementing gene *art21**(Q280A) to mutant Δ*art21-ech*	This work
pHY300P$_{aprN}$::*art21**(Q280N)	Tet^r, Amp^r, used for complementing gene *art21**(Q280N) to mutant Δ*art21-ech*	This work
pHY300P$_{aprN}$::*art21**(Q280E)	Tet^r, Amp^r, used for complementing gene *art21**(Q280E) to mutant Δ*art21-ech*	This work
pET28a	Kan^r, protein production vector	Novagen
pET28a::*art6*	Kan^r, for producing *N*-His$_6$-tagged Art6	This work
pET28a::*art19*	Kan^r, for producing *N*-His$_6$-tagged Art19	This work
pET28a::*art20*	Kan^r, for producing *N*-His$_6$-tagged Art20	This work
pET28a::*art21*	Kan^r, for producing *N*-His$_6$-tagged Art21	This work
pET28a::*art21**Q280H	Kan^r, for producing *N*-His$_6$-tagged Art21*Q280H	This work

**Erm^r, erythromycin resistance; Amp^r, ampicillin resistance; Tet^r, tetracycline resistance; Kan^r, kanamycin resistance.*

Table S2. Primers used in this study.

Primers	Sequences (5′ to 3′)$^{\#}$	Uses
19-L-F	gactgcgcaaaagacataatcgatAAGCTTgctgaaggcggtgaagacttaac	Amplification of the upstream region of
19-L-R	gagacgaagcagatgcttacggcactcctcaatcatccggtcat	*art19* (*Hin*dIII)
19-R-F	atgaccggatgattgaggagtgccgtaagcatctgcttcgtctc	Amplification of the downstream region
19-R-R	taactgtgataaactaccgcattaAAGCTTtcctcttcctcacctctggttg	of *art19* (*Hin*dIII)
20-L-F	taactgtgataaactaccgcattaAAGCTTggcgaagtcgttgagtaaggag	Amplification of the upstream region of
20-L-R	ctaacacctccactctaggcaagacaatctctggctccaactcttg	*art20* (*Hin*dIII)
20-R-F	caagagttggagccagagattgtcttgcctagagtggaggtgttag	Amplification of the downstream region
20-R-R	gactgcgcaaaagacataatcgatAAGCTTtgccaactccttcgccatctt	of *art20* (*Hin*dIII)
21-L-F	taaactaccgcattaaagcttccgttaactaaaagggcaatc	Amplification of the upstream region of
21-L-R	ctcctcatatatatttttaatccatatctggcattccctc	*art21*(*Hin*dIII)
21-R-F	gagggaatgccagatatggattaaaaaatatatatgaggag	Amplification of the downstream region
21-R-R	gacataatcgataagctttacatgaccatgcgaagggccg	of *art21* (*Hin*dIII)
16-L-F	taaactaccgcattaAAGCTTgagccgctgttgatagacacc	Amplification of the upstream region of
16-L-R	gatctcaccagcctgtacccgttccaactctaccat	*art16* (*Hin*dIII)
16-R-F	atggtagagttggaacgggtacaggctggtgagatc	Amplification of the upstream region of
16-R-R	gacataatcgatAAGCTTctcgcaatctctcaacgcctg	*art16* (*Hin*dIII)
ECH-L-F	cactatggcgtgctGCTAGCgcgttaattgatggcctgcttga	Amplification of the upstream region of
ECH-L-R	ctacctcggttaaagcttccttctgcatggtcacctgaacaatcc	*art21-ech* (*Nhe*I)
ECH-R-F	ggattgttcaggtgaccatgcagaaggaagctttaaccgaggtag	Amplification of the downstream region
ECH-R-R	tcaacgcatatagcGCTAGCatagtgatctcatctgcatccg	of *art21-ech* (*Nhe*I)
DH-L-F	taaactaccgcattaAAGCTTgtggaagcccatggcacaggc	Amplification of the upstream region of
DH-L-R	gctaaatgcagtcttactgcgacttaaaagcggctcccttcc	*art15DH* (*Hin*dIII)
DH-R-F	ggaagggagccgcttttaagtcgcagtaagactgcatttagc	Amplification of the upstream region of
DH-R-R	gacataatcgatAAGCTTggcatggagcacatttgccgc	*art15DH* (*Hin*dIII)
TE-L-F	taaactaccgcattaAAGCTT gagaggagggaatgccagatatg	Amplification of the upstream region of
TE-L-R	ctcctcatatatatttttaatcaaaagccggcaacatttt	*art21TE* (*Hin*dIII)
TE-R-F	aaaatgttgccggcttttgattaaaaaatatatatgaggag	Amplification of the downstream region
TE-R-R	gacataatcgatAAGCTT tacatgaccatgcgaagggccg	of *art21TE* (*Hin*dIII)
TEm-L-F	cactatggcgtgctGCTAGCggattgttcaggtgaccatgcag	Amplification of the upstream region of
TEm-L-R	taagccaatacacctcctaaGGCataaccgcctaaatcatagg	Ser^{390} codon of *art21* (*Nhe*I)
TEm-R-F	cctatgatttaggcggttatGCCttaggaggtgtattggctta	Amplification of the downstream
TEm-R-R	tcaacgcatatagcGCTAGCcaccgcctgatcaataatccgt	Ser^{390} codon of *art21* (*Nhe*I)
KSm-L-F	cactatggcgtgctGCTAGCgagcaggttcgaccatcatt	Amplification of the upstream region of
KSm-L-R	ggacagagaccaaagaggaggaGGCgaccgtatcaatggccatgct	Cys^{595} codon of *art15* (*Nhe*I)
KSm-R-F	agcatggccattgatacggtcGCCtcctcctctttggtctctgtcc	Amplification of the downstream Cys^{595}
KSm-R-R	tcaacgcatatagcGCTAGCcacacgttcttccaatgcg	codon of *art15* (*Nhe*I)
Ym-L-F	taaactaccgcattaAAGCTTgagaagcagtattgtgcc	Amplification of the upstream region of
Ym-L-R	ggctggttgccccttgaccatGTGttcacttaaaagcggctc	Tyr^{1068} codon of *art15* (*Hin*dIII)
Ym-R-F	gagccgcttttaagtgaaCACatggtcaaggggcaaccagcc	Amplification of the downstream region
Ym-R-R	gacataatcgatAAGCTTcgtatcaatccgagcaatagc	of Tyr^{1068} codon of *art15* (*Hin*dIII)
Nm-L-F	taaactaccgcattaAAGCTTcttgaggacgataatcgcctg	Amplification of the upstream region of
Nm-L-R	cgggcggcctgtaaagaagcGTCtagcagaacaggttggg	Asn^{1233} codon of *art15* (*Hin*dIII)

Nm-R-F	cccaacctgttctgctaGACgcttctttacaggccgcccg	Amplification of the downstream region
Nm-R-R	gacataatcgatAAGCTTgaggatatctggtaacgagcg	of Asn[1233] codon of *art15* (*Hin*dIII)
ECH-F	aaaggagagggtaaagaGGATCCatggatacaaaggcgatatt	Amplification of the *art21-ech* (*Bam*HI)
ECH-R	gagatctgcaggtcgacGGATCCttaatcaaaagccggcaacatttt	
21-F	aaaggagagggtaaagaGGATCCatggatacaaaggcgatatt	Amplification of *art21* or art*21-te*[*]
21-R	gagatctgcaggtcgacGGATCCttataagtcatacagttttt	(*Bam*HI)
ECH-H-F	tgggcgatGCAcgagaaaacc	Point mutation to generate gene
ECH-H-R	ggttttctcgTGCatcgccca	*art21*[*](Q280H)
ECH-A-F	tgggcgatgGCAgagaaaacc	Point mutation to generate gene
ECH-A-R	ggttttctcTGCcatcgccca	*art21*[*](Q280A)
ECH-E-F	tgggcgatgGAAgagaaaacc	Point mutation to generate gene
ECH-E-R	ggttttctcTTCcatcgccca	*art21*[*](Q280E)
ECH-N-F	tgggcgatgAATgagaaaacc	Point mutation to generate gene
ECH-N-R	ggttttctcATTcatcgccca	*art21*[*](Q280N)
28-6-F	gtgccgcgcggcagcCATATGccaacaaccccgatg	Amplification of *art6* (*Nde*I and *Bam*HI)
28-6-R	cggagctcgaattcGGATCCtcatgtaatagtttgaaatg	
28-19-F	gtgccgcgcggcagcCATATGgactatcaaacggtgaat	Amplification of *art19* (*Nde*I and *Bam*HI)
28-19-R	acggagctcgaattcGGATCCtcactcccatggaaacttccc	
28-20-F	gtgccgcgcggcagcCATATGgtgacatctgttgtacat	Amplification of *art20* (*Nde*I and *Bam*HI)
28-20-R	acggagctcgaattcGGATCCtcattttccaaataagttatt	
28-21-F	tgccgcgcggcagcCATATGccagatatggatacaaag	Amplification of *art21* (*Nde*I and *Bam*HI)
28-21-R	cggagctcgaattcGGATCCttataagtcatacagtttttc	

[#] *The designed restriction site in each primer is capitalized.*

Table S3. NMR data of ATA 1 (**5**), ^{13}C NMR (125 M Hz) and ^{1}H NMR (500 M Hz) in MeOH-d_4.

No.	δ_C (ppm)	δ_H (ppm)
1	177.69	
2	46.48	2.39 (p, J = 7.0 Hz, 1H)
2-CH_3	11.68	1.20 (d, J = 7.0 Hz, 3H)
3	69.76	3.88 (m, 1H)
4	41.91	1.12 (m, 1H), 1.51 (m, 1H)
5	27.24	1.98 (m, 1H)
5-CH_3	17.85	0.90 (d, J = 5.8 Hz, 3H)
6	48.62	2.06 (m, 1H), 1.98 (m, 1H)
7	137.99	
7-CH_3	15.34	1.79 (s, 3H)
8	126.49	5.86 (d, J = 11.0 Hz, 1H)
9	128.75	6.45 (dd, J = 14.7, 11.0 Hz, 1H)
10	129.83	6.05 (dd, J = 14.7, 10.7 Hz, 1H)
11	134.97	6.24 (dd, J = 14.8, 10.7 Hz, 1H)
12	131.83	5.53 (dd, J = 14.8, 10.3 Hz, 1H)
13	45.72	3.37 (m, 1H)
14	134.31	
14-CH_3	21.19	1.72 (s, 3H)
15	121.27	5.65 (s, 1H)
16	44.59	2.54 (m, 1H)
17	77.49	3.29 (m, 1H)
18	34.81	1.37 (m, 1H)
18-CH_3	17.56	0.99 (d, J = 6.4 Hz, 3H)
19	33.50	1.66 (m, 1H), 0.99 (m, 1H)
20	23.35	1.96 (m, 1H), 1.49 (m, 1H)
21	35.40	2.14 (m, 1H)
22	55.03	3.33 (m, 1H)
23	213.99	
24	50.19	3.08 (m, 1H)
24-CH_3	9.03	0.96 (d, J = 6.7 Hz, 3H)
25	72.85	4.07 (dt, J = 9.9, 2.8 Hz, 1H)
26	36.51	2.98 (dd, J = 13.0, 2.0 Hz, 1H), 2.58 (dd, J = 13.0, 10.2 Hz, 1H)
27	153.80	
28	120.74	5.94 (s, 1H)
29	168.32	
30	43.84	3.33 (m, 2H)
31	172.76	

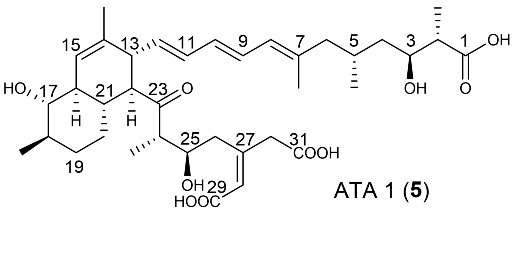

ATA 1 (**5**)

Table S4. NMR data of ATA 2 (**6**), ^{13}C NMR (125 M Hz) and ^{1}H NMR (500 M Hz) in MeOH-d_4.

No.	δ_C (ppm)	δ_H (ppm)
1	177.64	
2	46.41	2.39 (p, J = 7.0 Hz, 1H)
2-CH_3	11.72	1.19 (d, J = 7.0 Hz, 3H)
3	69.76	3.87 (ddd, J = 9.9, 6.5, 2.1 Hz, 1H)
4	41.91	1.11 (m, 1H), 1.49 (m, 1H)
5	27.28	1.97 (m, 1H)
5-CH_3	17.98	0.88 (d, J = 5.8 Hz, 3H)
6	48.66	2.05 (m, 1H), 1.95 (m, 1H)
7	138.22	
7-CH_3	15.51	1.78 (s, 3H)
8	126.51	5.85 (d, J = 11.1 Hz, 1H)
9	129.02	6.45 (dd, J = 14.7, 11.1 Hz, 1H)
10	129.77	6.06 (dd, J = 14.7, 10.7 Hz, 1H)
11	134.97	6.24 (dd, J = 14.8, 10.7 Hz, 1H)
12	131.82	5.54 (dd, J = 14.8, 10.5 Hz, 1H)
13	45.54	3.37 (m, 1H)
14	134.12	
14-CH_3	21.34	1.71 (s, 3H)
15	121.35	5.66 (s, 1H)
16	44.49	2.53 (m, 1H)
17	77.38	3.26 (m, 1H)
18	34.78	1.37 (m, 1H)
18-CH_3	17.67	0.97 (d, J = 6.4 Hz, 3H)
19	33.47	1.65 (m, 1H), 0.97 (m, 1H)
20	23.37	1.91 (m, 1H), 1.47 (m, 1H)
21	35.40	2.13 (m, 1H)
22	56.58	3.25 (m, 1H)
23	212.39	
24	46.31	3.37 (m, 1H)
24-CH_3	10.76	1.02 (d, J = 6.9 Hz, 3H)
25	79.39	4.63 (dt, J = 14.8, 10.7 Hz, 1H)
26	29.44	2.52 (m, 1H), 2.43 (m, 1H)
27	155.71	
28	117.46	5.92 (s, 1H)
29	165.23	
30	40.81	3.37 (m, 1H), 3.33 (m, 1H)
31	171.09	

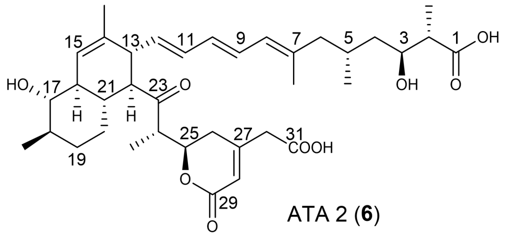

ATA 2 (**6**)

Table S5. NMR data of ATA 3 (**7**), ^{13}C NMR (125 M Hz) and ^{1}H NMR (500 M Hz) in MeOH-d_4.

No.	δ_C (ppm)	δ_H (ppm)
1	177.62	
2	46.45	2.37 (p, J = 6.9 Hz, 1H)
2-CH_3	11.67	1.19 (d, J = 7.0 Hz, 3H)
3	69.75	3.87 (m, 1H)
4	41.92	1.12 (m, 1H), 1.50 (m, 1H)
5	27.24	1.98 (m, 1H)
5-CH_3	17.85	0.88 (d, J = 6.1 Hz, 3H)
6	48.62	2.06 (m, 1H), 1.97 (m, 1H)
7	138.16	
7-CH_3	15.34	1.78 (s, 3H)
8	126.46	5.86 (d, J = 11.1 Hz, 1H)
9	128.97	6.46 (dd, J = 14.7, 11.1 Hz, 1H)
10	129.73	6.06 (dd, J = 14.7, 10.6 Hz, 1H)
11	135.00	6.25 (dd, J = 14.9, 10.6 Hz, 1H)
12	131.79	5.54 (dd, J = 14.9, 10.5 Hz, 1H)
13	45.53	3.38 (m, 1H)
14	134.12	
14-CH_3	21.16	1.72 (s, 3H)
15	121.29	5.66 (s, 1H)
16	44.52	2.53 (m, 1H)
17	77.36	3.26 (m, 1H)
18	34.76	1.36 (m, 1H)
18-CH_3	17.54	0.97 (d, J = 6.4 Hz, 3H)
19	33.44	1.66 (m, 1H), 0.98 (m, 1H)
20	23.34	1.91 (m, 1H), 1.49 (m, 1H)
21	35.41	2.13 (m, 1H)
22	56.63	3.25 (m, 1H)
23	212.49	
24	46.27	3.37 (m, 1H)
24-CH_3	10.65	1.02 (d, J = 6.9 Hz, 3H)
25	79.19	4.60 (m, 1H)
26	30.46	2.45 (m, 1H), 2.29 (m, 1H)
27	160.39	
28	114.72	5.79 (s, 1H)
29	165.79	
30	21.65	2.02 (s, 3H)

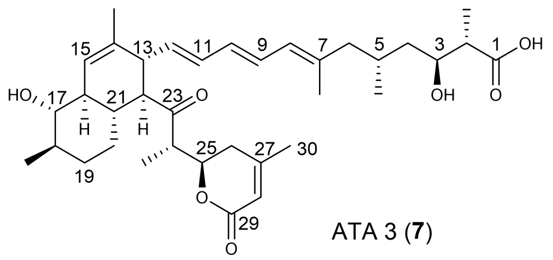

ATA 3 (**7**)

Table S6. NMR data of ATA 4 (**8**), ^{13}C NMR (125 M Hz) and ^{1}H NMR (500 M Hz) in MeOH-d_4.

No.	δ_C (ppm)	δ_H (ppm)
1	177.64	
2	46.44	2.38 (p, J = 6.9 Hz, 1H)
2-CH_3	11.68	1.19 (d, J = 7.0 Hz, 3H)
3	69.76	3.87 (ddd, J = 9.2, 6.6, 2.4 Hz, 1H)
4	41.91	1.12 (ddd, J = 13.5, 9.6, 3.1 Hz, 1H), 1.48 (m, 1H)
5	27.26	1.97 (m, 1H)
5-CH_3	17.88	0.88 (d, J = 6.1 Hz, 3H)
6	48.63	2.05 (m, 1H), 1.96 (m, 1H)
7	138.15	
7-CH_3	15.39	1.78 (s, 3H)
8	126.48	5.86 (d, J = 11.1 Hz, 1H)
9	128.97	6.46 (dd, J = 14.7, 11.2 Hz, 1H)
10	129.74	6.05 (dd, J = 14.7, 10.7 Hz, 1H)
11	134.94	6.24 (dd, J = 14.8, 10.7 Hz, 1H)
12	131.73	5.54 (dd, J = 14.8, 10.5 Hz, 1H)
13	45.54	3.36 (m, 1H)
14	134.14	
14-CH_3	21.21	1.71 (s, 3H)
15	121.29	5.65 (s, 1H)
16	44.56	2.52 (m, 1H)
17	77.38	3.26 (dd, J = 10.5, 5.3 Hz, 1H)
18	34.77	1.35 (m, 1H)
18-CH_3	17.57	0.97 (d, J = 6.4 Hz, 3H)
19	33.44	1.66 (m, 1H), 0.97 (m, 1H)
20	23.32	1.92 (m, 1H), 1.47 (m, 1H)
21	35.45	2.11 (m, 1H)
22	56.21	3.21 (dd, J = 6.7, 2.3 Hz, 1H)
23	212.41	
24	46.92	3.30 (dd, J = 6.9, 5.0 Hz, 1H)
24-CH_3	10.25	1.00 (d, J = 6.9 Hz, 3H)
25	78.43	3.87 (ddd, J = 12.2, 5.0, 3.2 Hz, 1H)
26	35.14	2.00 (m, 1H), 1.85 (dd, J = 13.9, 12.2 Hz, 1H)
27	67.57	
28	41.49	2.75 (d, J = 17.7 Hz, 1H), 2.70 (d, J = 17.7 Hz, 1H)
29	171.20	
30	45.17	2.59 (s, 2H)
31	172.46	

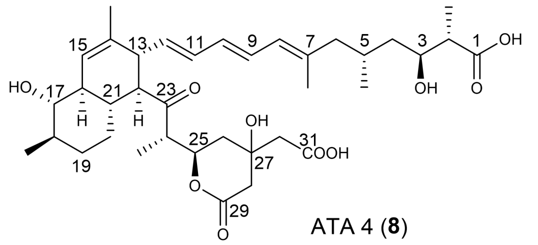

ATA 4 (**8**)

Table S7. NMR data of ATA 5 (**9**), ^{13}C NMR (125 M Hz) and ^{1}H NMR (500 M Hz) in MeOH-d_4.

No.	δ_C (ppm)	δ_H (ppm)
1	177.71	
2	46.48	2.37 (m, 1H)
2-CH_3	11.68	1.19 (d, *J* = 7.0 Hz, 3H)
3	69.76	3.87 (ddd, *J* = 9.4, 6.7, 2.1 Hz, 1H)
4	41.91	1.12 (m, 1H), 1.48 (m, 1H)
5	27.24	1.97 (m, 1H)
5-CH_3	17.86	0.88 (d, *J* = 6.0 Hz, 3H)
6	48.61	2.05 (m, 1H), 1.96 (m, 1H)
7	138.04	
7-CH_3	15.33	1.78 (s, 3H)
8	126.47	5.85 (d, *J* = 11.1 Hz, 1H)
9	128.81	6.43 (dd, *J* = 14.7, 11.1 Hz, 1H)
10	129.78	6.04 (dd, *J* = 14.7, 10.7 Hz, 1H)
11	134.86	6.20 (dd, *J* = 14.8, 10.7 Hz, 1H)
12	131.76	5.52 (dd, *J* = 14.8, 9.5 Hz, 1H)
13	45.72	3.33 (m, 1H)
14	134.23	
14-CH_3	21.17	1.71 (s, 3H)
15	121.32	5.65 (s, 1H)
16	44.62	2.53 (m, 1H)
17	77.47	3.27 (dd, *J* = 10.5, 5.3 Hz, 1H)
18	34.80	1.36 (m, 1H)
18-CH_3	17.55	0.98 (d, *J* = 6.4 Hz, 3H)
19	33.49	1.66 (m, 1H), 0.98 (m, 1H)
20	23.33	1.93 (m, 1H), 1.47 (m, 1H)
21	35.40	2.14 (m, 1H)
22	55.30	3.31 (m, 1H)
23	213.93	
24	48.57	3.12 (m, 1H)
24-CH_3	9.32	0.91 (d, *J* = 6.9 Hz, 3H)
25	69.55	4.27 (dt, *J* = 8.8, 4.4 Hz, 1H)
26	38.34	2.50 (dd, *J* = 15.7, 4.4 Hz, 1H),<br>2.31 (dd, *J* = 15.7, 8.8 Hz, 1H)
27	174.08	

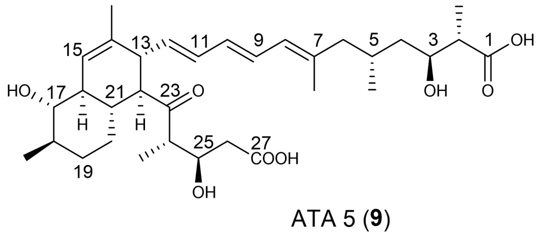

ATA 5 (**9**)

Table S8. NMR data of ATA 6 (**10**), ^{13}C NMR (125 M Hz) and ^{1}H NMR (500 M Hz) in MeOH-d_4.

No.	δ_C (ppm)	δ_H (ppm)
1	177.65	
2	46.47	2.37 (m, 1H)
2-CH_3	11.67	1.19 (d, J = 7.0 Hz, 3H)
3	69.75	3.87 (ddd, J = 9.4, 6.7, 2.1 Hz, 1H)
4	41.92	1.11 (m, 1H), 1.49 (m, 1H)
5	27.24	1.97 (m, 1H)
5-CH_3	17.82	0.88 (d, J = 6.0 Hz, 3H)
6	48.61	2.05 (m, 1H), 1.96 (m, 1H)
7	137.98	
7-CH_3	15.31	1.78 (s, 3H)
8	126.47	5.85 (d, J = 11.1 Hz, 1H)
9	128.72	6.44 (dd, J = 14.7, 11.1 Hz, 1H)
10	129.82	6.07 (dd, J = 14.7, 10.6 Hz, 1H)
11	134.82	6.21 (dd, J = 14.8, 10.6 Hz, 1H)
12	131.44	5.51 (dd, J = 14.8, 10.4 Hz, 1H)
13	45.66	3.27 (m, 1H)
14	134.22	
14-CH_3	21.21	1.71 (s, 3H)
15	121.29	5.65 (s, 1H)
16	44.55	2.52 (m, 1H)
17	77.46	3.26 (dd, J = 10.5, 5.4 Hz, 1H)
18	34.81	1.35 (m, 1H)
18-CH_3	17.53	0.98 (d, J = 6.4 Hz, 3H)
19	33.48	1.66 (dq, J = 9.9, 3.2 Hz, 1H), 0.98 (m, 1H)
20	23.45	1.92 (m, 1H), 1.47 (m, 1H)
21	35.50	2.11 (m, 1H)
22	55.18	3.03 (dd, J = 6.8, 2.3 Hz, 1H)
23	213.36	
24	33.85	2.58 (dq, J = 17.6, 7.3 Hz, 1H)<br>2.41 (m, 1H)
25	15.31	0.96 (t, J = 7.3 Hz, 3H)

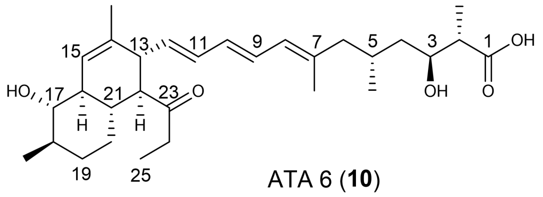

ATA 6 (**10**)

Table S9. NMR data of compound **15**, ^{13}C NMR (125 M Hz) and ^{1}H NMR (500 M Hz) in DMSO-d_6.

No.	δ_C (ppm)	δ_H (ppm)
1	176.26	
2	45.20	2.26 (m, 1H)
2-CH_3	12.80	1.08 (d, J = 6.8 Hz, 3H)
3	72.29	3.61 (ddd, J = 11.6, 6.8, 1.5 Hz, 1H)
4	37.70	0.80 (m, 1H), 1.47 (m, 1H)
5	26.79	1.57 (m, 1H)
5-CH_3	22.52	0.85 (d, J = 6.3 Hz, 3H)
6	43.74	0.93 (m, 1H), 1.81 (m, 1H)
7	75.12	
7-CH_3	31.46	1.13 (s, 3H)
8	138.89	5.63 (d, J = 15.4 Hz, 1H)
9	130.47	6.21 (m, 1H)
10	128.10	6.29-6.31 (m, 1H)
11	133.19	6.29-6.31 (m, 1H)
12	132.39	6.29-6.31 (m, 1H)
13	137.62	6.29-6.31 (m, 1H)
14	133.35	
14-CH_3	12.69	1.81 (s, 3H)
15	132.30	6.10 (d, J = 11.2 Hz, 1H)
16	125.42	6.36 (dd, J = 14.8, 11.2 Hz, 1H)
17	142.44	5.67 (dd, J = 14.8, 6.9 Hz, 1H)
18	36.99	2.19 (m, 1H)
18-CH_3	20.70	0.97 (d, J = 6.7 Hz, 3H)
19	32.80	1.27 (m, 1H), 1.42 (m, 1H)
20	35.05	1.30 (m, 1H), 1.34 (m, 1H)
21	67.74	3.73 (m, 1H)
22	43.22	2.15 (m, 1H), 2.22 (m, 1H)
23	174.19	

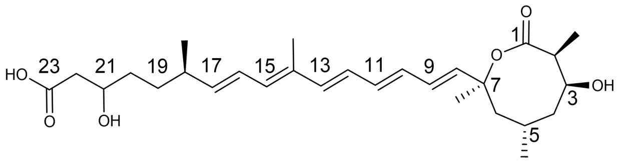

15

Table S10. NMR data of compound **16**, ^{13}C NMR (200 M Hz) and ^{1}H NMR (800 M Hz) in DMSO-d_6.

No.	δ_C (ppm)	δ_H (ppm)
1	175.77	
2	44.92	2.34 (p, J = 6.9, 1H)
2-CH_3	12.62	1.08 (d, J = 6.9, 3H)
3	70.97	3.77 (m, 1H)
4	37.54	0.79 (m, 1H), 1.54 (m, 1H)
5	25.67	1.82 (m, 1H)
5-CH_3	22.44	0.87 (d, J = 6.5, 3H)
6	43.44	0.90 (m, 1H), 1.53 (m, 1H)
7	73.68	
7-CH_3	21.44	1.22 (s, 3H)
8	142.06	5.76 (d, J = 15.2, 1H)
9	126.37	6.21 (dd, J = 15.2, 9.9, 1H)
10	128.07	6.30 (m, 1H)
11	133.03	6.30 (m, 1H)
12	132.44	6.29 (m, 1H)
13	137.29	6.31 (m, 1H)
14	133.26	
14-CH_3	12.58	1.82 (s, 3H)
15	132.07	6.10 (d, J = 11.2, 1H)
16	125.36	6.37 (dd, J = 14.7, 11.2, 1H)
17	142.17	5.67 (dd, J = 14.7, 7.9, 1H)
18	36.84	2.20 (m, 1H)
18-CH_3	20.59	0.98 (d, J = 6.6, 3H)
19	32.67	1.28 (m, 1H), 1.43 (m, 1H)
20	34.93	1.29 (m, 1H), 1.36 (m, 1H)
21	67.46	3.78 (m, 1H)
22	42.89	2.22 (dd, J = 14.8, 7.9, 1H)<br>2.30 (dd, J = 14.8, 5.1, 1H)
23	173.21	

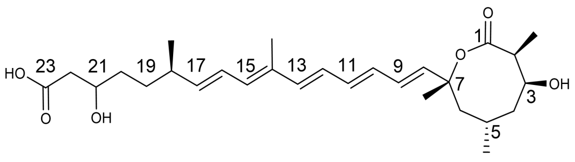

16

Table S11. The conversion rates of malonyl- and succinyl-ACP_{13} in different assays.

Samples	Conversion (%)	
	**Malony-ACP_{13}**	**Succinyl-ACP_{13}**
holo-ACP_{13}+Art2+M-CoA	6	ND
holo-ACP_{13}+Art2+M-CoA+Art21	3	ND
holo-ACP_{13}+Art2+S-CoA	ND	93
holo-ACP_{13}+Art2+S-CoA+Art21	ND	87
holo-ACP_{13}+Art2+M-CoA+S-CoA	ND	71
holo-ACP_{13}+Art2+M-CoA+S-CoA+Art21	ND	61
holo-ACP_{13}+Art6+M-CoA	94	ND
holo-ACP_{13}+Art6+M-CoA+Art21	82	ND
holo-ACP_{13}+Art6+S-CoA	ND	0

ND: not detected.

Table S12. Percent Identity (%) between Art KS domains.

PI(%)	$ArtKS_2$	$ArtKS_3$[a]	$ArtKS_4$	$ArtKS_5$	$ArtKS_6$[b]	$ArtKS_8$	$ArtKS_9$	$ArtKS_{10}$	$ArtKS_{11}$	$ArtKS_{12}$	$ArtKS_{13}$[c]	$ArtKS_{14}$
$ArtKS_1$	53.05	43.46	49.84	50.81	46.15	45.08	42.77	43.65	41.95	39.22	45.42	50.34
$ArtKS_2$		48.10	54.12	50.71	45.56	43.55	42.17	44.81	44.37	37.00	41.23	67.94
$ArtKS_3$			45.86	46.57	42.69	39.95	40.78	47.48	41.72	33.17	42.11	50.12
$ArtKS_4$				53.32	45.60	46.64	46.88	48.10	48.74	39.57	47.95	53.21
$ArtKS_5$					47.56	47.33	46.19	47.16	47.02	37.83	46.22	50.48
$ArtKS_6$						58.54	67.81	41.72	48.65	37.53	55.81	43.53
$ArtKS_8$							56.71	43.84	46.14	35.20	52.85	43.09
$ArtKS_9$								43.75	50.11	35.96	58.12	42.03
$ArtKS_{10}$									42.66	32.54	42.14	44.68
$ArtKS_{11}$										36.01	47.98	42.53
$ArtKS_{12}$											34.56	36.56
$ArtKS_{13}$												44.32
$ArtKS_{14}$												

[a]: *$ArtKS_3$ have the same sequence with $ArtKS_{15}$.*

[b]: *$ArtKS_6$ have the same sequence with $ArtKS_7$.*

[c]: *Data of $ArtKS_{13}$ were highlighted in red.*

Table S13. Percent Identity (%) between Art DH domains.

PI(%)	$ArtDH_6$[a]	$ArtDH_8$	$ArtDH_{10}$	$ArtDH_{12}$	$ArtDH_{13}$[b]
$ArtDH_5$	34.20	31.58	33.33	22.63	25.77
$ArtDH_6$		37.73	40.00	23.88	28.19
$ArtDH_8$			30.00	23.91	26.78
$ArtDH_{10}$				22.16	26.29
$ArtDH_{12}$					20.81

[a]: *$ArtDH_6$ have same sequence with $ArtDH_7$.*

[b]: *Data of $ArtDH_{13}$ were highlighted in red.*

Table S14. Percent Identity (%) between Art MT domains.

PI(%)	$ArtMT_7$	$ArtMT_9$	$ArtMT_{13}$[a]
$ArtMT_1$	48.85	53.00	52.53
$ArtMT_7$		51.61	53.69
$ArtMT_9$			50.23

[a]: *Data of $ArtMT_{13}$ were highlighted in red.*

Table S15. Percent Identity (%) between Art ACP domains.

PI(%)	ACP_1	ACP_2[a]	ACP_3	$ACP_{4\text{-}1}$	$ACP_{4\text{-}2}$	ACP_5	ACP_6	ACP_7	ACP_8	ACP_9	ACP_{10}	ACP_{11}	ACP_{12}	ACP_{13}[b]	$ACP_{15\text{-}1}$	$ACP_{15\text{-}2}$
Art_{10}-ACP	26.98	15.38	23.91	29.23	25.53	16.67	14.10	10.26	22.50	35.56	17.95	19.23	23.08	37.25	33.33	21.79
ACP_1		19.51	26.15	32.31	25.00	32.35	32.35	26.76	23.53	40.00	27.87	30.30	23.94	38.10	27.91	22.95
ACP_2			28.79	26.56	26.15	26.56	25.00	32.81	34.38	23.21	38.71	32.61	23.44	28.79	28.21	27.78
ACP_3				46.15	50.77	19.23	17.95	20.51	17.95	32.31	23.81	12.82	23.91	31.75	45.24	43.08
$ACP_{4\text{-}1}$					51.56	21.79	64.29	29.82	30.77	32.31	26.98	32.08	36.00	30.77	40.00	40.91
$ACP_{4\text{-}2}$						15.38	16.67	30.51	19.23	33.87	25.00	53.85	21.15	28.08	42.31	51.56
ACP_5							68.92	34.29	51.43	23.08	20.51	20.51	24.36	28.57	14.10	16.67
ACP_6								35.71	54.29	33.33	27.87	30.30	35.29	33.33	17.95	16.67
ACP_7									42.86	33.33	32.26	30.30	57.89	21.79	11.54	14.10
ACP_8										28.57	34.43	40.48	39.22	34.15	16.67	17.95
ACP_9											28.07	32.20	17.95	46.15	30.61	27.69
ACP_{10}												32.65	29.41	29.51	30.00	37.25
ACP_{11}													29.23	35.09	26.09	23.33
ACP_{12}														28.57	23.81	23.33
ACP_{13}															36.36	32.79

[a]: *ACP_2 have same sequence with ACP_{14}.*

[b]: *Data of $ArtMT_{13}$ were highlighted in red.*

Table S16. Information of the ECH_Q-TE proteins.

ECH_Q-TE	Source strain	Accession number	Percent identity (%)
Art21	*Bacillus subtilis* fmb60	OEI73471	100.00
Bsu_9	*Bacillus subtilis* subsp. *spizizenii* DV1-B-1	WP_019713719	99.50
Bsu_952	*Bacillus subtilis* At3	QJC87839	99.17
Bsi_6	*Bacillus siamensis* RGM 2529	WP_076983040	98.01
Bsu_21355	*Bacillus subtilis* HU Biol-II	PPA34060	98.18
Bin_913	*Bacillus inaquosorum* A65.1	WP_134981026	98.18
BinF2_6	*Bacillus inaquosorum* EC30O5B-F2-1	WP_268397699	98.01
BinF1_6	*Bacillus inaquosorum* EC49O5B-F1	WP_268275686	98.01
BinJ4_6	*Bacillus inaquosorum* EC30O1B-J4	WP_268290803	98.01
BinD21_6	*Bacillus inaquosorum* EC49O2B-D21	WP_268273328	97.84
Bsp_6	*Bacillus sp.* XF8	WP_207994781	83.17
Mal_5	*Melghirimyces algeriensis* DSM 45474	WP_185955937	83.06
GdiDD1_6	*Gracilibacillus dipsosauri* DD1	WP_181397615	77.74
Gdi_6	*Gracilibacillus dipsosauri* bin82	WP_277679337	77.74
Tsp_6	*Thermoactinomyces sp.* CICC 10522	WP_198065021	66.61
Tda_13	*Thermoactinomyces daqus* s-11	WP_160173857	66.45
Tsp10522_6	*Thermoactinomyces sp.* CICC 10523	WP_198056182	66.45
Dar_43	*Dictyobacter arantiisoli* Uno17	WP_172631837	48.07
Bba_12775	*Bacillota bacterium* HOT.CON.82	MCL6590816	50.38
Mha_282	*Marininema halotolerans* DSM 45789	WP_091833648	49.02
Cru_19	*Cellulosilyticum ruminicola* JCM 14822	WP_083461233	41.38
Apo_127	*Anaeromicropila populeti* 743A	WP_177214584	41.05
Lsp_9	uncultured *Lachnoclostridium* sp. SRR1747057_bin.44	WP_297933286	42.86

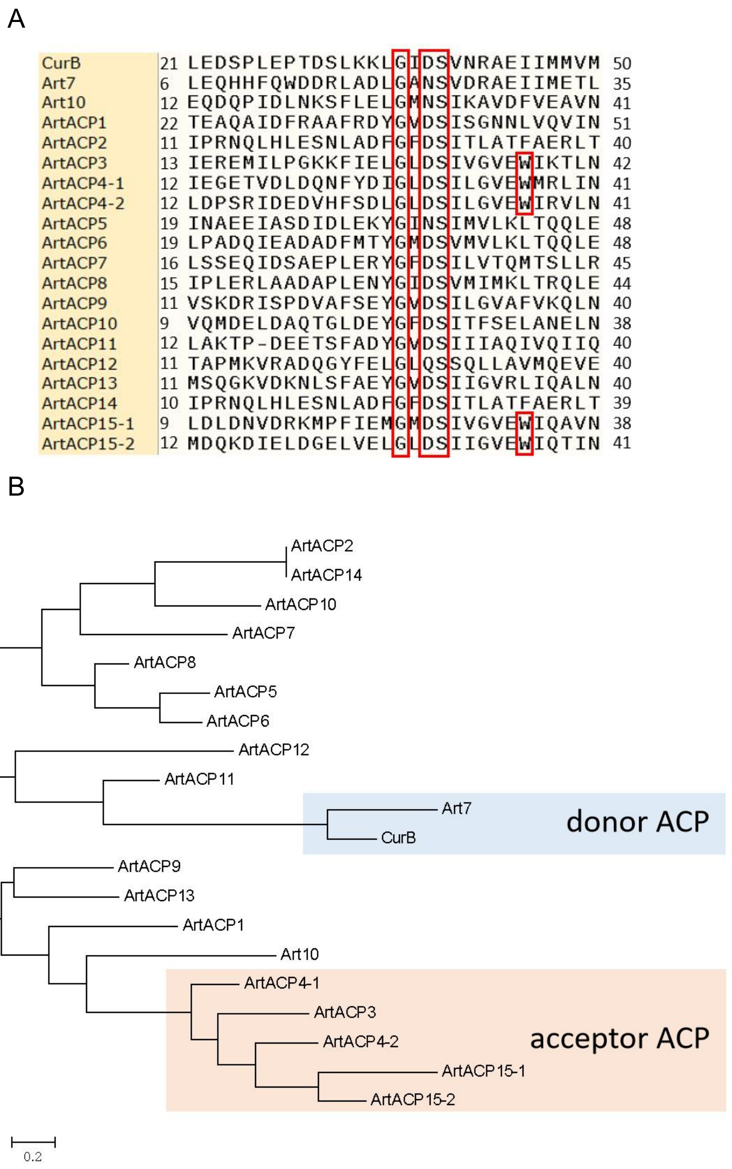

Figure S1. Sequence analysis of Art ACP domains. (**A**) Multiple sequence alignment and (**B**) phylogenetic analysis of the Art ACP domains. GXDSX$_5$W is a conserved motif in the β-branching acceptor ACPs (indicated with red box),[3] and CurB is a well-characterized donor ACP of β-branching.[4]

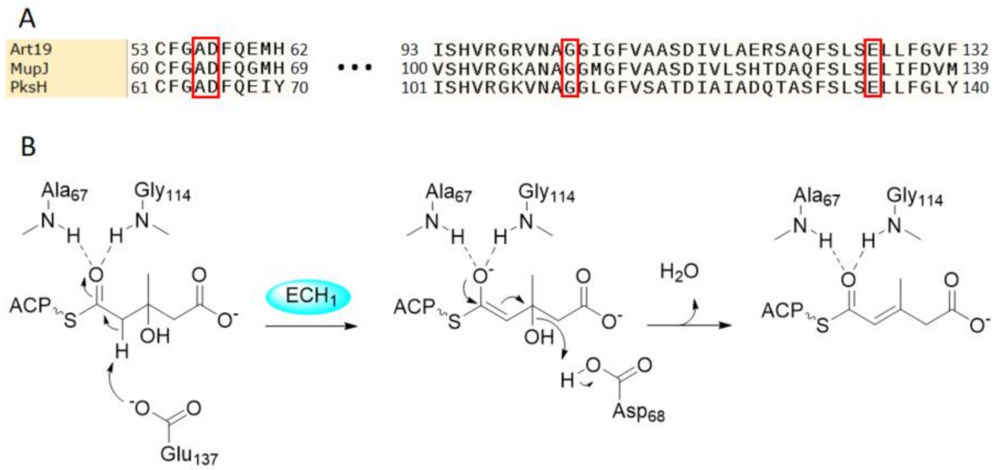

Figure S2. Sequence analysis of Art19. (**A**) Multiple sequence alignment of Art19 with typical ECH_1 (MupJ and PksH). The crucial catalytic residues were indicated with red boxes; (**B**) The dehydration mechanism of ECH_1 (using PksH as an example) in β-branching process.[5]

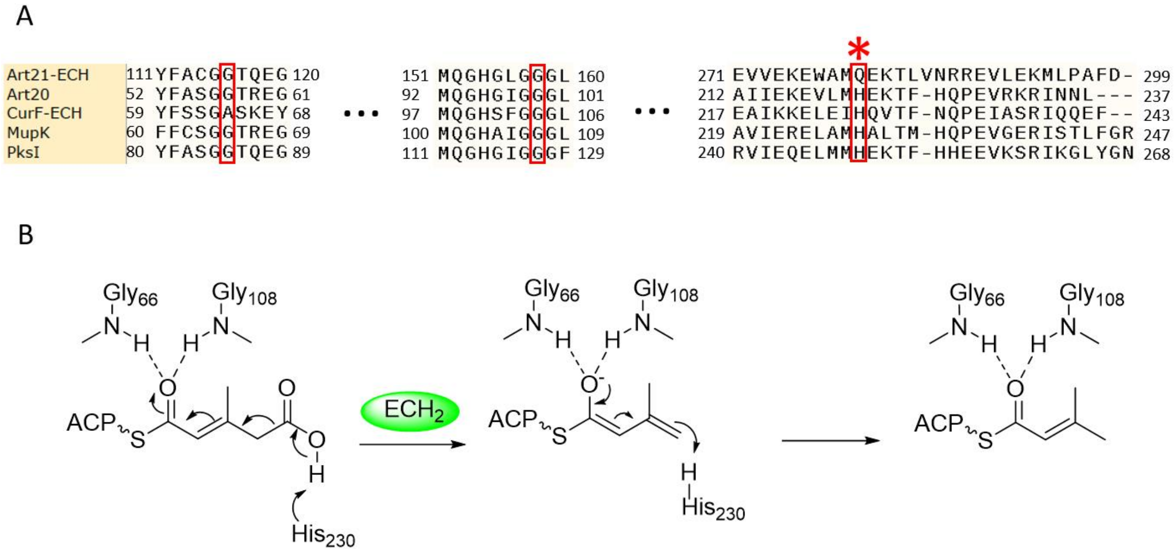

Figure S3. Sequence analysis of Art20 and Art21-ECH. (**A**) Multiple sequence alignment of Art20 and Art21-ECH with typical ECH_2 (CurF-ECH, MupK, and PksI). The key catalytic residues were indicated with red boxes; (**B**) The decarboxylation mechanism of ECH_2 (using PksI as an example) in β-branching process.[5]

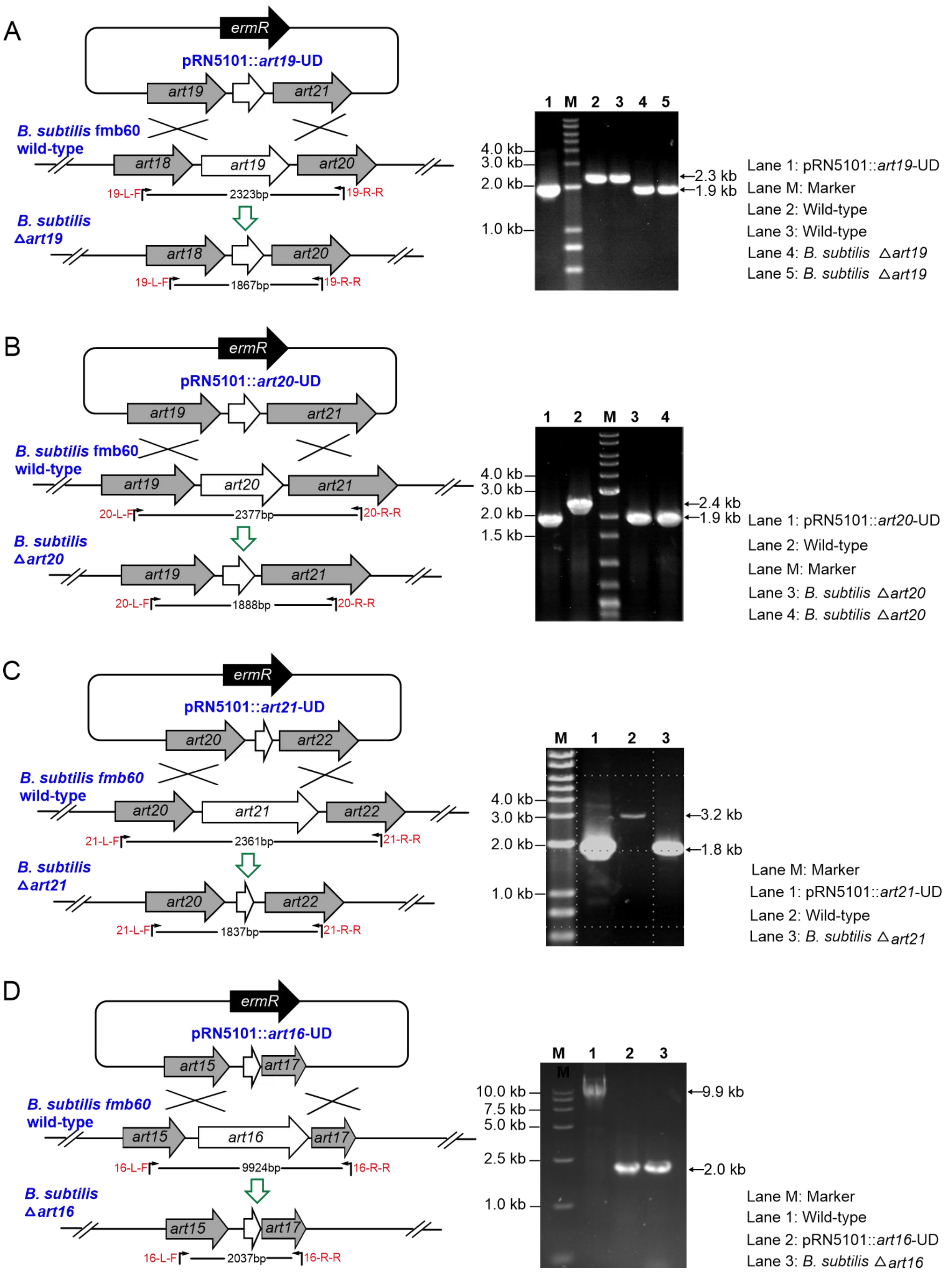

Figure S4. Construction of *B. subtilis* Δ*art19*, Δ*art20*, Δ*art21*, and Δ*art16*. Construction of *B. subtilis* Δ*art19* (**A**), *B. subtilis* Δ*art20* (**B**), *B. subtilis* Δ*art21* (**C**), and *B. subtilis* Δ*art16* (**D**) were performed using a gene in-frame deletion strategy. The primers used for genotype verification of the mutants were indicated with red and the sizes of the PCR products were also shown.

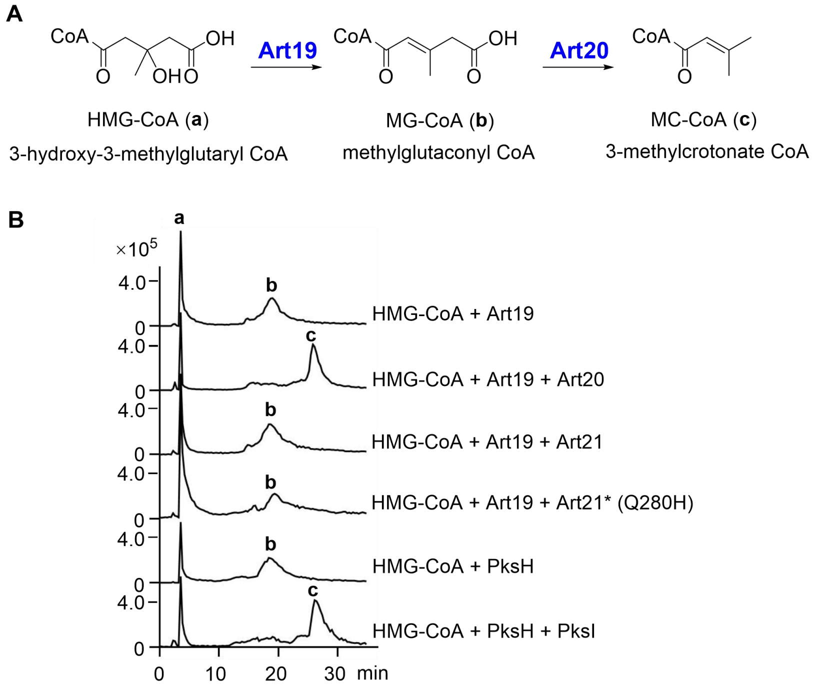

Figure S5. The enzymatic assays of ECH_1 and ECH_2. (**A**) Reaction scheme for conversion of HMG-CoA (**a**) to MG-CoA (**b**) and then MC-CoA (**c**); (**B**) LCMS analysis of assays using HMG-CoA as the substrates. The well-characterized PksH and PksI were used as positive controls of ECH_1 and ECH_2.

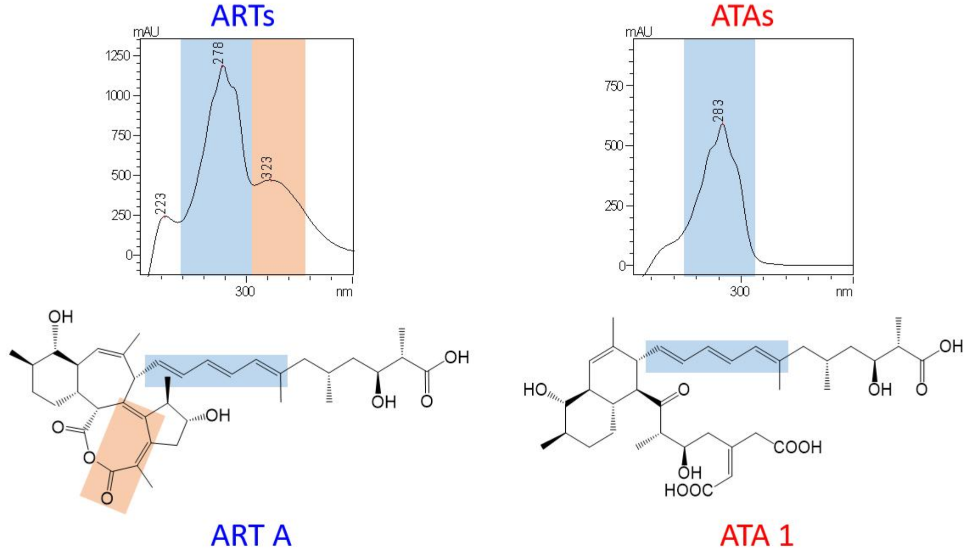

Figure S6. Comparison of the UV-visible spectroscopy data of ARTs and ATAs. ARTs have the characteristic UV absorptions of triene (at about 280nm, in blue) and conjugation moiety (at about 320nm, in orange), while ATAs only possesses the UV absorptions of triene (at about 280nm, in blue).

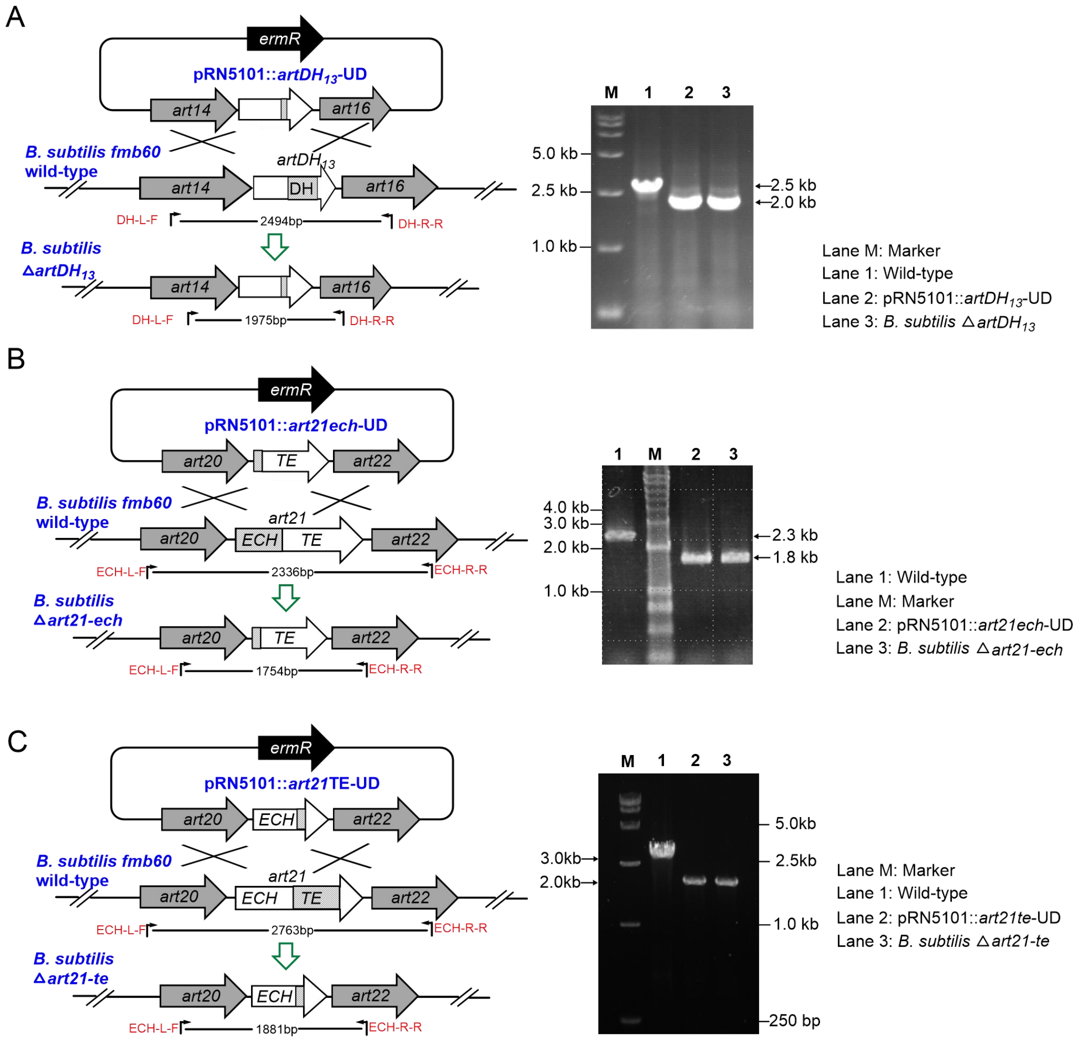

Figure S7. Construction of *B. subtilis* Δ*artDH*$_{13}$, Δ*art21-ech*, and Δ*art21-te*. Construction of *B. subtilis* Δ*artDH*$_{13}$ (**A**), *B. subtilis* Δ*art21-ech* (**B**) and *B. subtilis* Δ*art21-te* (**C**) were performed using an in-frame deletion strategy. The primers used for genotype verification of the mutants were indicated with red and the sizes of the PCR products were also shown.

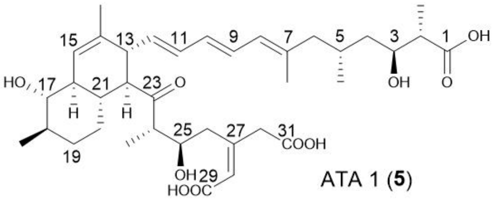

A

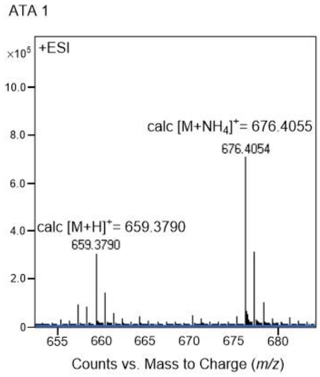

B

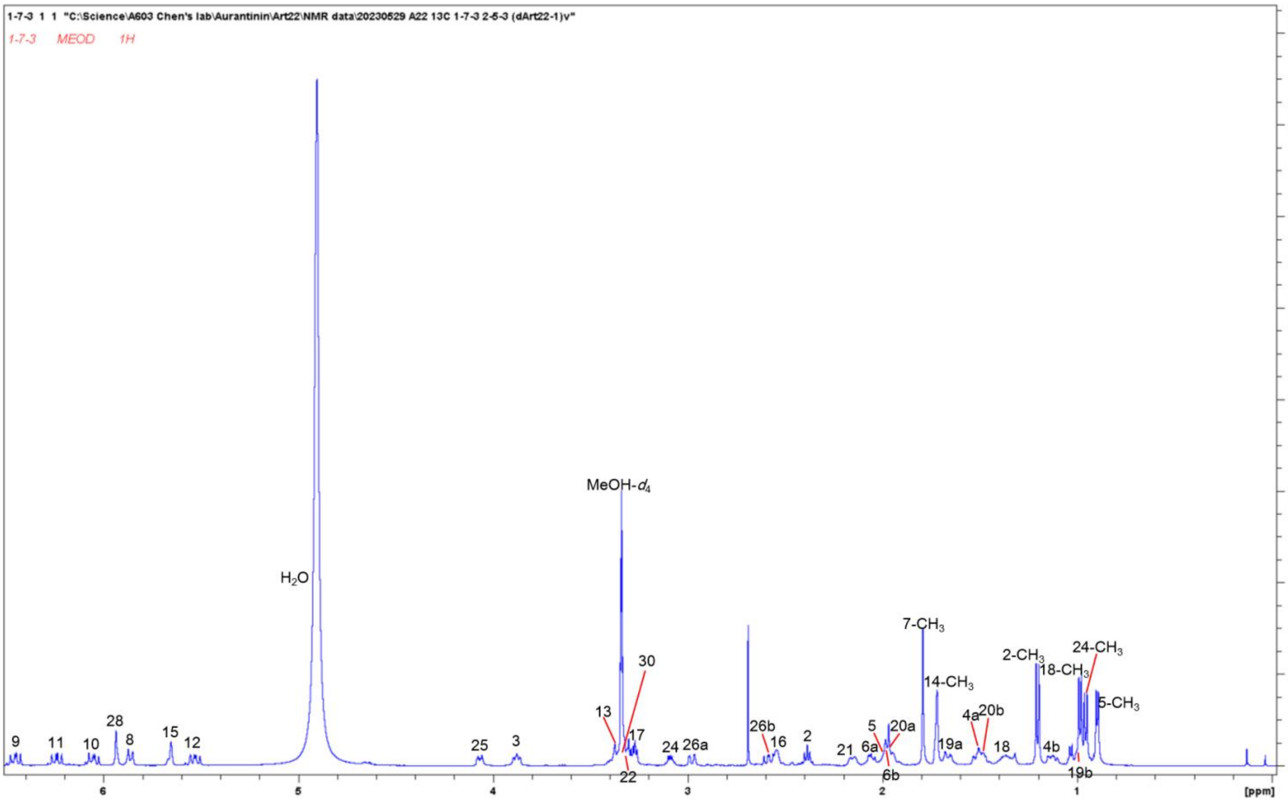

Figure S8. Spectral data of ATA 1 (**5**). (**A**) HR-ESI-MS spectrum of **5**. (**B**) ^{1}H NMR spectra (500 MHz) of **5** in MeOH-d_4.

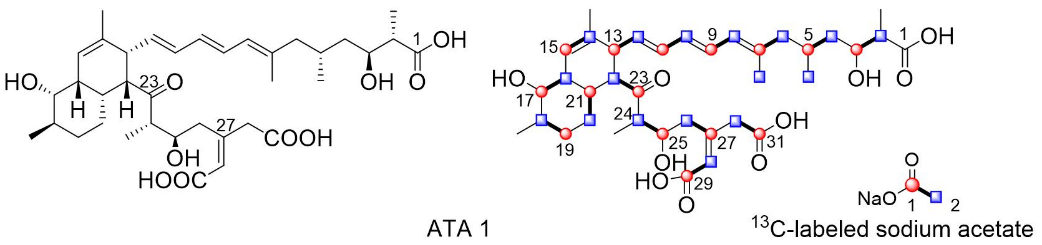

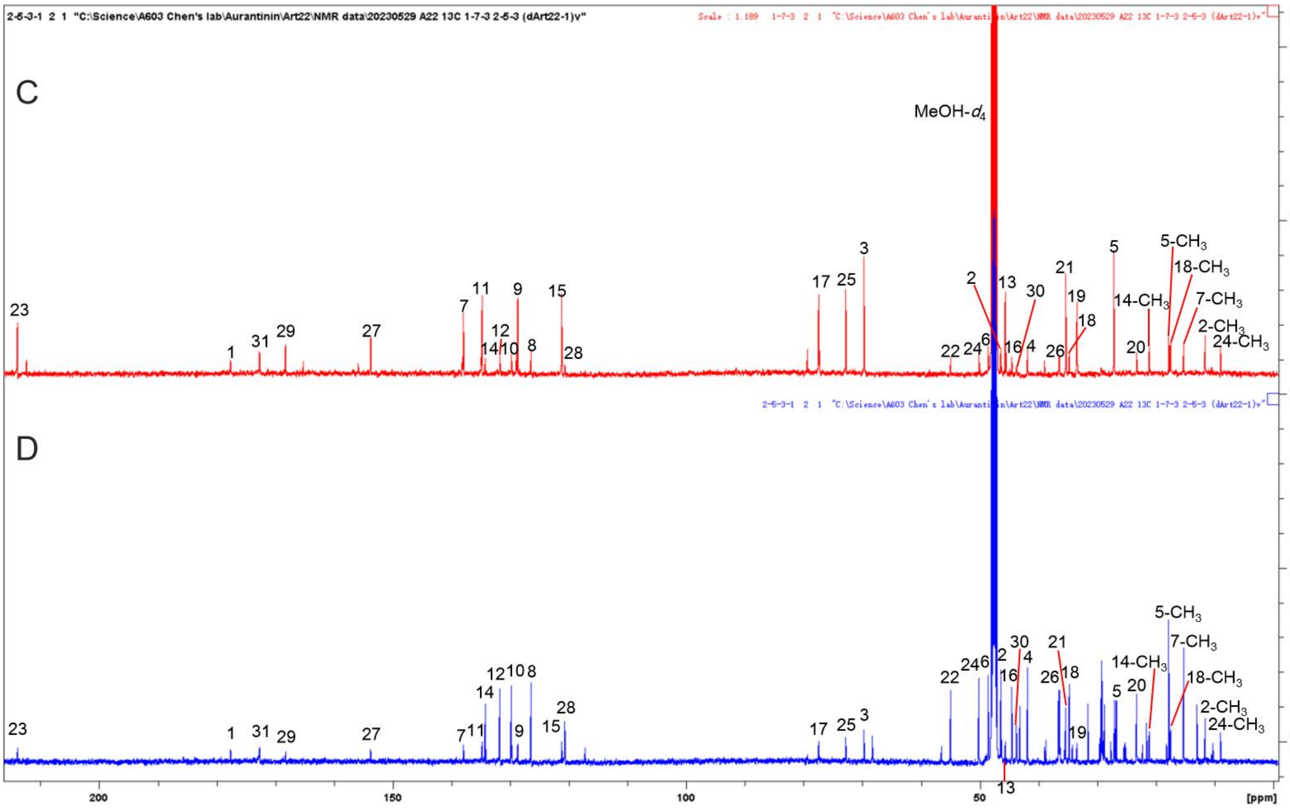

Figure S8. Spectral data of ATA 1 (**5**). (**C**) ^{13}C NMR of **5** labeled by sodium acetate-1-(^{13}C) in MeOH-d_4; (**D**) ^{13}C NMR of **5** labeled by sodium acetate-2-(^{13}C) in MeOH-d_4.

E

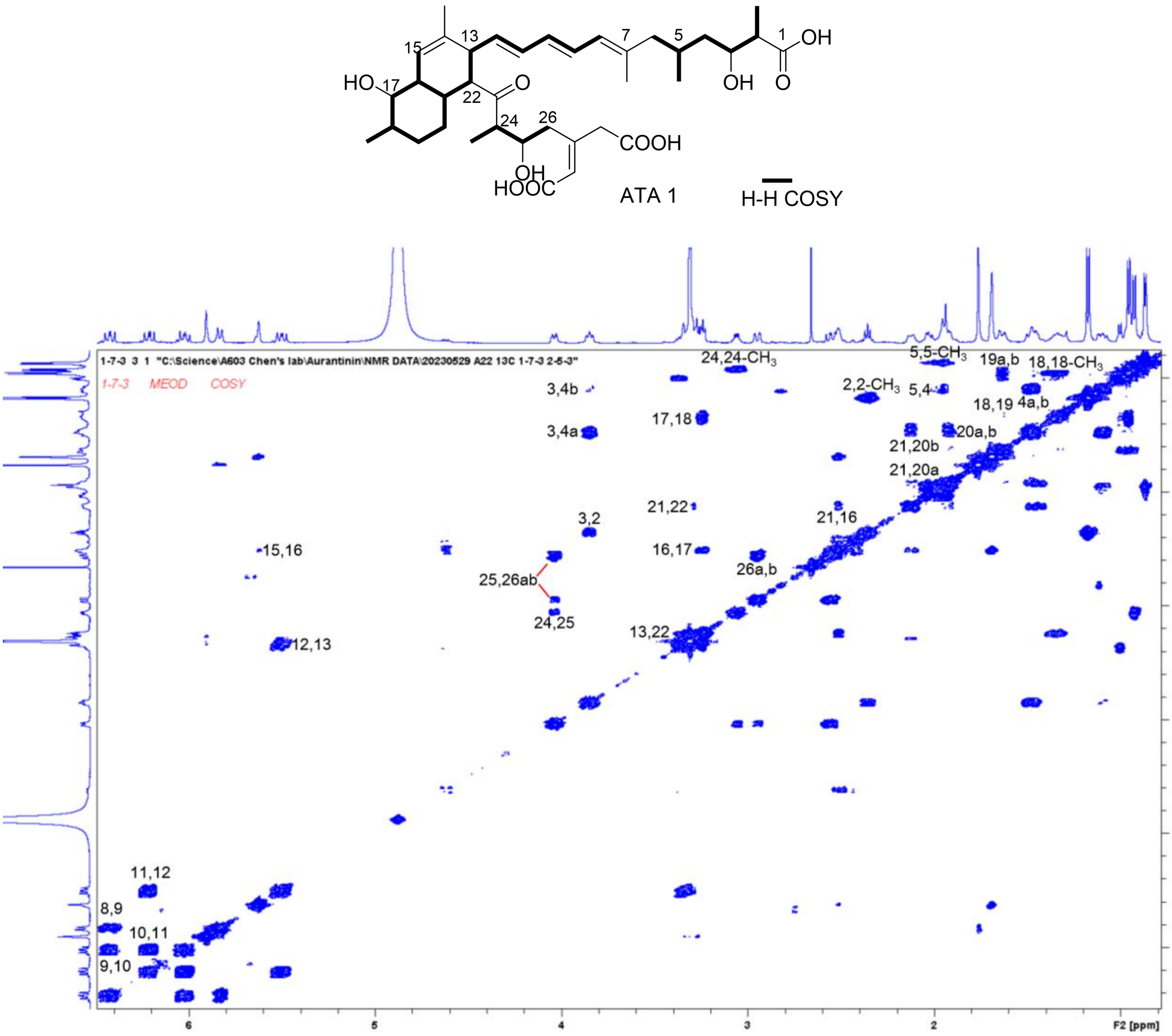

Figure S8. Spectral data of ATA 1 (**5**). (**E**) ^{1}H-^{1}H COSY spectrum (500 MHz) of **5** in MeOH-d_4.

F

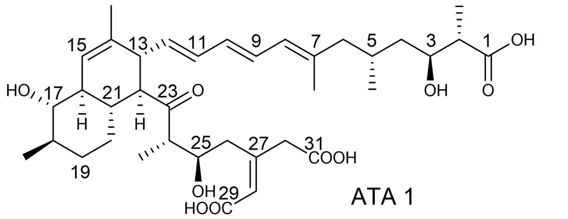

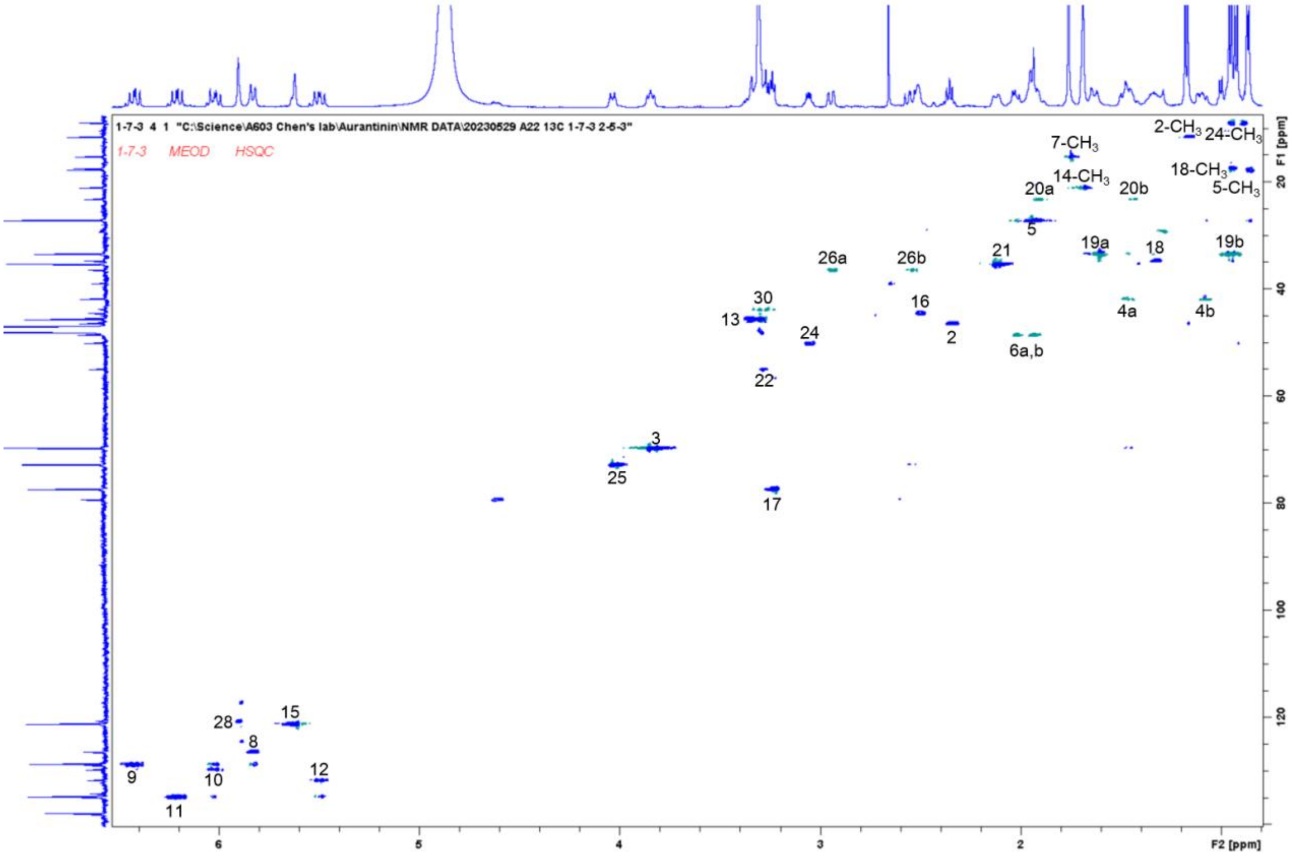

Figure S8. Spectral data of ATA 1 (**5**). (**F**) ^{1}H-^{13}C HSQC spectrum (500 MHz) of **5** in MeOH-d_4.

G

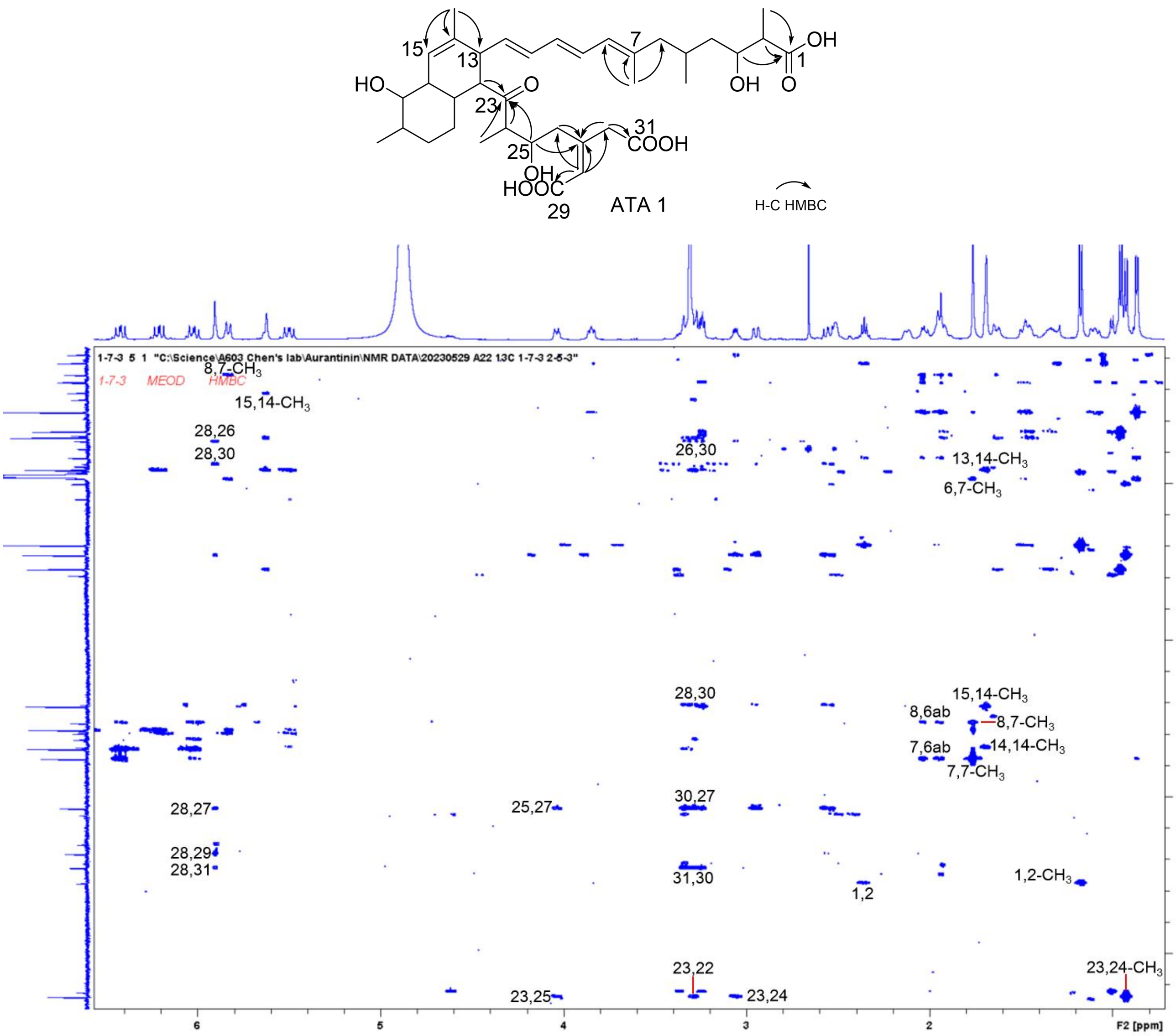

Figure S8. Spectral data of ATA 1 (**5**). (**G**) ^{1}H-^{13}C HMBC spectrum (500 MHz) of **5** in MeOH-d_4.

H

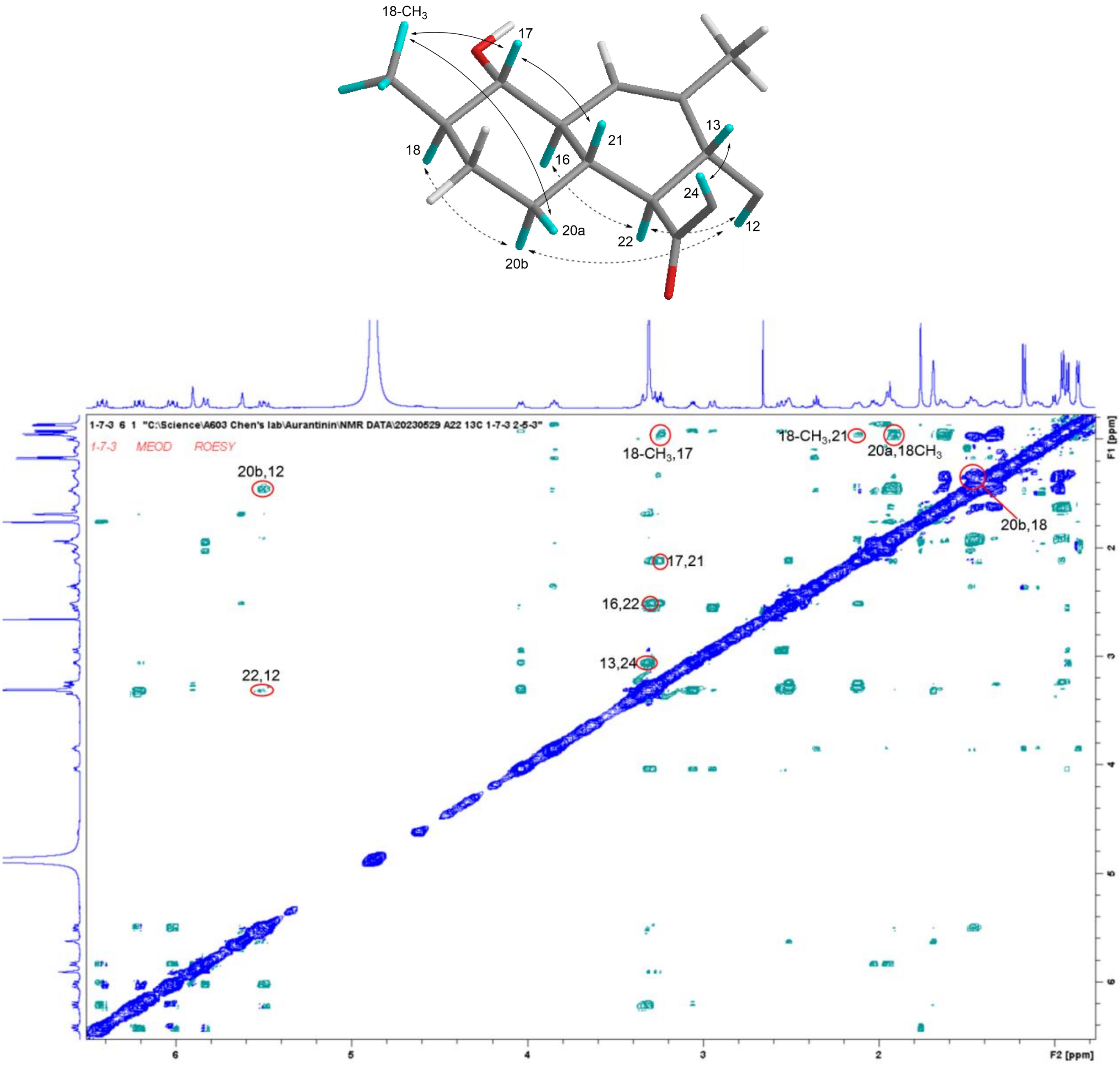

Figure S8. Spectral data of ATA 1 (**5**). (**H**) ^{1}H-^{1}H ROESY spectrum (500 MHz) of **5** in MeOH-d_4.

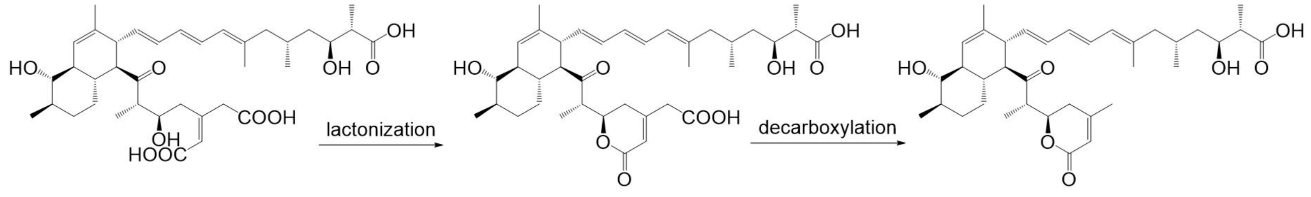

ATA 1 (**5**) ATA 2 (**6**) ATA 3 (**7**)

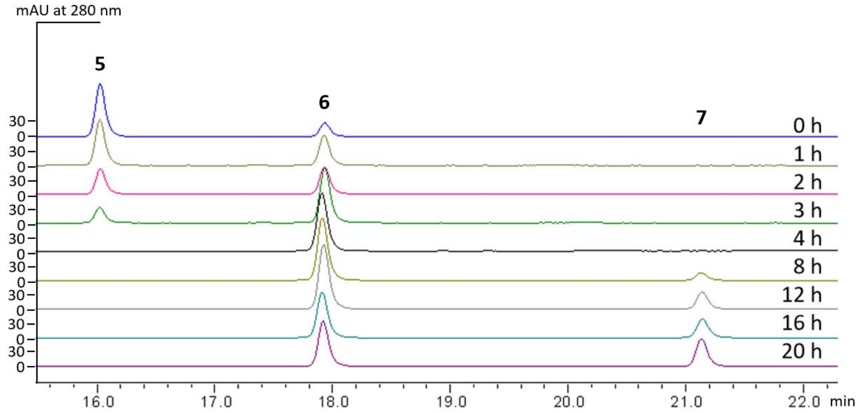

Figure S9. Evaluation of the stability of compound **5**. The stability of **5** was checked by time-course analysis in a solution containing 1:1 of H_2O and acetonitrile with 0.5‰ of formic acid. **5** underwent a spontaneous lactonization to generate **6**, followed by an auto-decarboxylation to form **7**. HPLC analysis was carried out with an Apollo C18 column (5 μm, 4.6 mm × 250 mm, Alltech, IL, USA) on a Shimadzu HPLC system (Shimadzu, Kyoto, Japan). The detection wavelength was 280 nm. The column was developed with acetonitrile and water containing 0.1% formic acid at a flow rate of 1 mL/min. Percentage of acetonitrile was changed from 40% to 65% at 0-30 min.

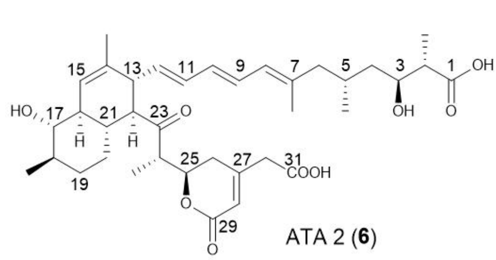

A

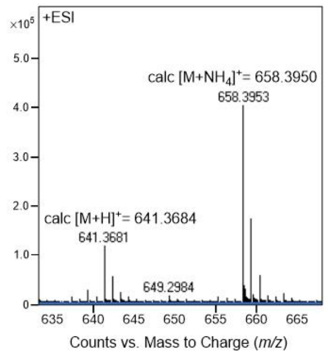

B

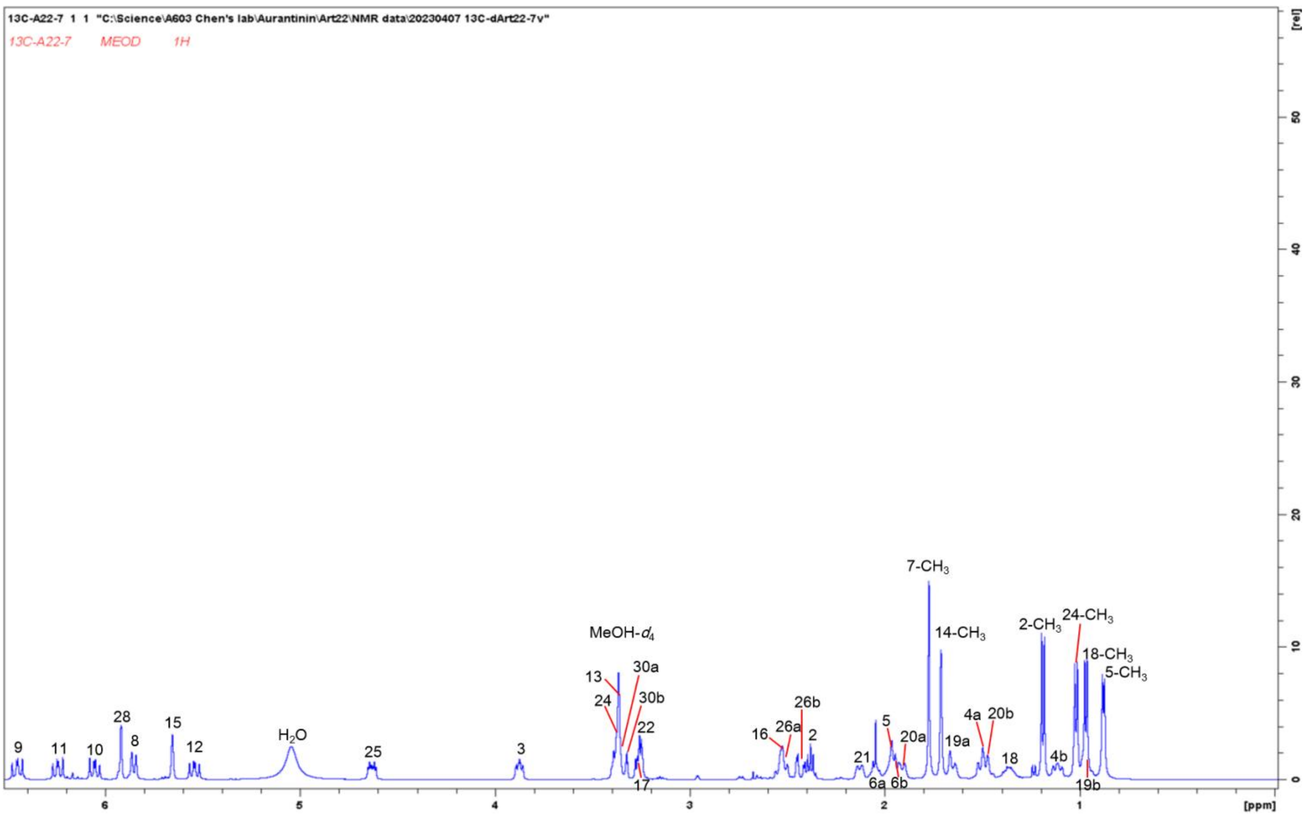

Figure S10. Spectral data of ATA 2 (**6**). (**A**) HR-ESI-MS spectrum of **6**. (**B**) ^{1}H NMR spectra (500 MHz) of **6** in MeOH-d_4.

C

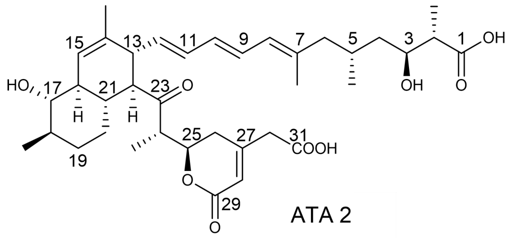

ATA 2

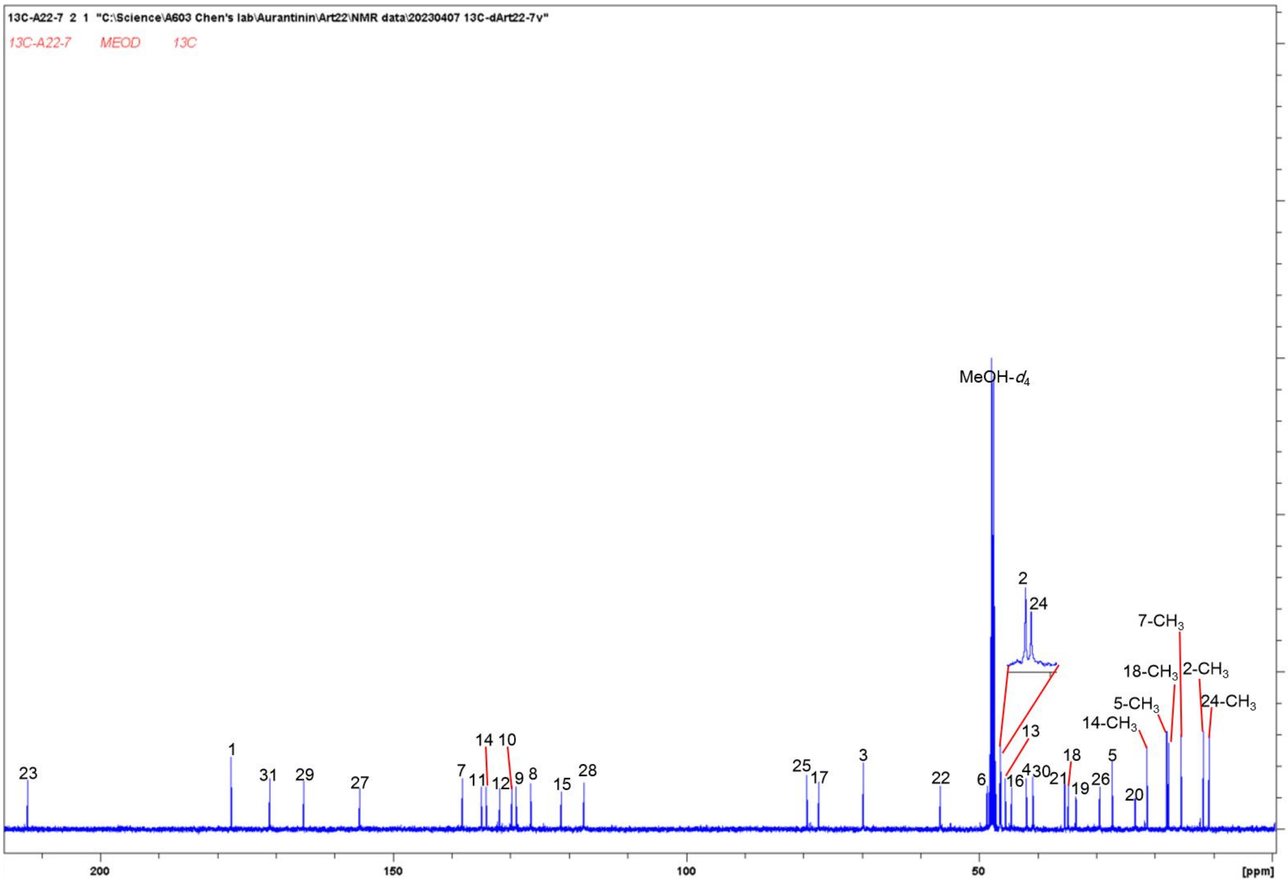

Figure S10. Spectral data of ATA 2 (**6**). (**C**) ^{13}C NMR spectrum (125 MHz) of **6** in MeOH-d_4.

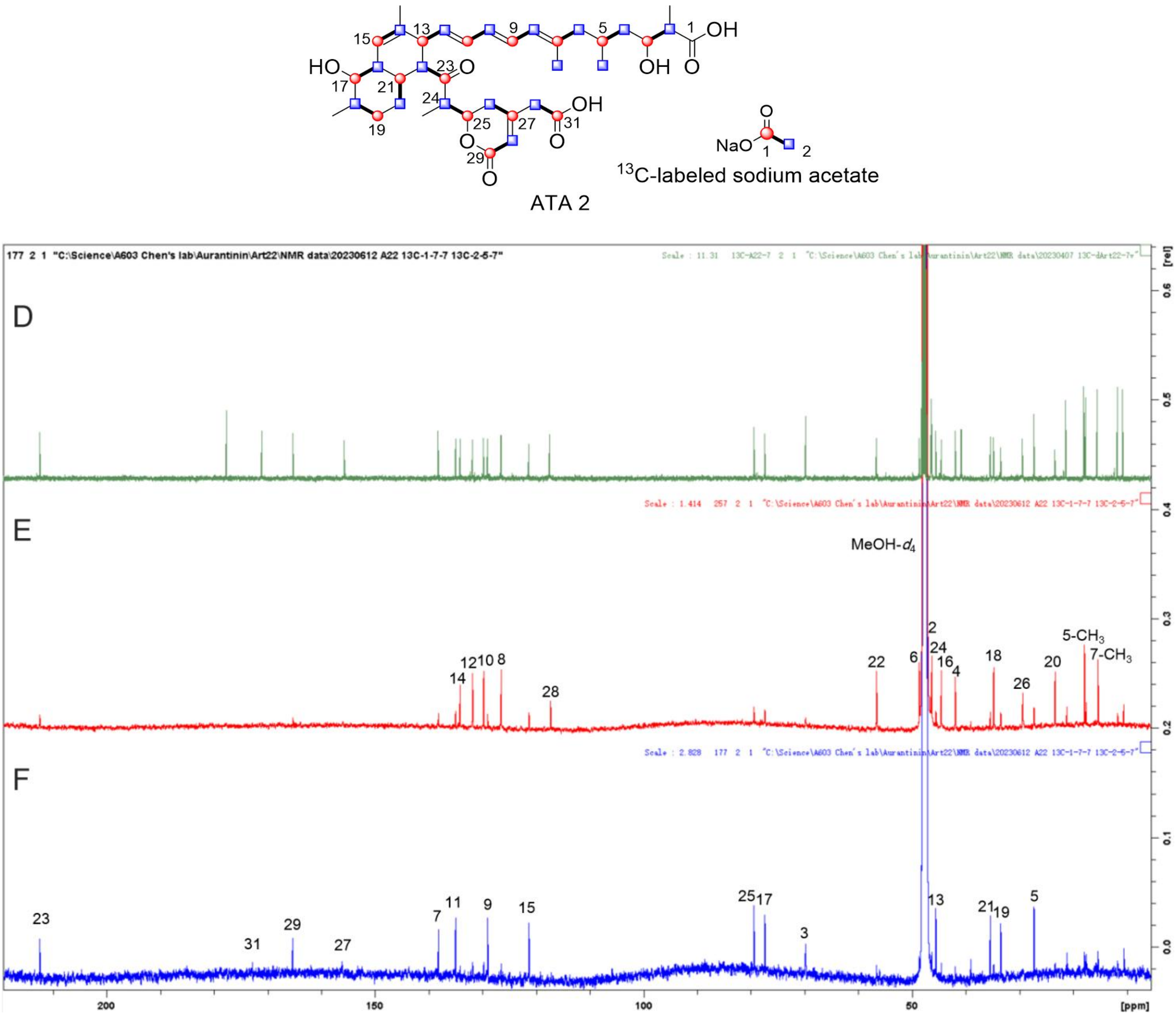

Figure S10. Spectral data of ATA 2 (**6**). (**D**) ^{13}C NMR spectrum (125 MHz) of **6** in MeOH-*d*4; (**E**) ^{13}C NMR of **6** labeled by sodium acetate-1-(^{13}C); (**F**) ^{13}C NMR of **6** labeled by sodium acetate-2-(^{13}C).

G

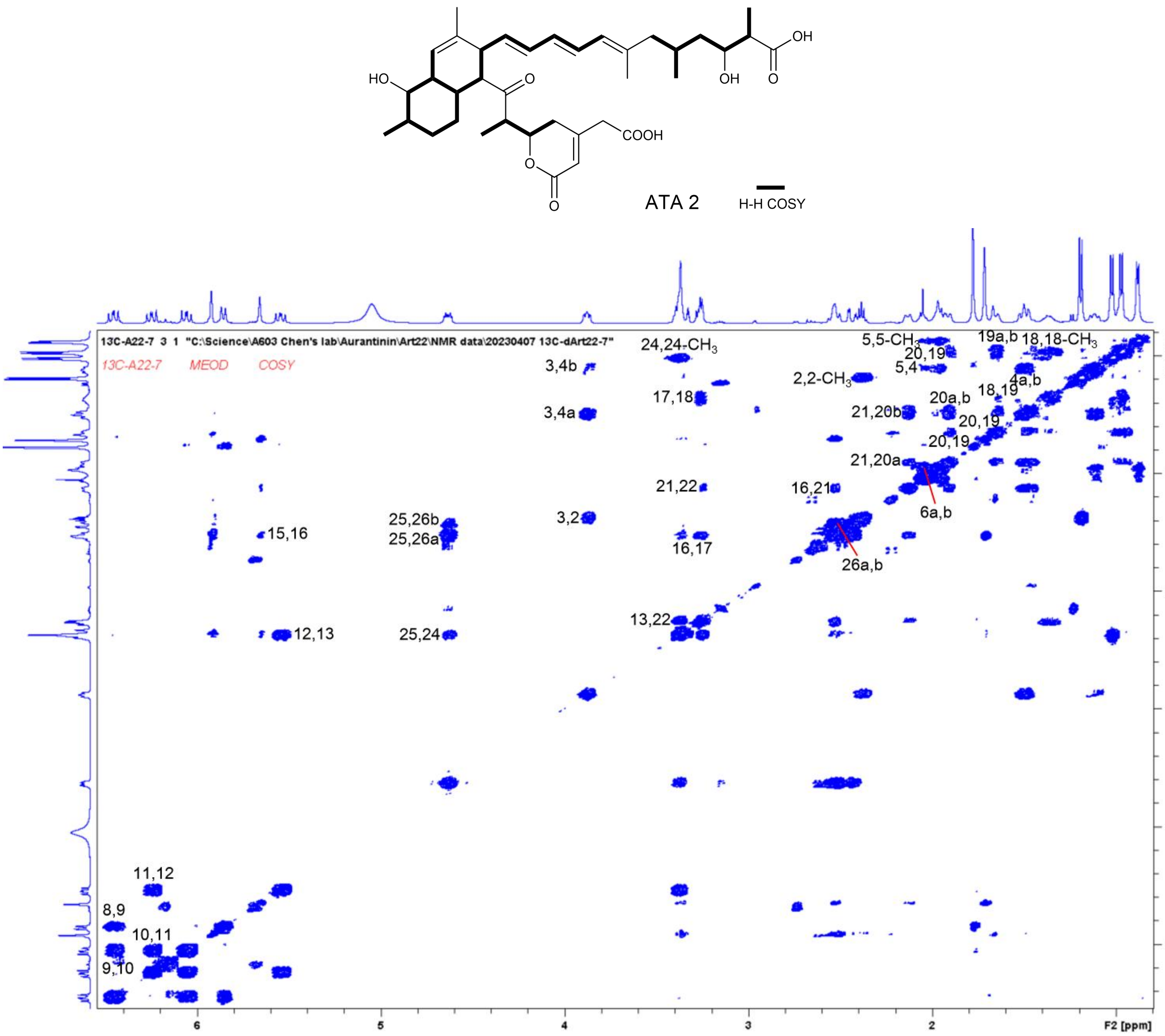

Figure S10. Spectral data of ATA 2 (**6**). (**G**) ^{1}H-^{1}H COSY spectrum (500 MHz) of **6** in MeOH-d_4.

H

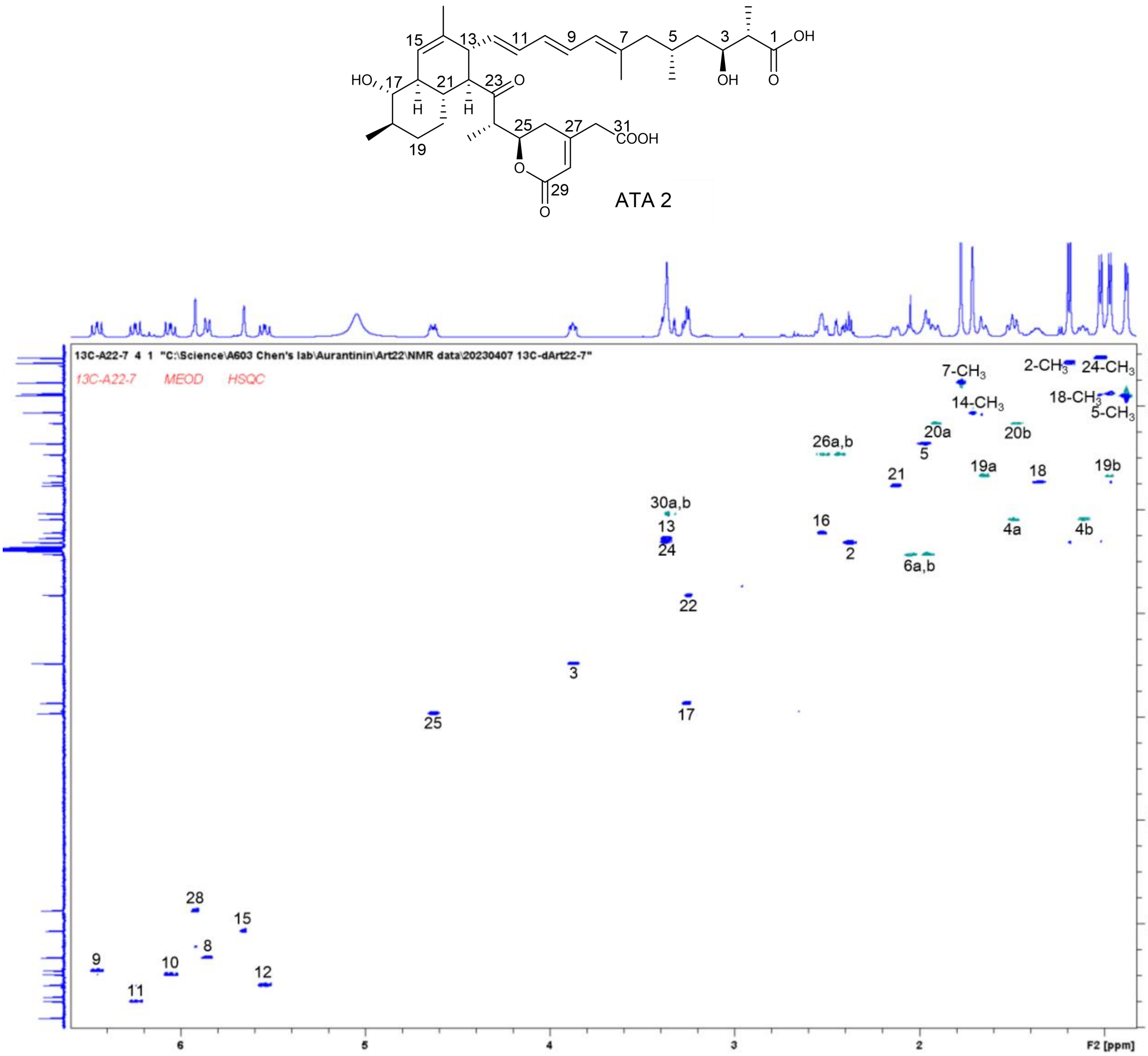

Figure S10. Spectral data of ATA 2 (**6**). (**H**) ^{1}H-^{13}C HSQC spectrum (500 MHz) of **6** in MeOH-*d*$_4$.

I

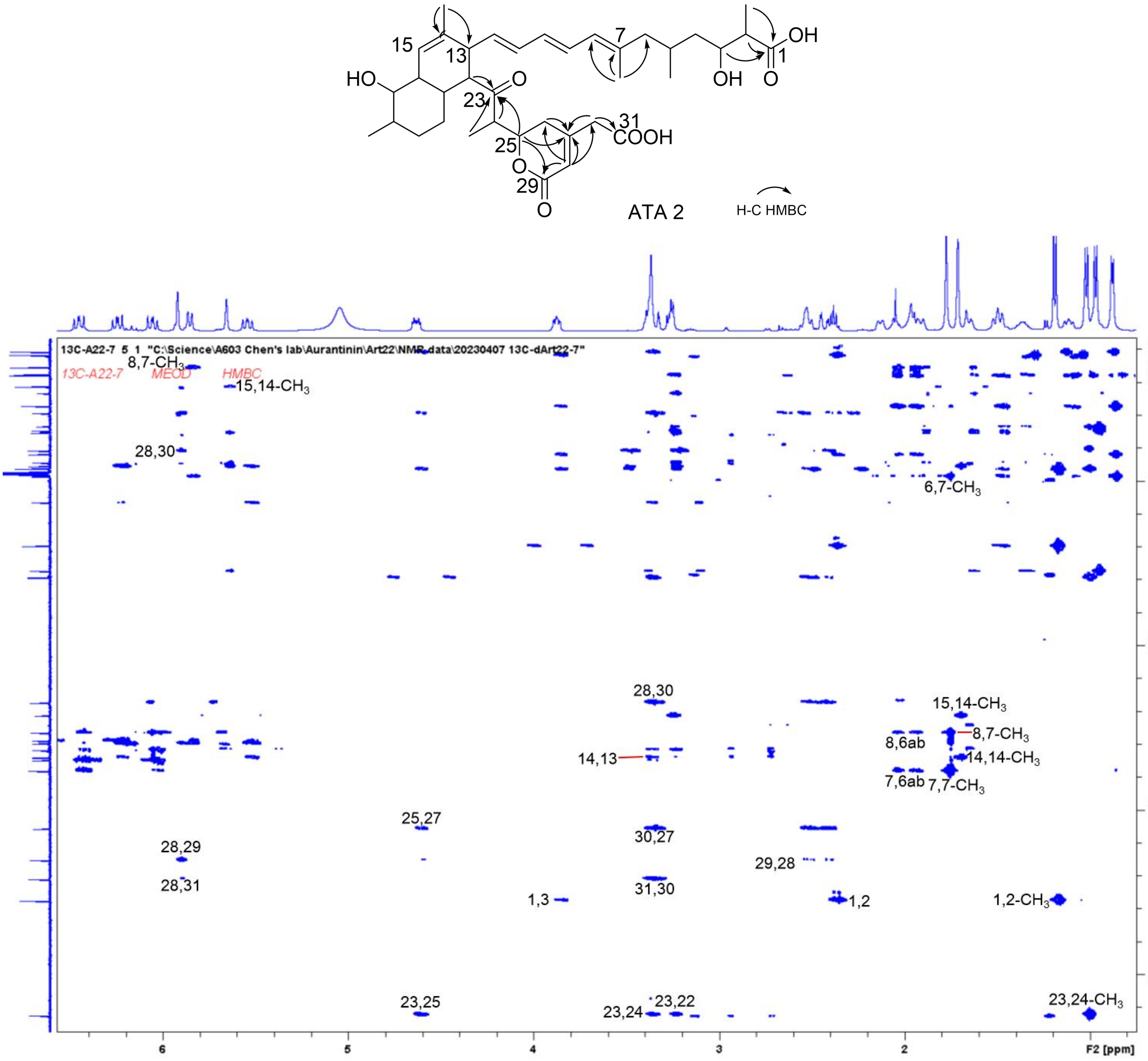

Figure S10. Spectral data of ATA 2 (**6**). (**I**) ^{1}H-^{13}C HMBC spectrum (500 MHz) of **6** in MeOH-d_4.

J

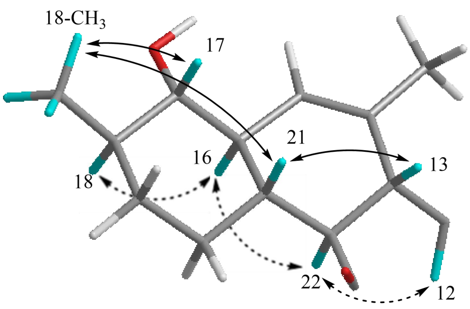

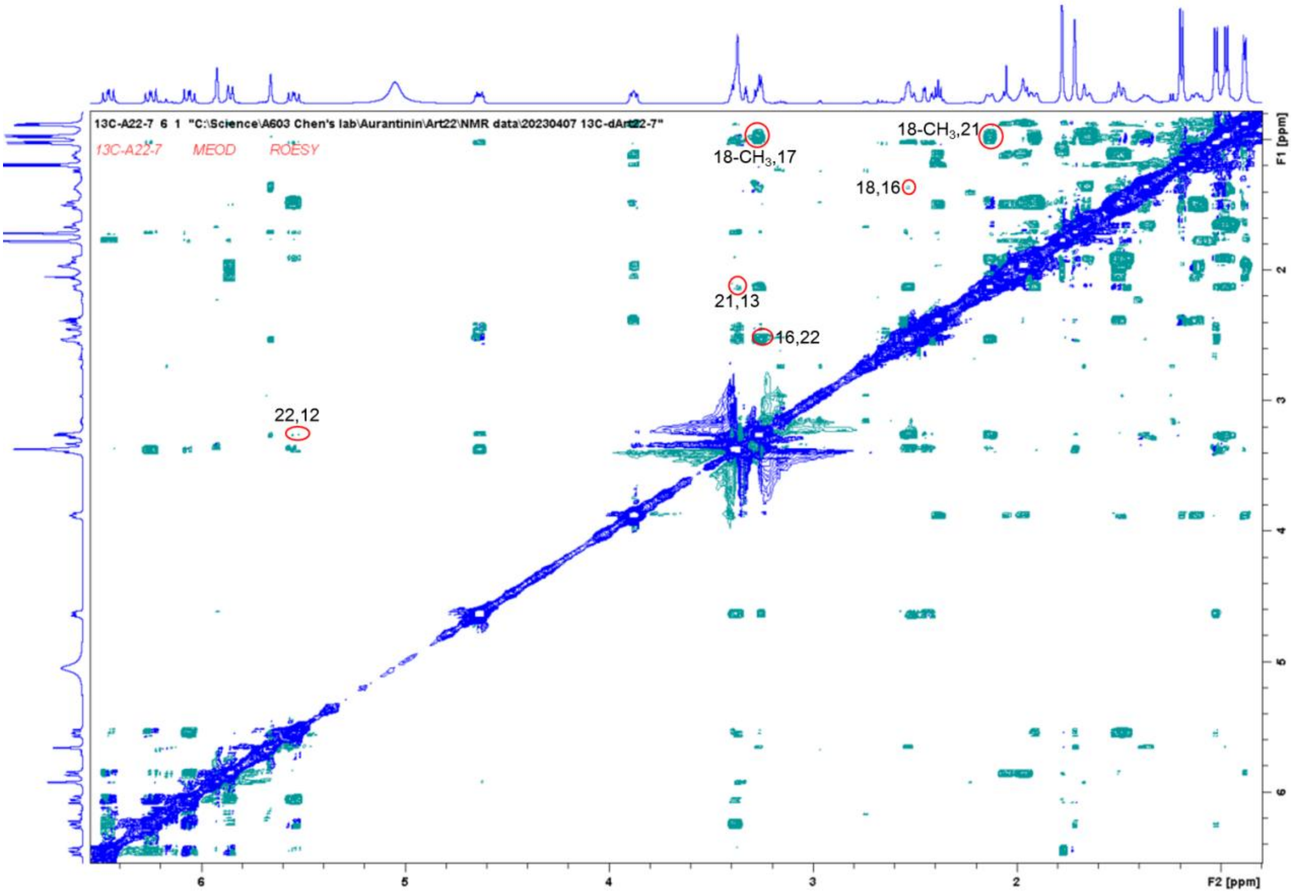

Figure S10. Spectral data of ATA 2 (**6**). (**J**) ^{1}H-^{1}H ROESY spectrum (500 MHz) of **6** in MeOH-d_4.

A

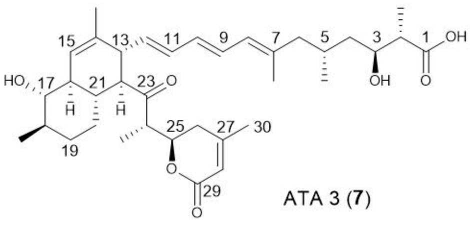

ATA 3 (**7**)

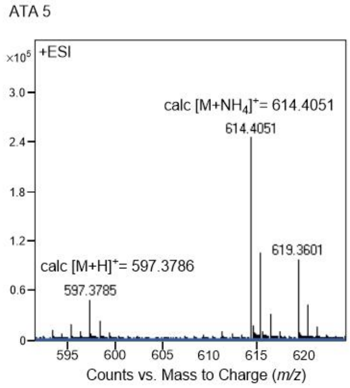

B

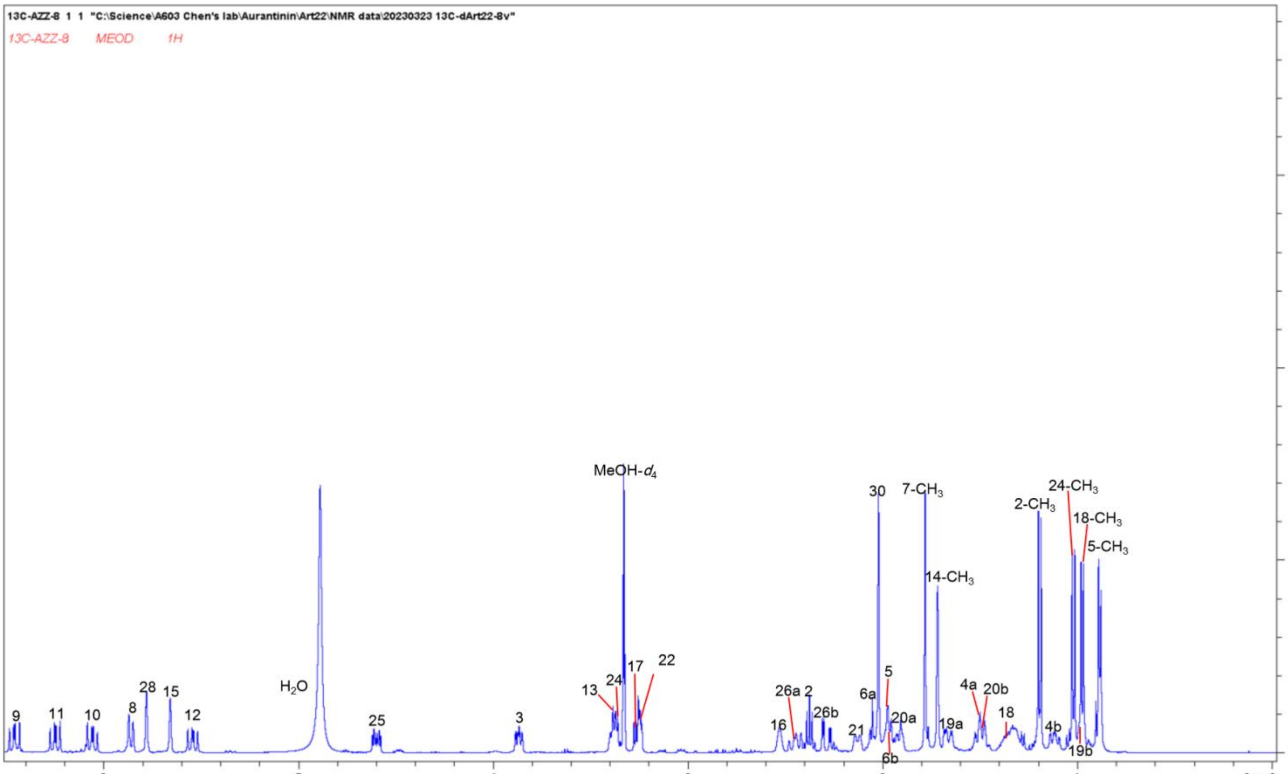

Figure S11. Spectral data of ATA 3 (**7**). (**A**) HR-ESI-MS spectrum of **7**; (**B**) ^{1}H NMR spectra (500 MHz) of **7** in MeOH-d_4.

C

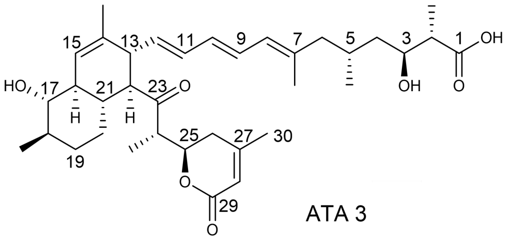

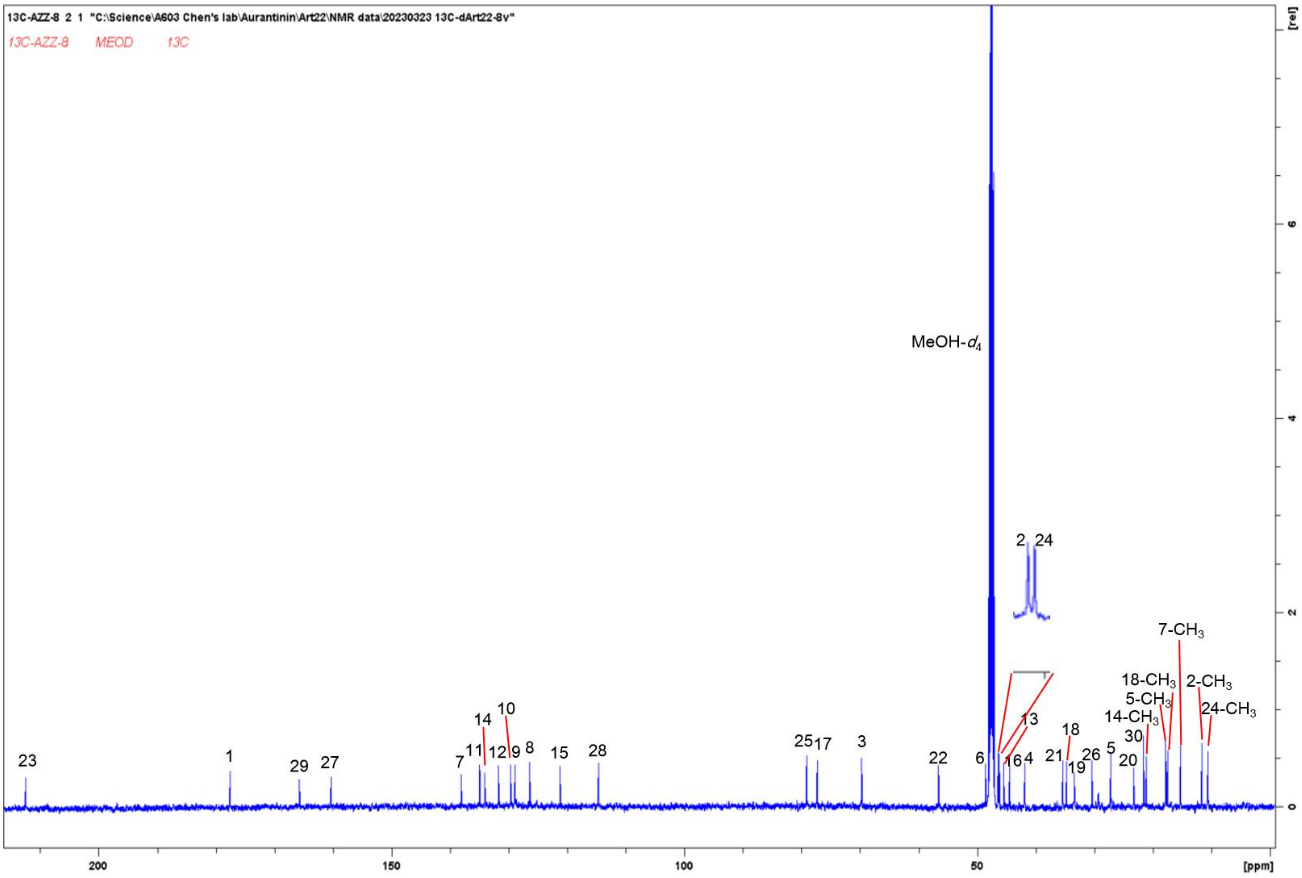

Figure S11. Spectral data of ATA 3 (**7**). (**C**) ^{13}C NMR spectrum (125 MHz) of **7** in MeOH-d_4.

D

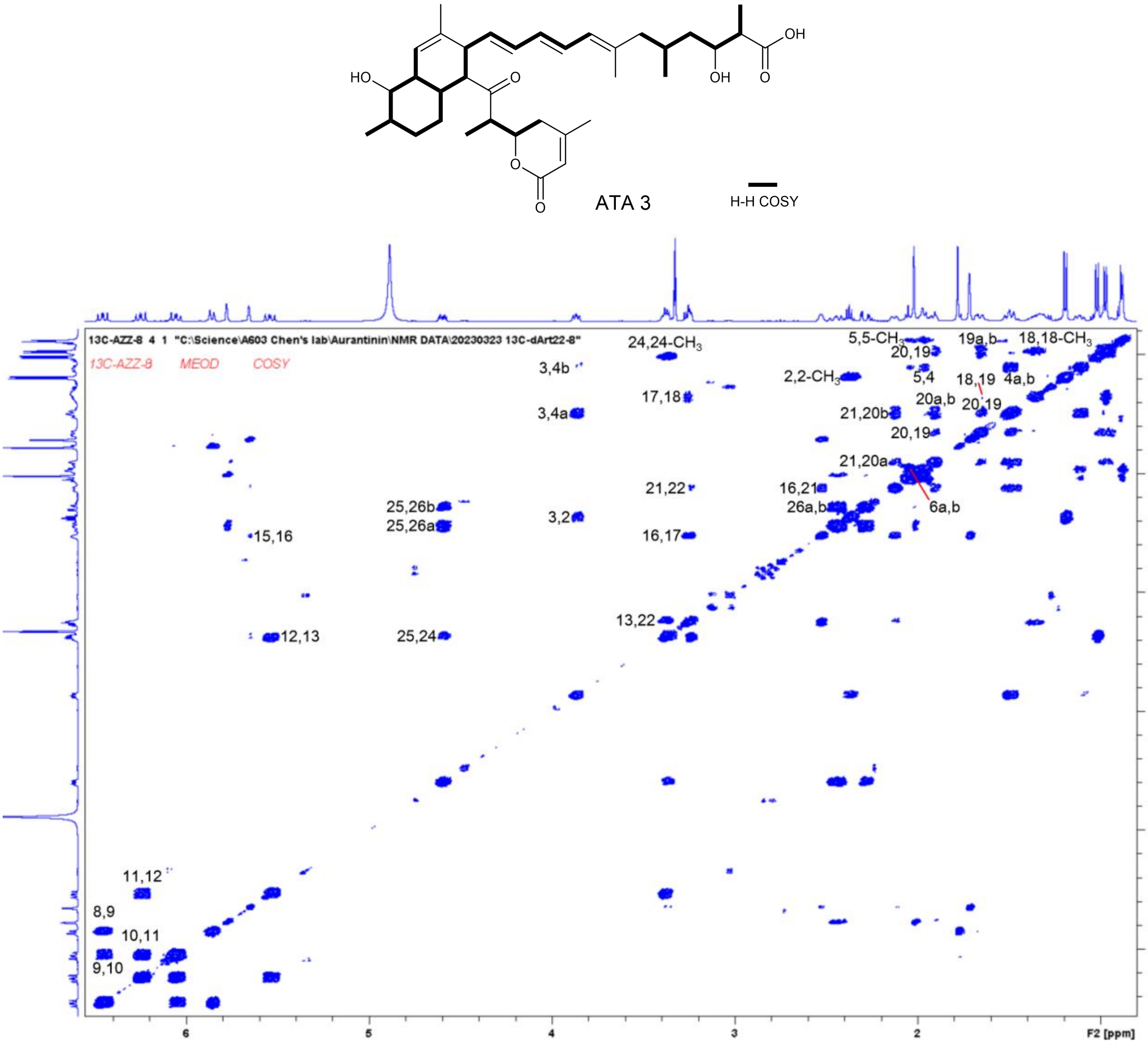

Figure S11. Spectral data of ATA 3 (**7**). (**D**) ^{1}H-^{1}H COSY spectrum (500 MHz) of **7** in MeOH-d_4.

E

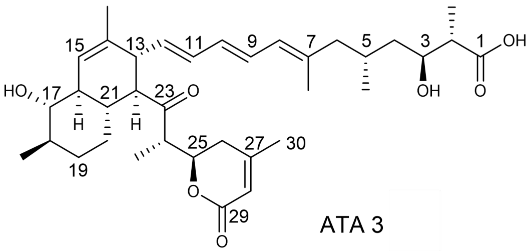

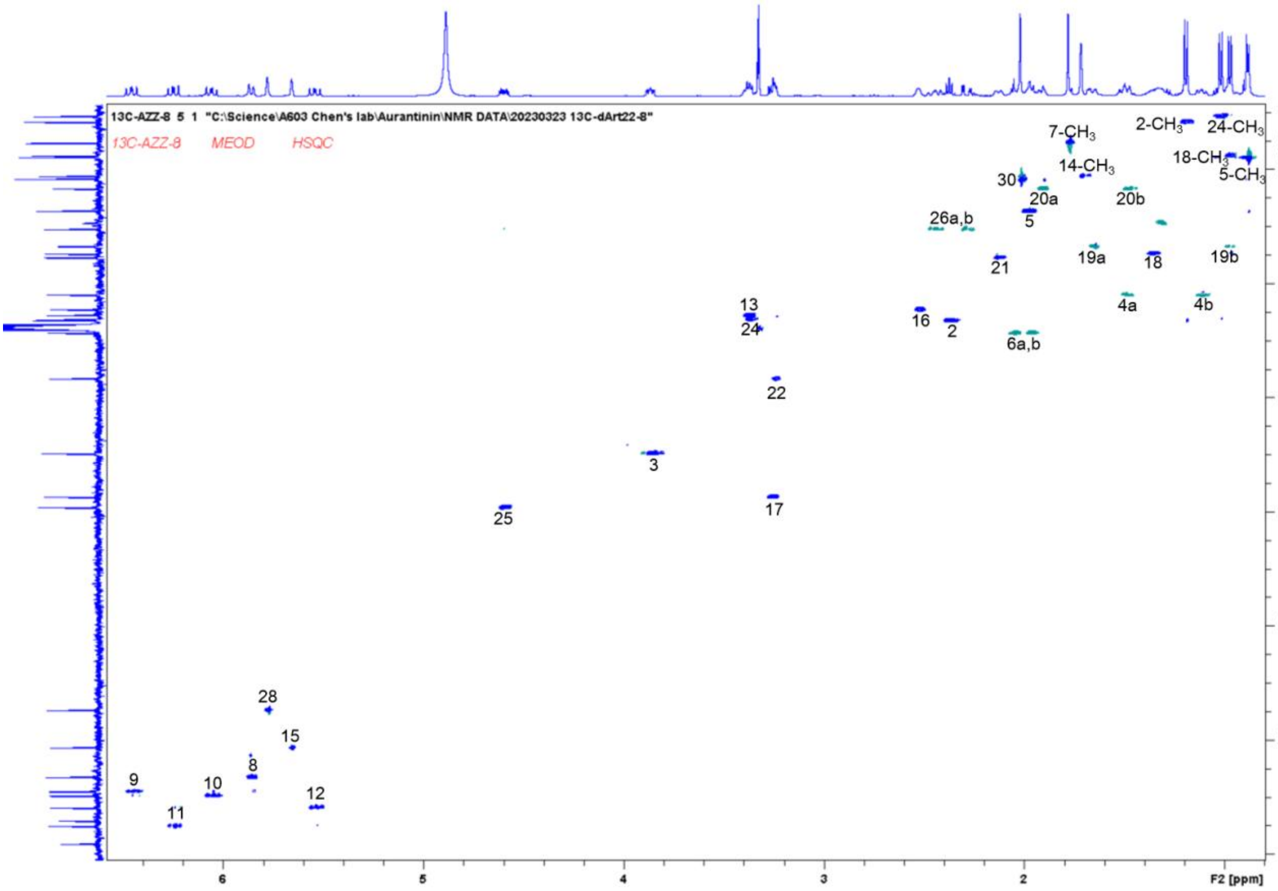

Figure S11. Spectral data of ATA 3 (**7**). (**E**) ^{1}H-^{13}C HSQC spectrum (500 MHz) of **7** in MeOH-d_4.

F

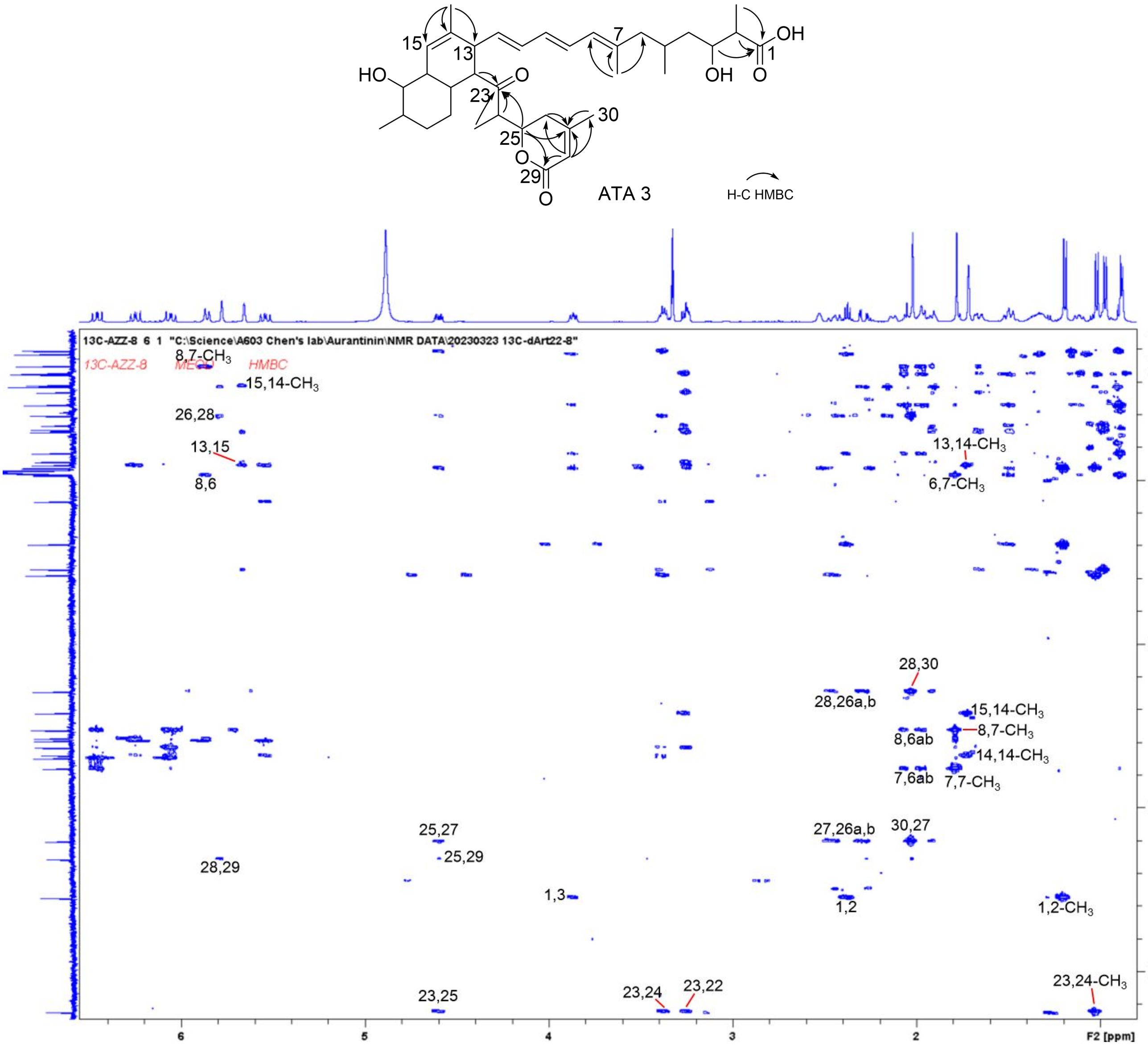

Figure S11. Spectral data of ATA 3 (**7**). (**F**) ^{1}H-^{13}C HMBC spectrum (500 MHz) of **7** in MeOH-d_4.

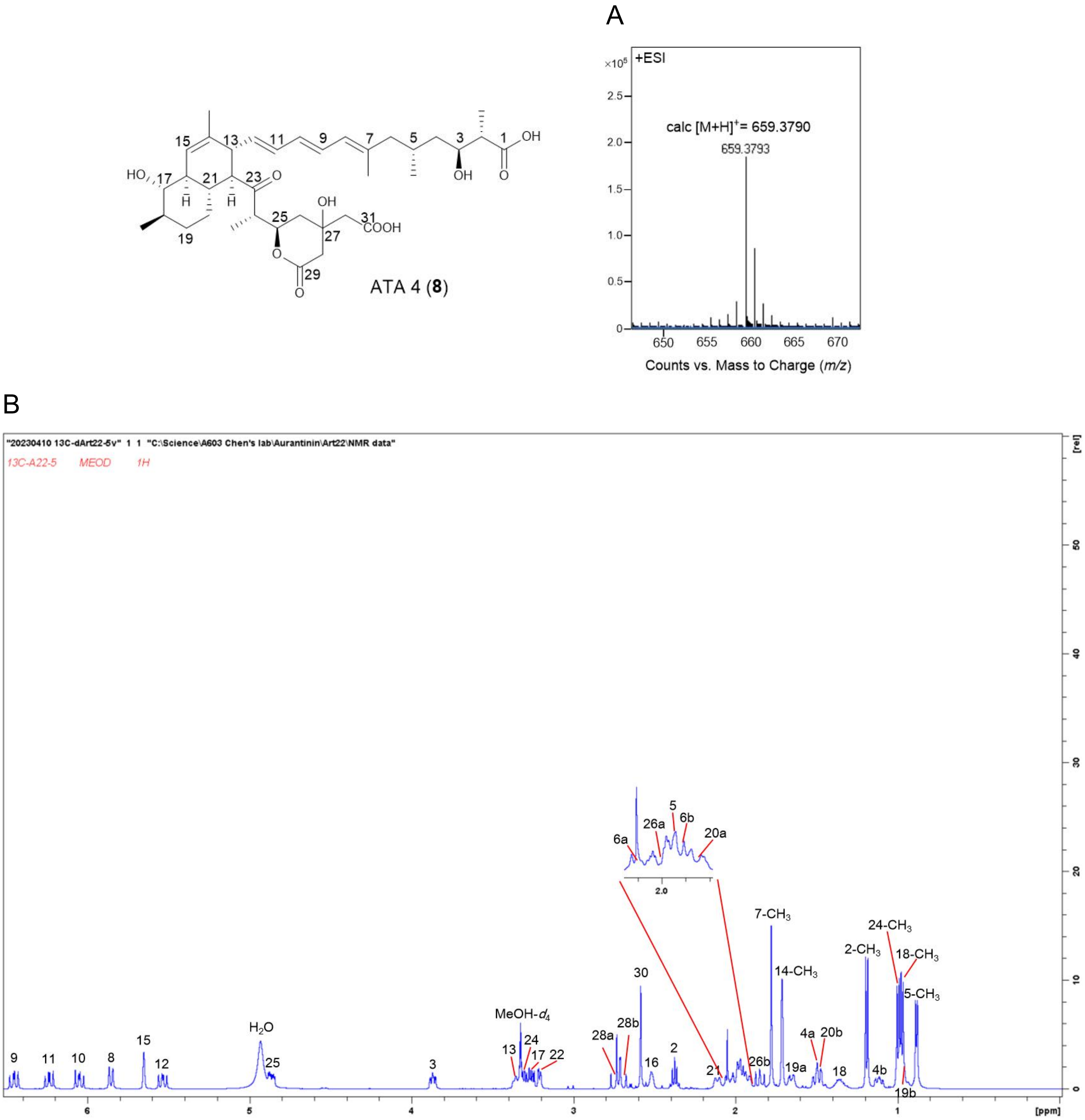

Figure S12. Spectral data of ATA 4 (**8**). (**A**) HR-ESI-MS spectrum of **8**; (**B**) ^{1}H NMR spectra (500 MHz) of **8** in MeOH-d_4.

C

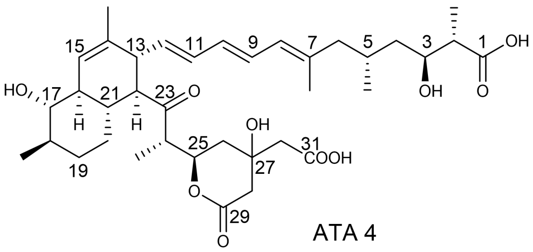

ATA 4

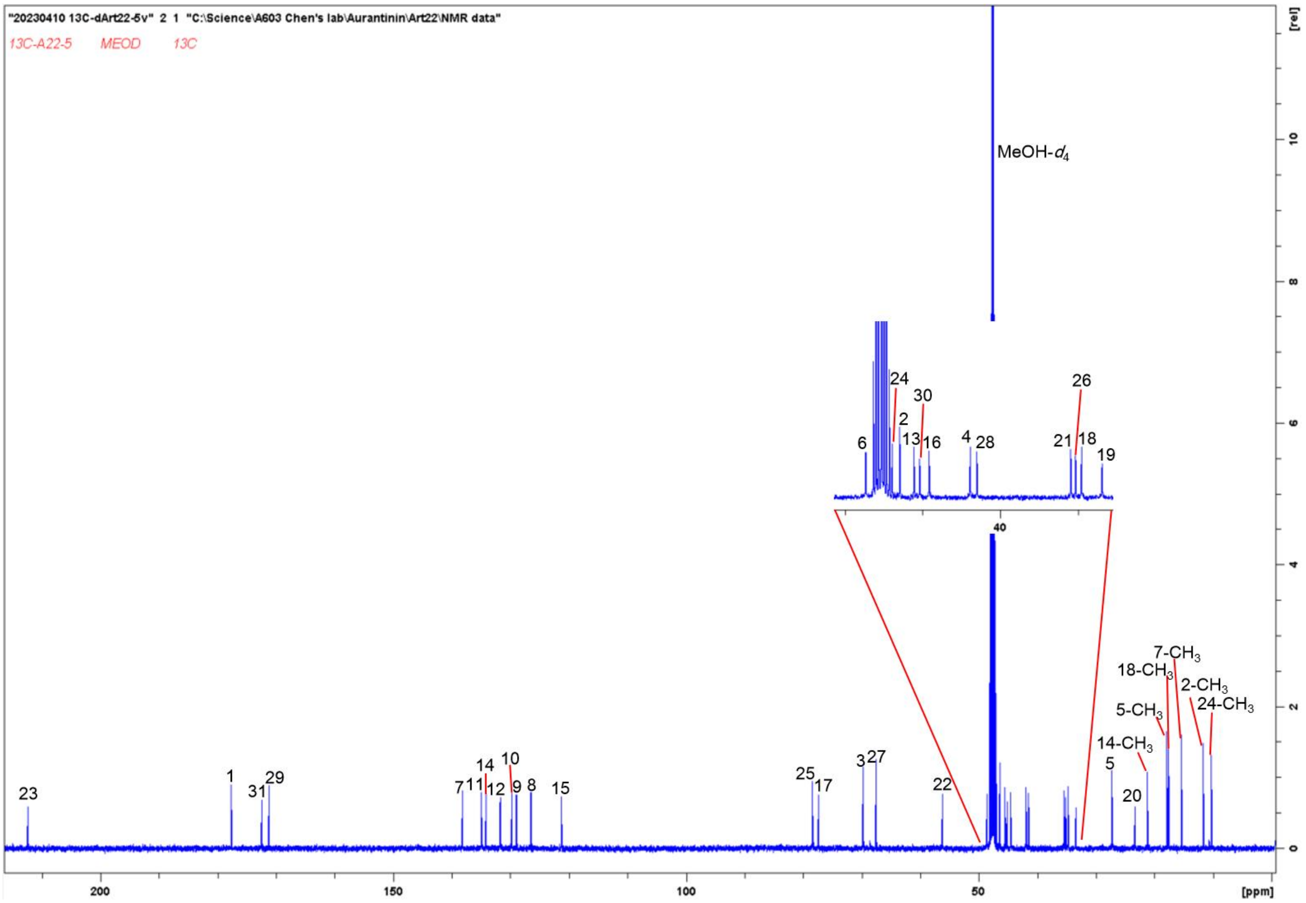

Figure S12. Spectral data of ATA 4 (**8**). (**C**) ^{13}C NMR spectrum (125 MHz) of **8** in MeOH-d_4.

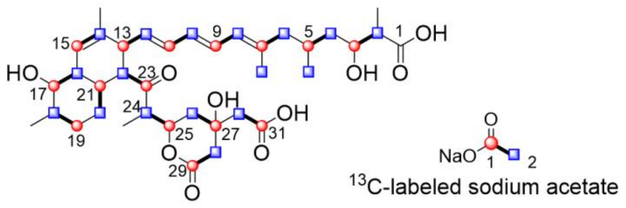

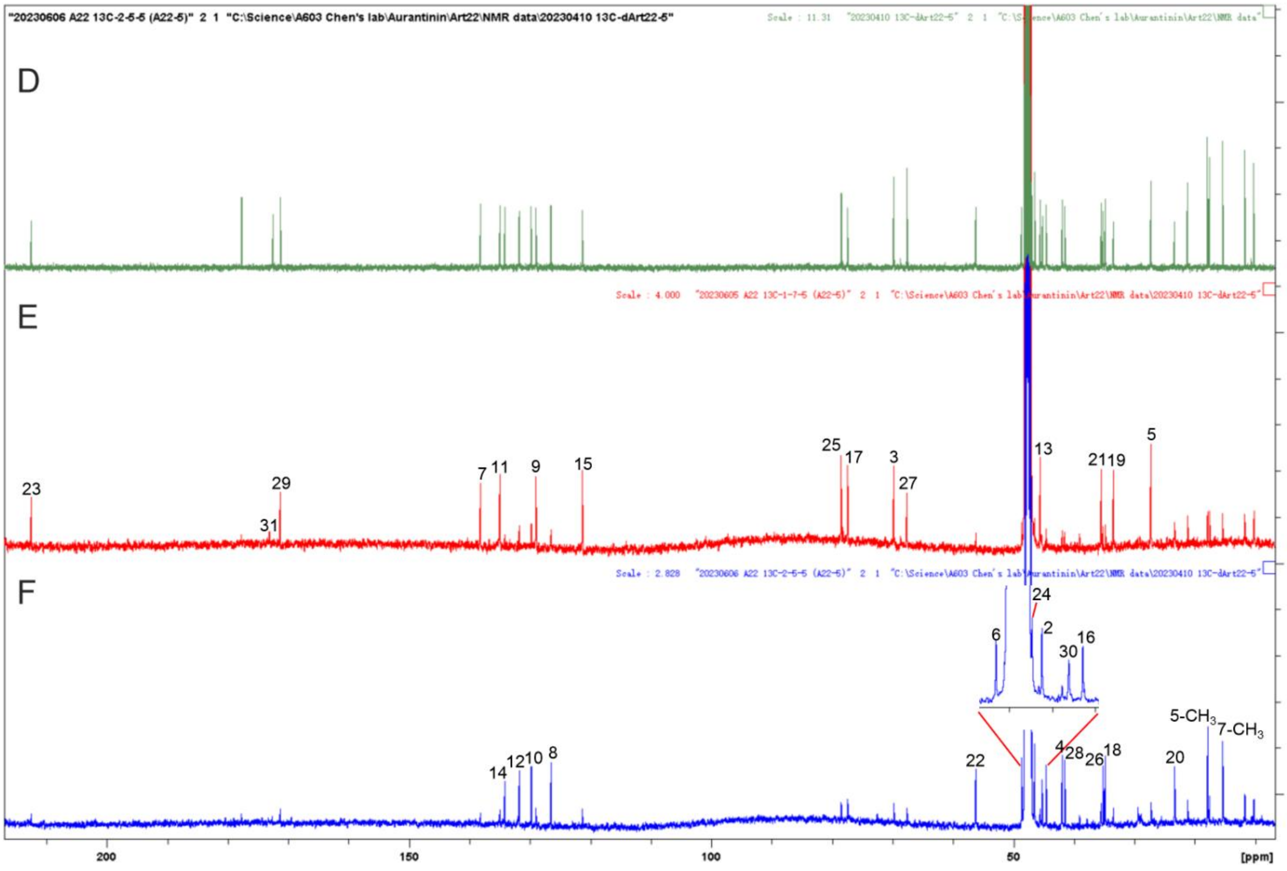

Figure S12. Spectral data of ATA 4 (**8**). (**D**) ^{13}C NMR spectrum (125 MHz) of **8** in MeOH-*d*4; (**E**) ^{13}C NMR of **8** labeled by sodium acetate-1-(^{13}C); (**F**) ^{13}C NMR of **8** labeled by sodium acetate-2-(^{13}C).

G

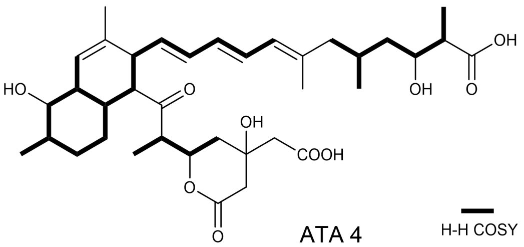

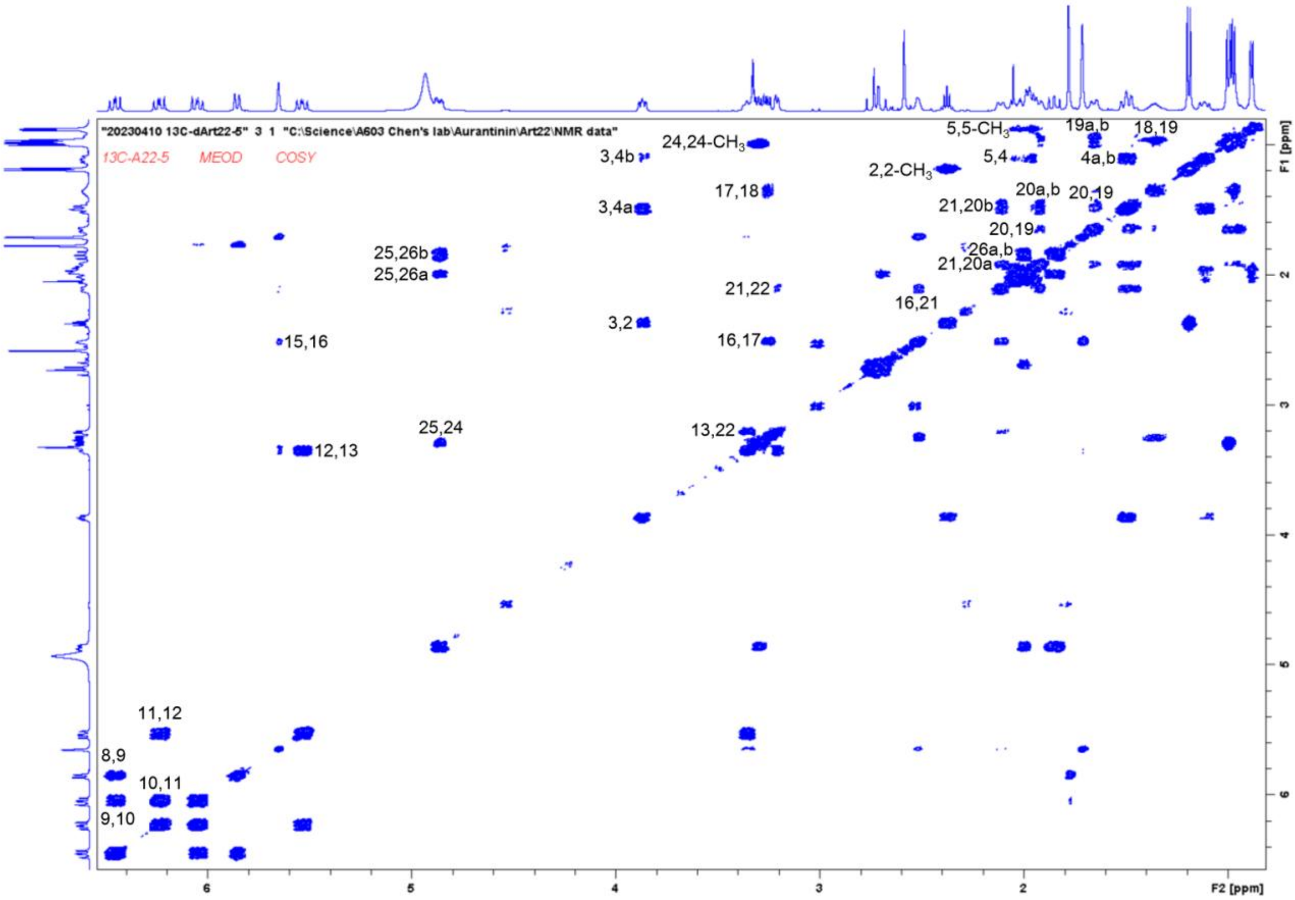

Figure S12. Spectral data of ATA 4 (**8**). (**G**) ^{1}H-^{1}H COSY spectrum (500 MHz) of **8** in MeOH-*d*$_4$.

H

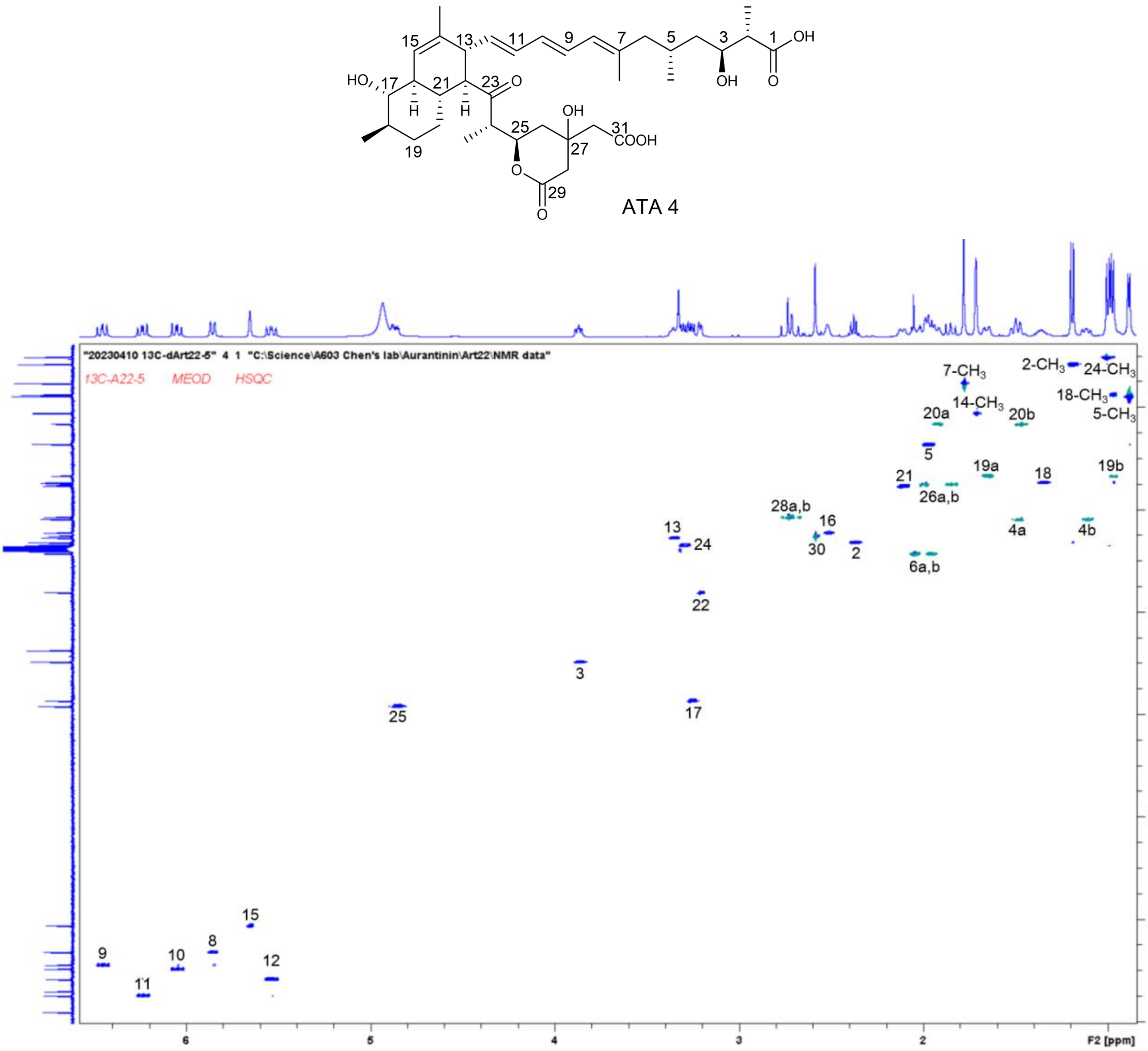

Figure S12. Spectral data of ATA 4 (**8**). (**H**) ^{1}H-^{13}C HSQC spectrum (500 MHz) of **8** in MeOH-d_4.

I

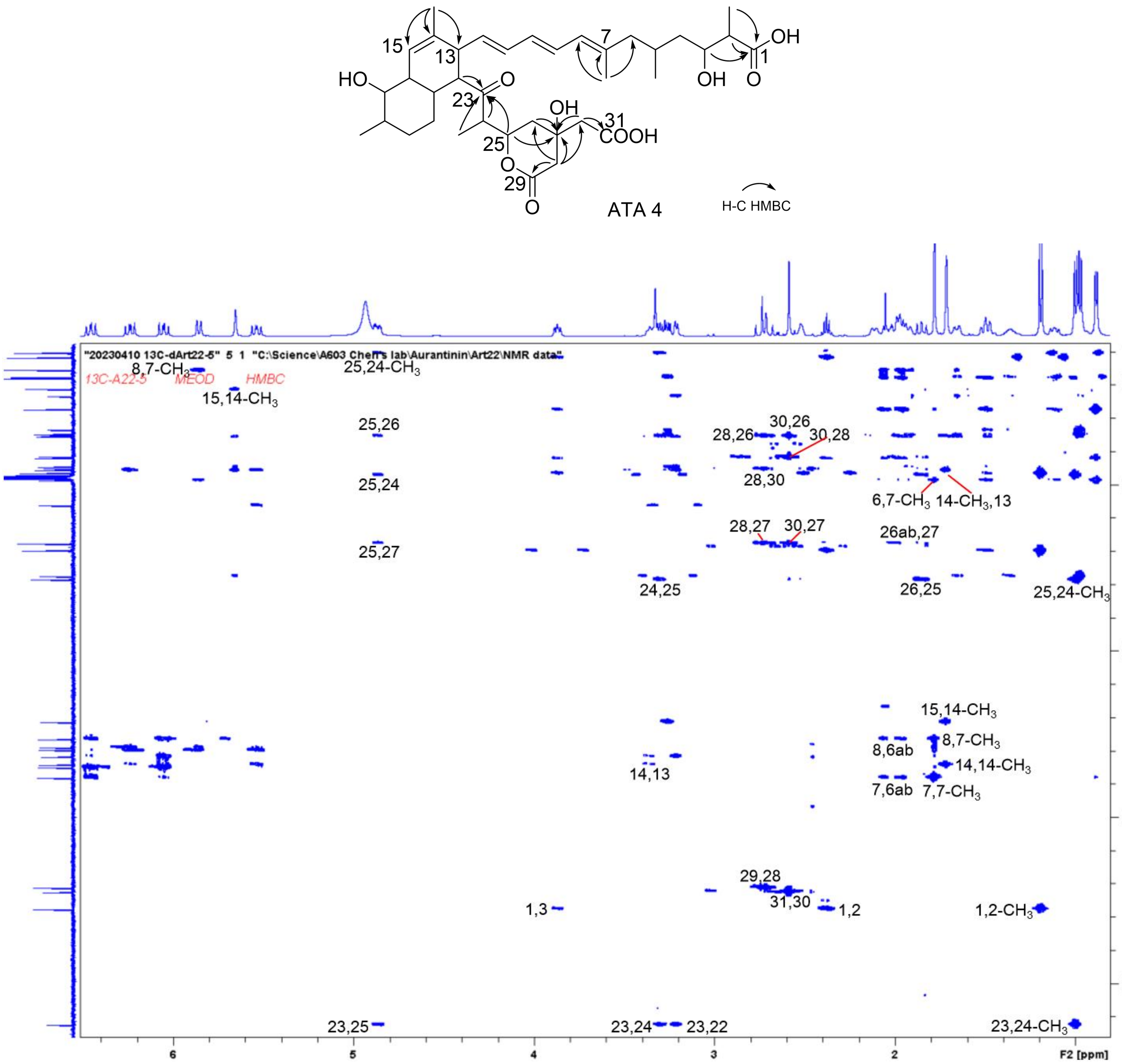

Figure S12. Spectral data of ATA 4 (**8**). (**I**) ^{1}H-^{13}C HMBC spectrum (500 MHz) of **8** in MeOH-d_4.

J

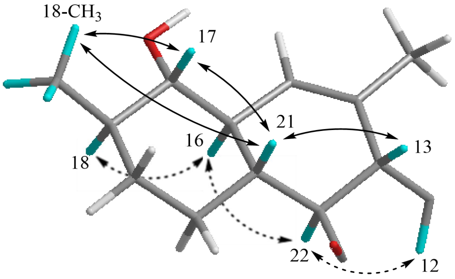

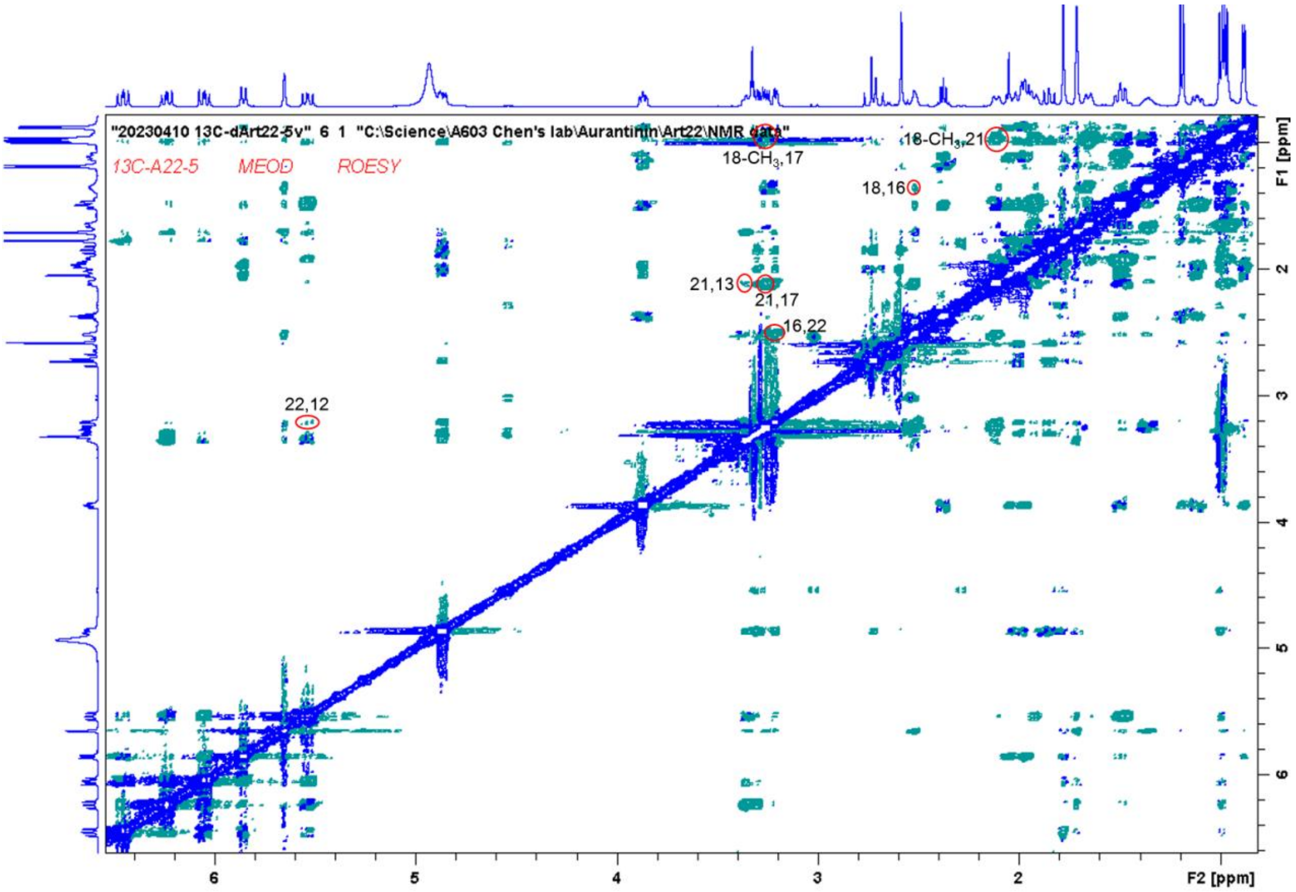

Figure S12. Spectral data of ATA 4 (**8**). (**J**) ^{1}H-^{1}H ROESY spectrum (500 MHz) of **8** in MeOH-*d*$_4$.

A

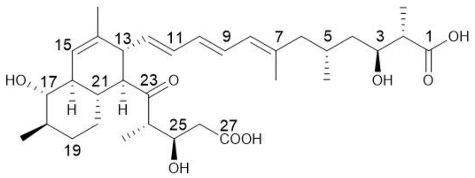

ATA 5 (**9**)

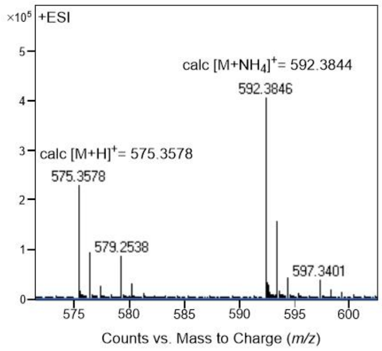

B

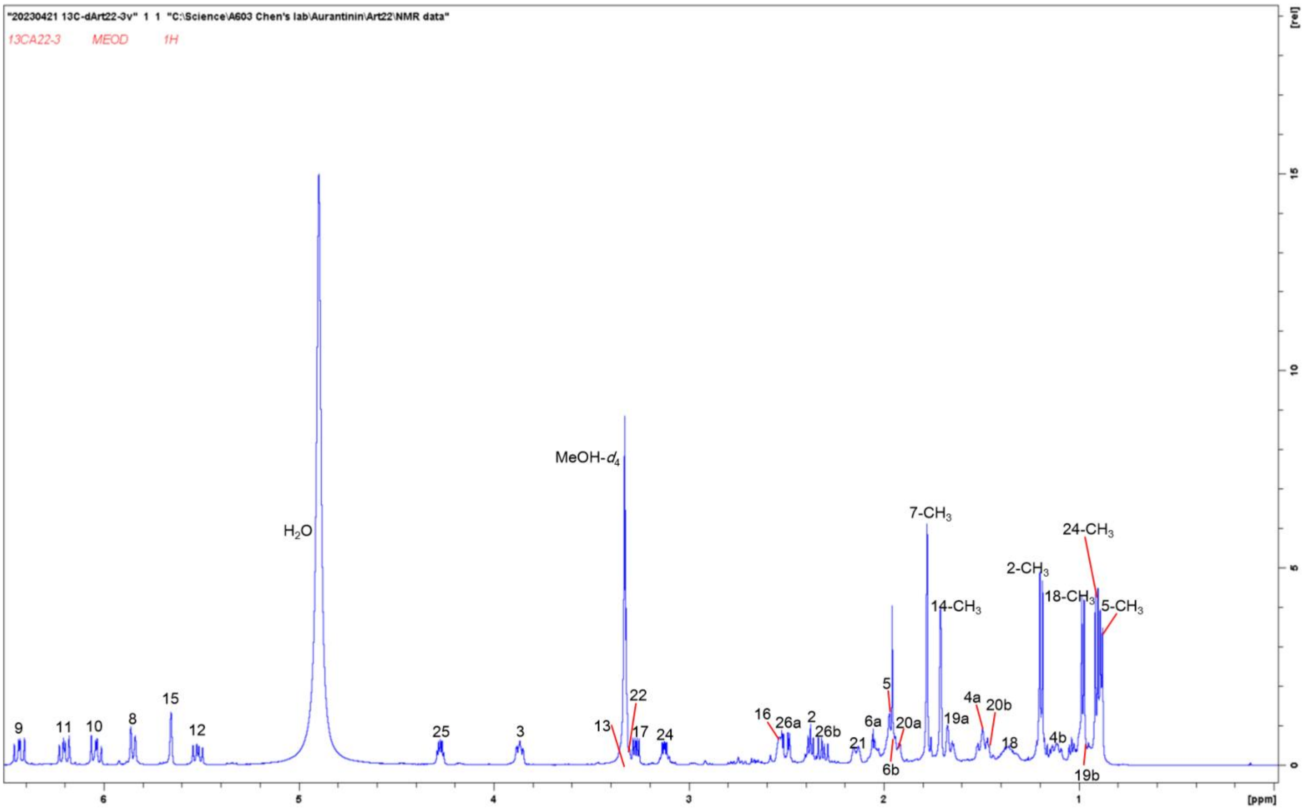

Figure S13. Spectral data of ATA 5 (**9**). (**A**) HR-ESI-MS spectrum of **9**; (**B**) ^{1}H NMR spectra (500 MHz) of **9** in MeOH-d_4.

C

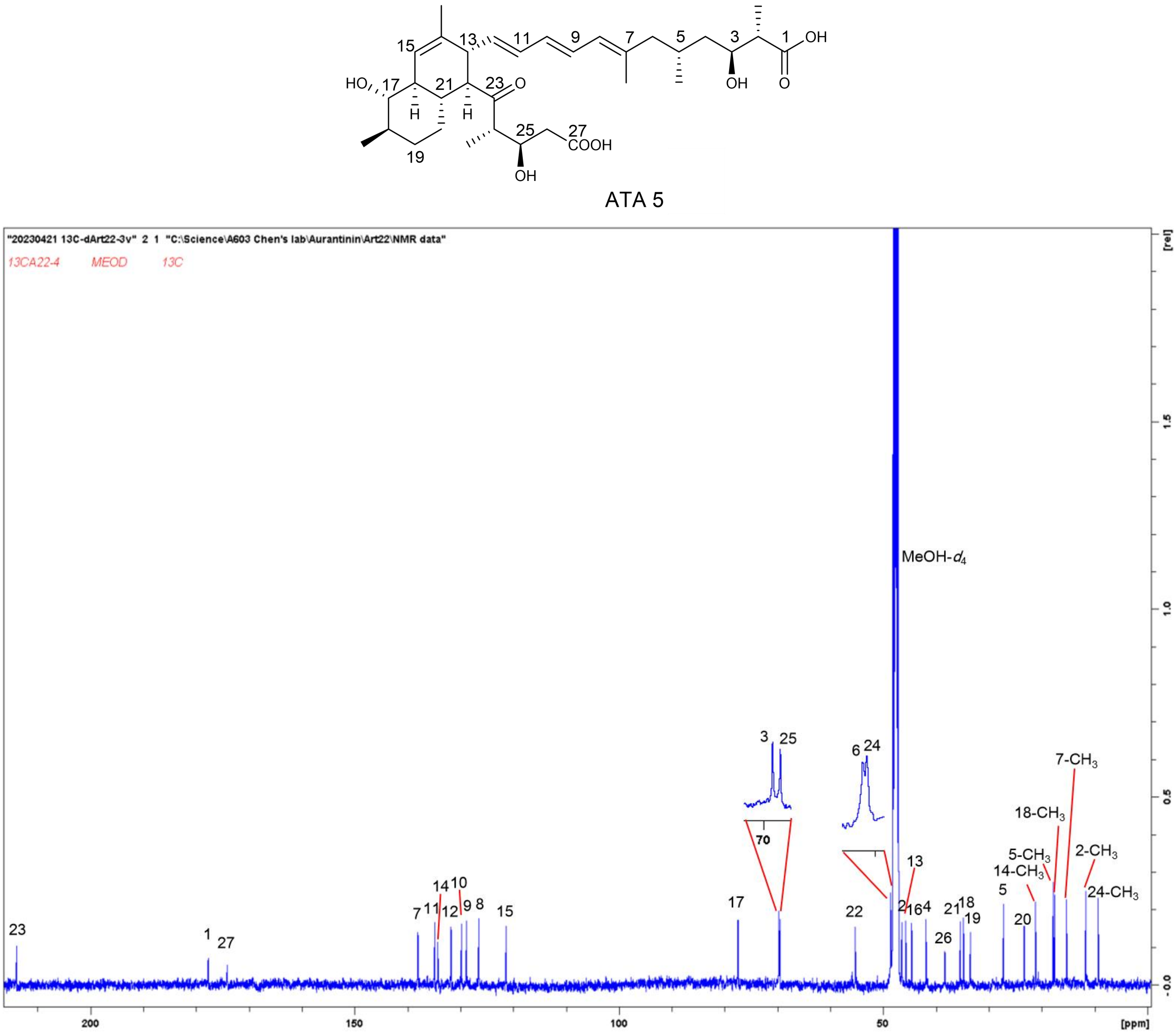

Figure S13. Spectral data of ATA 5 (**9**). (**C**) ^{13}C NMR spectrum (125 MHz) of **9** in MeOH-d_4.

D

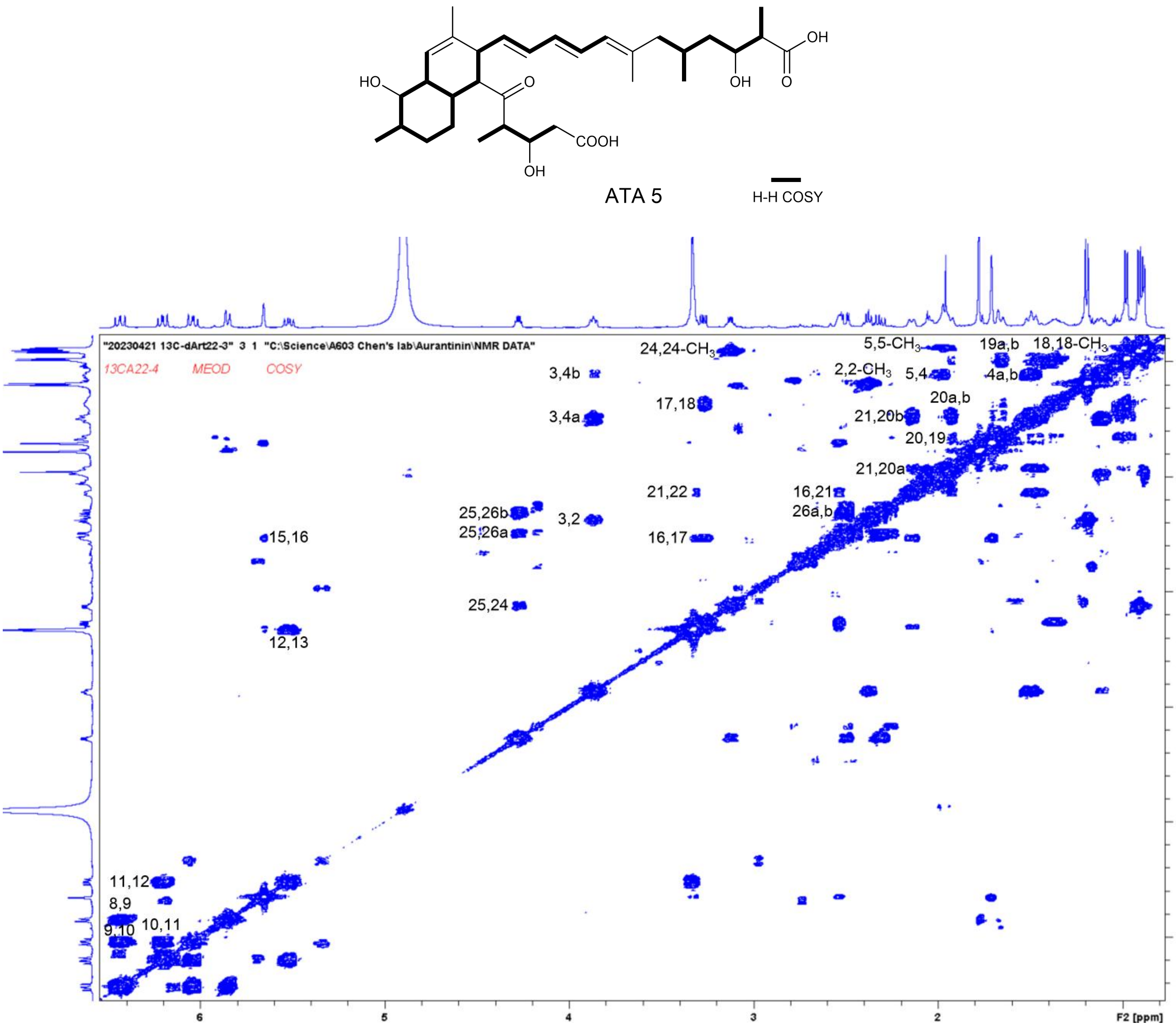

Figure S13. Spectral data of ATA 5 (**9**). (**D**) ^{1}H-^{1}H COSY spectrum (500 MHz) of **9** in MeOH-d_4.

E

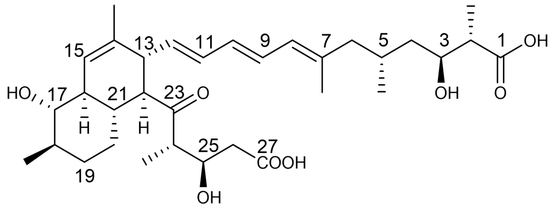

ATA 5

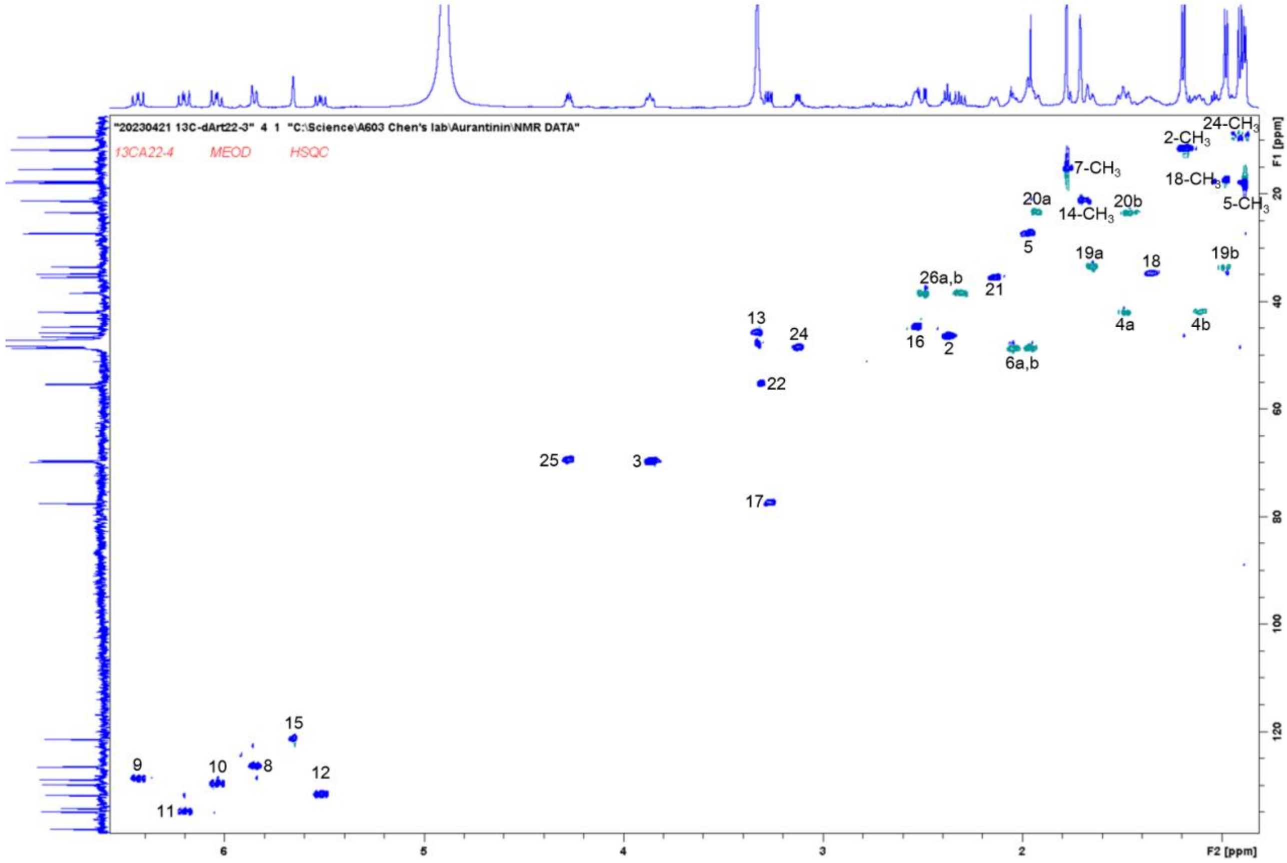

Figure S13. Spectral data of ATA 5 (**9**). (**E**) ^{1}H-^{13}C HSQC spectrum (500 MHz) of **9** in MeOH-d_4.

F

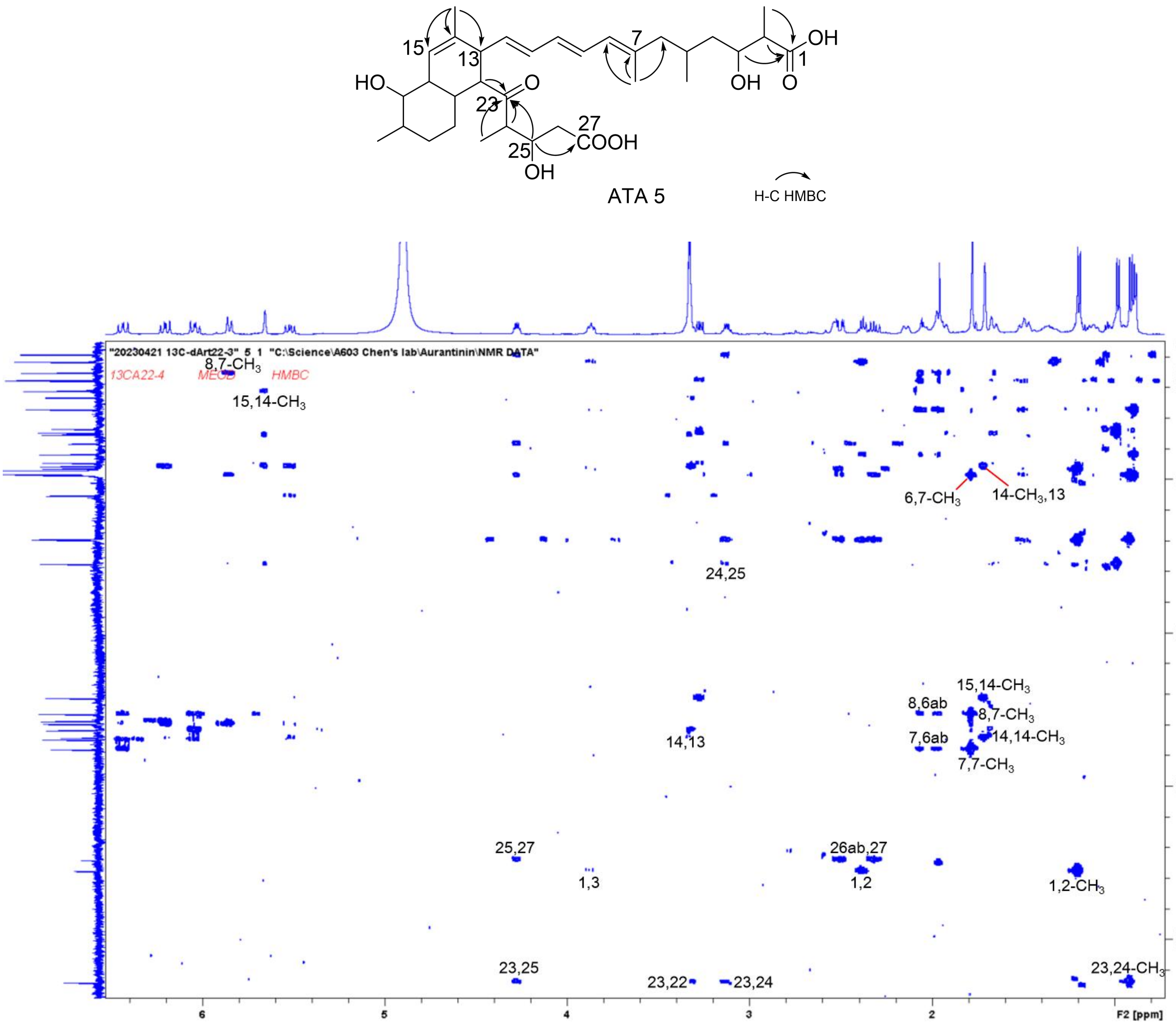

Figure S13. Spectral data of ATA 5 (**9**). (**F**) ^{1}H-^{13}C HMBC spectrum (500 MHz) of **9** in MeOH-d_4.

G

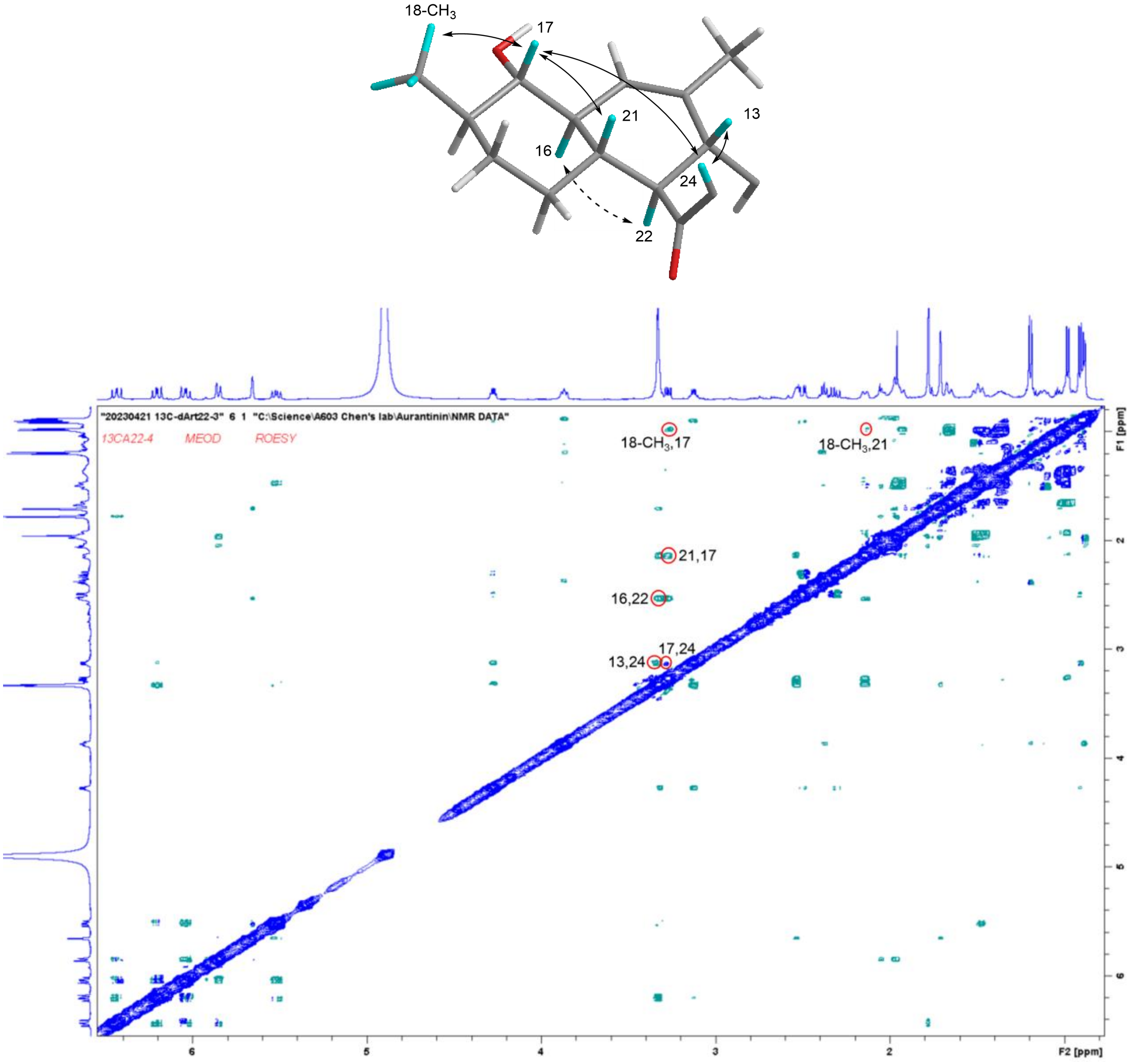

Figure S13. Spectral data of ATA 5 (**9**). (**G**) ^{1}H-^{1}H ROESY spectrum (500 MHz) of **9** in MeOH-d_4.

H

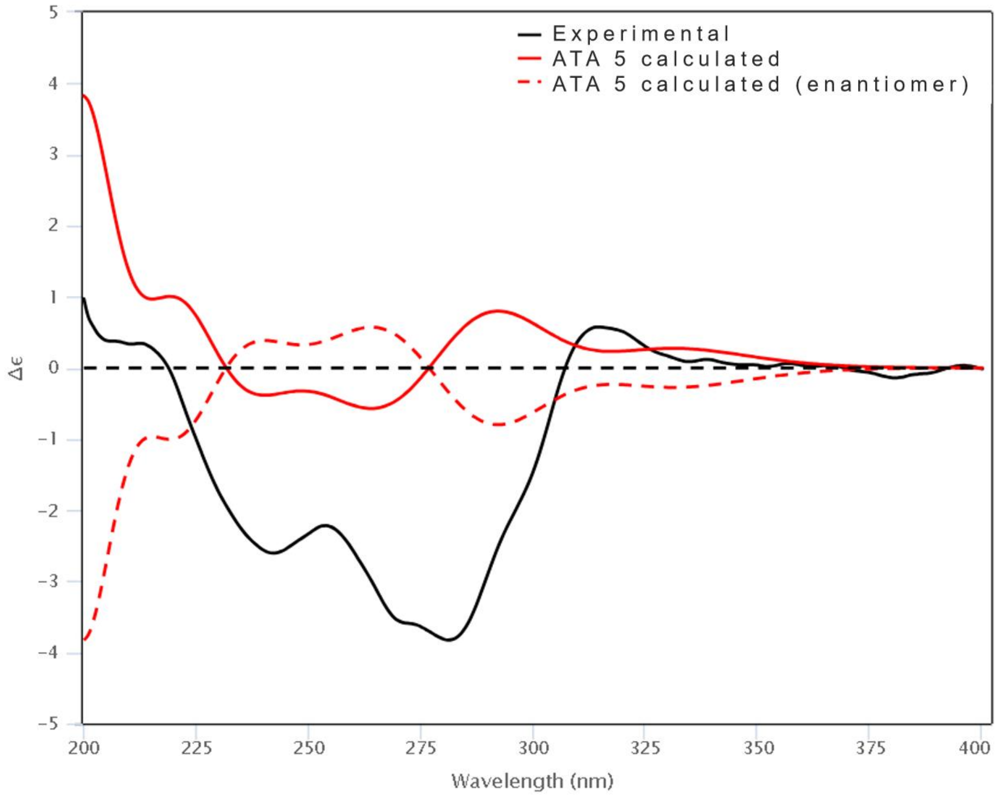

Figure S13. Spectral data of ATA 5 (**9**). (**H**) Experimental ECD spectrum of **9** in acetonitrile was compared with its theoretical ECD (bandwidth σ = 0.3 eV, uv shift = 10 nm).

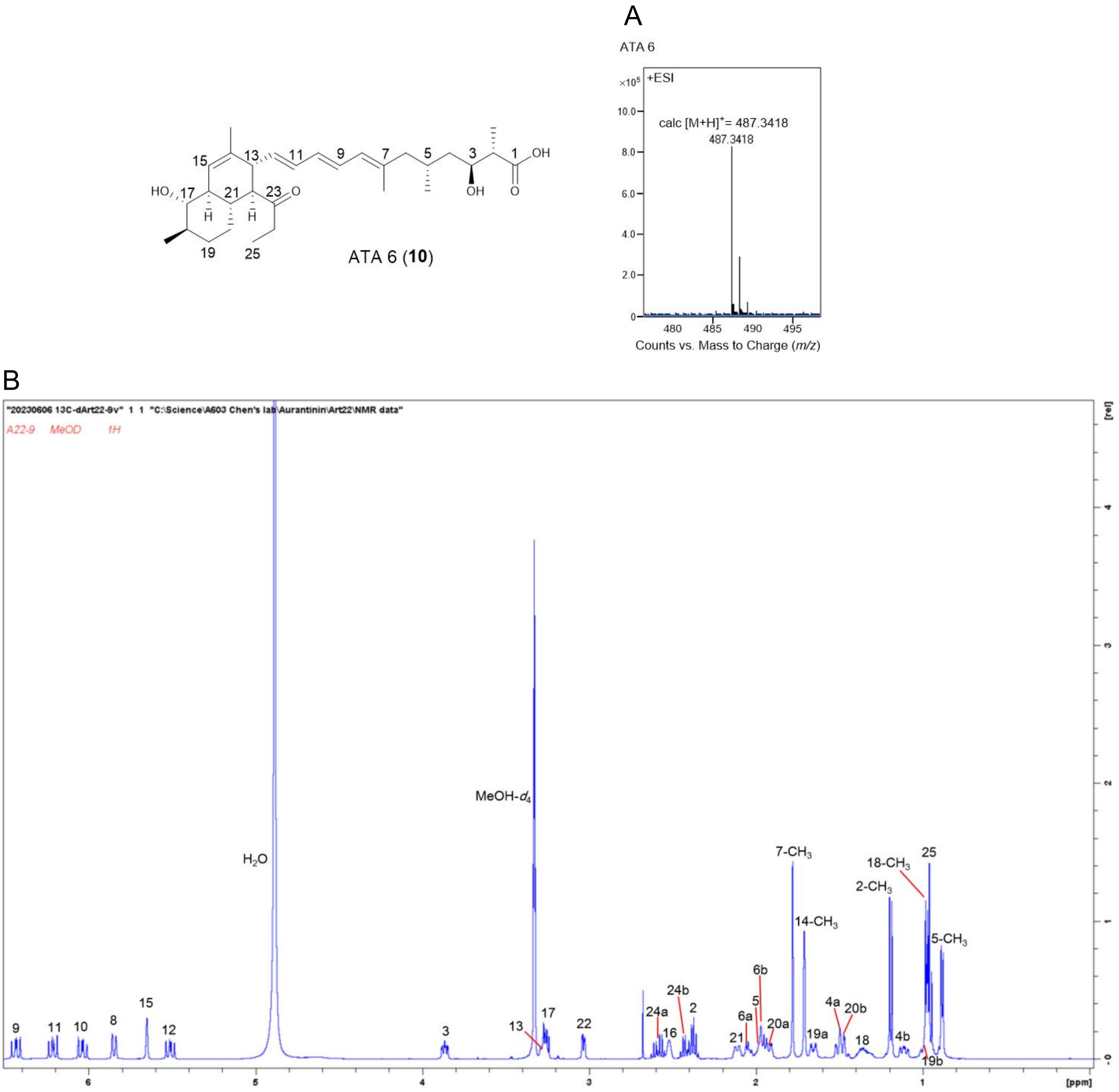

Figure S14. Spectral data of ATA 6 (**10**). (**A**) HR-ESI-MS spectrum of **10**. (**B**) ^{1}H NMR spectra (500 MHz) of **10** in MeOH-d_4.

C

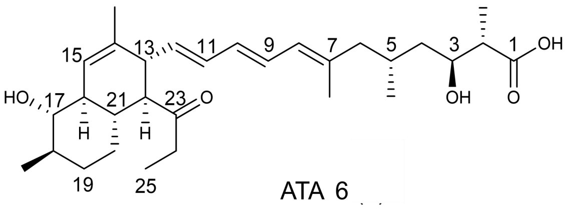

ATA 6

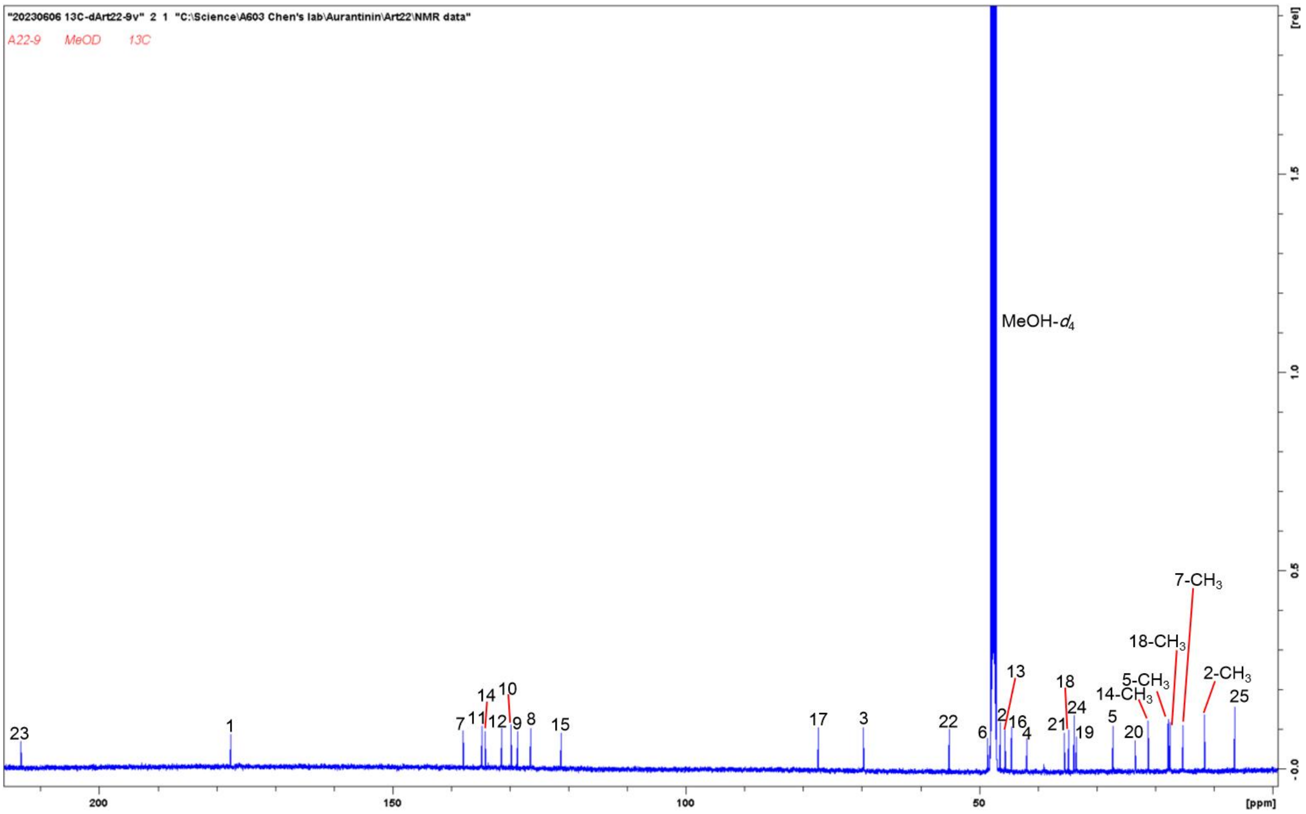

Figure S14. Spectral data of ATA 6 (**10**). (**C**) ^{13}C NMR spectrum (125 MHz) of **10** in MeOH-d_4.

D

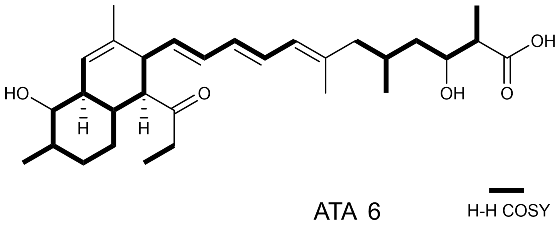

ATA 6

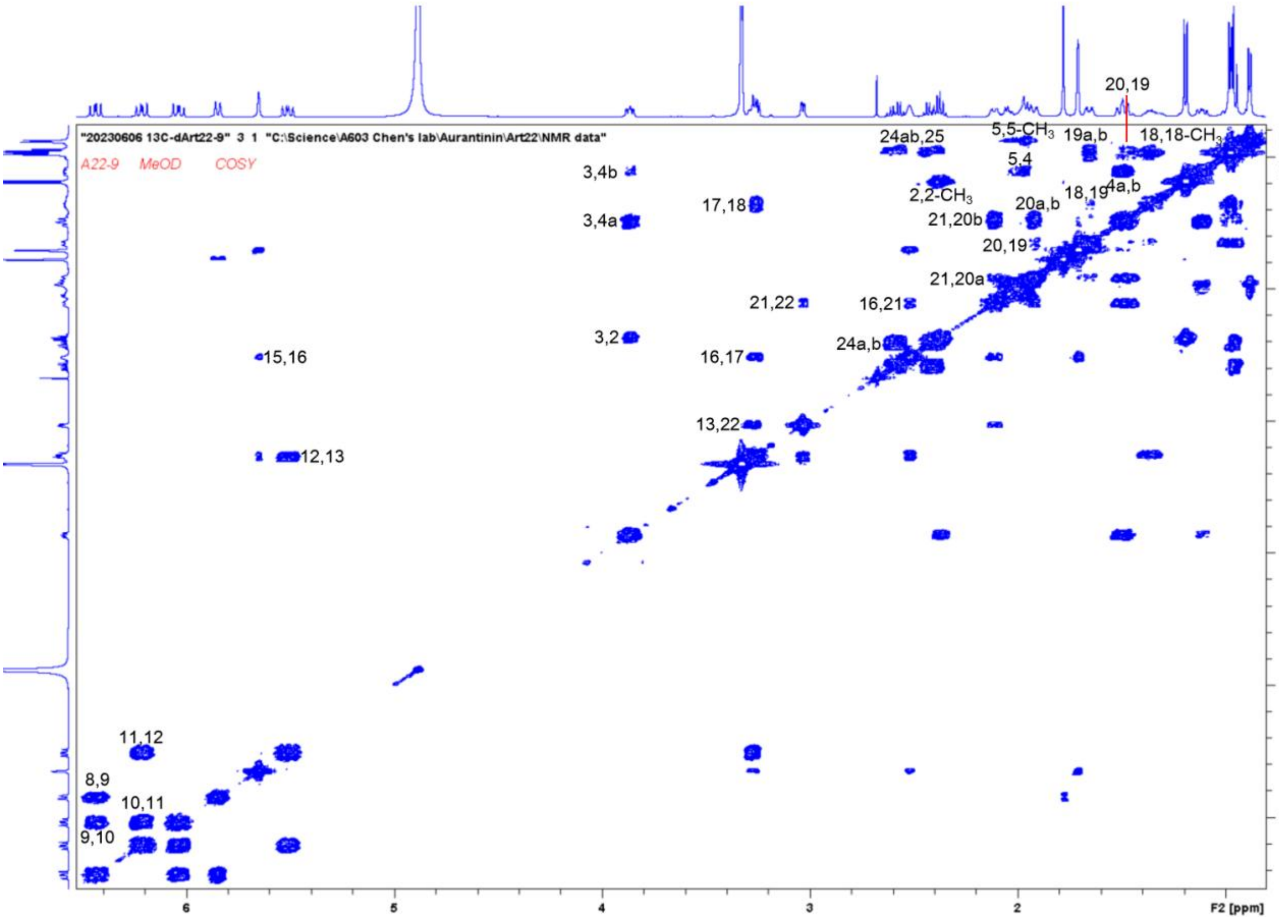

Figure S14. Spectral data of ATA 6 (**10**). (**D**) ^{1}H-^{1}H COSY spectrum (500 MHz) of **10** in MeOH-d_4.

E

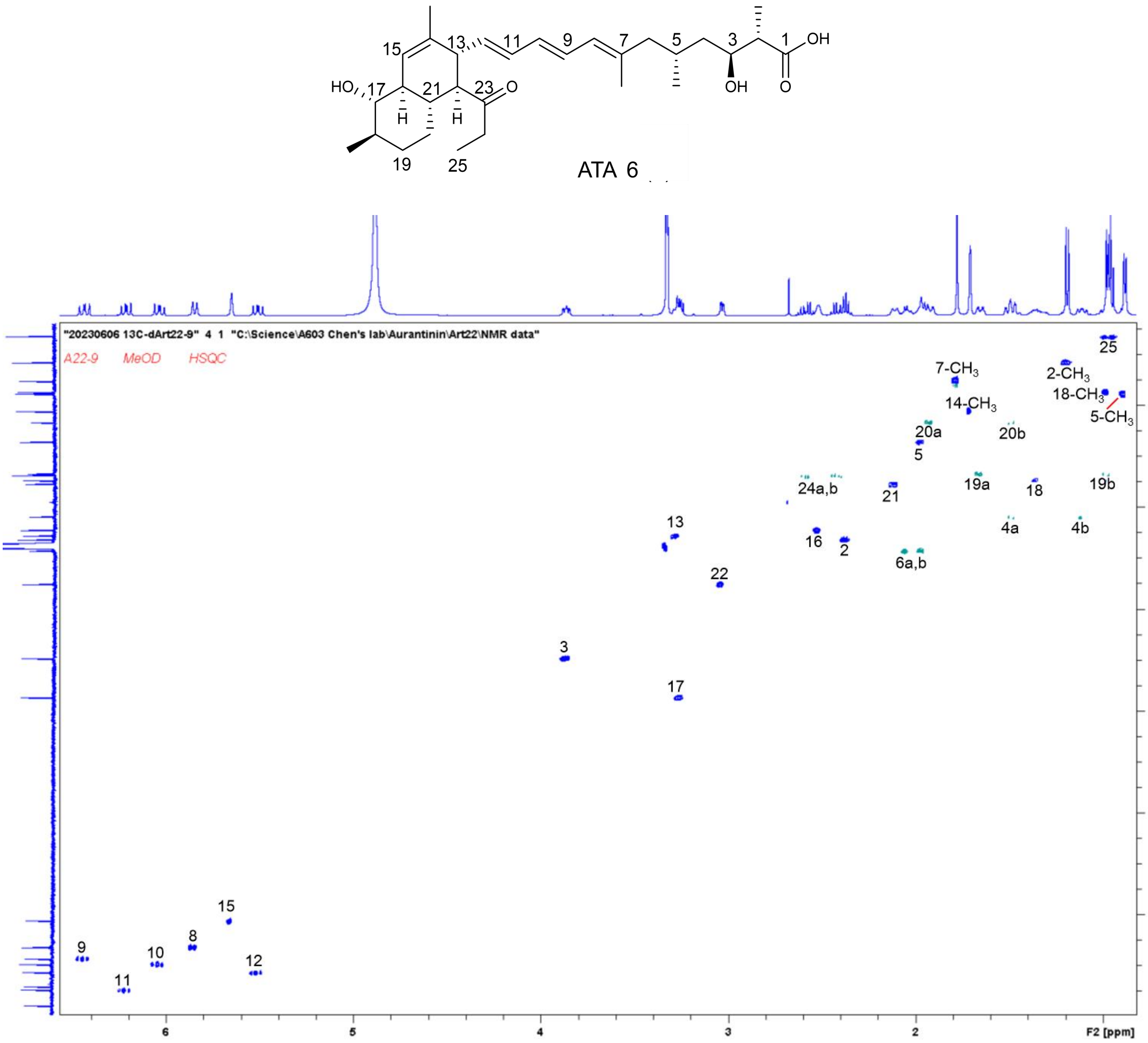

Figure S14. Spectral data of ATA 6 (**10**). (**E**) ^{1}H-^{13}C HSQC spectrum (500 MHz) of **10** in MeOH-d_4.

F

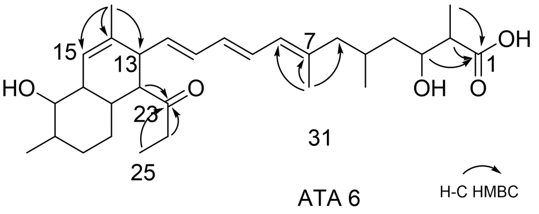

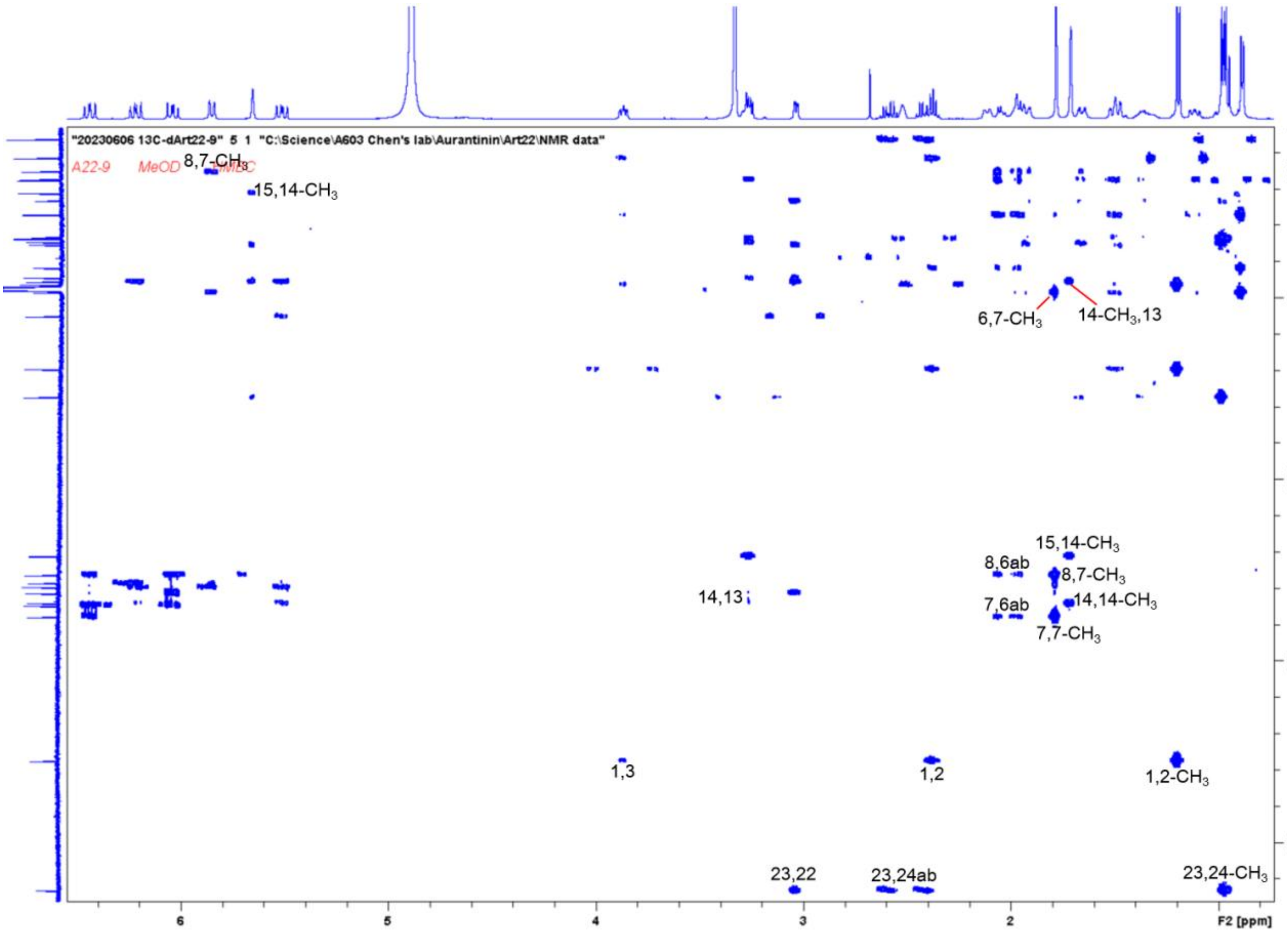

Figure S14. Spectral data of ATA 6 (**10**). (**F**) ^{1}H-^{13}C HMBC spectrum (500 MHz) of **10** in MeOH-d_4.

G

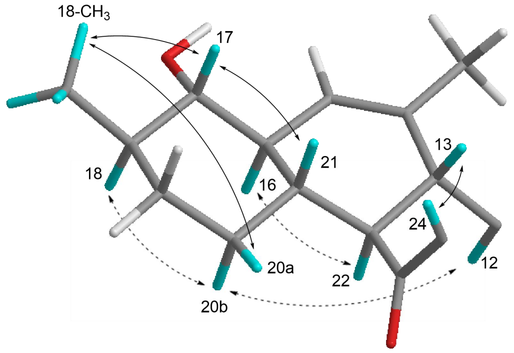

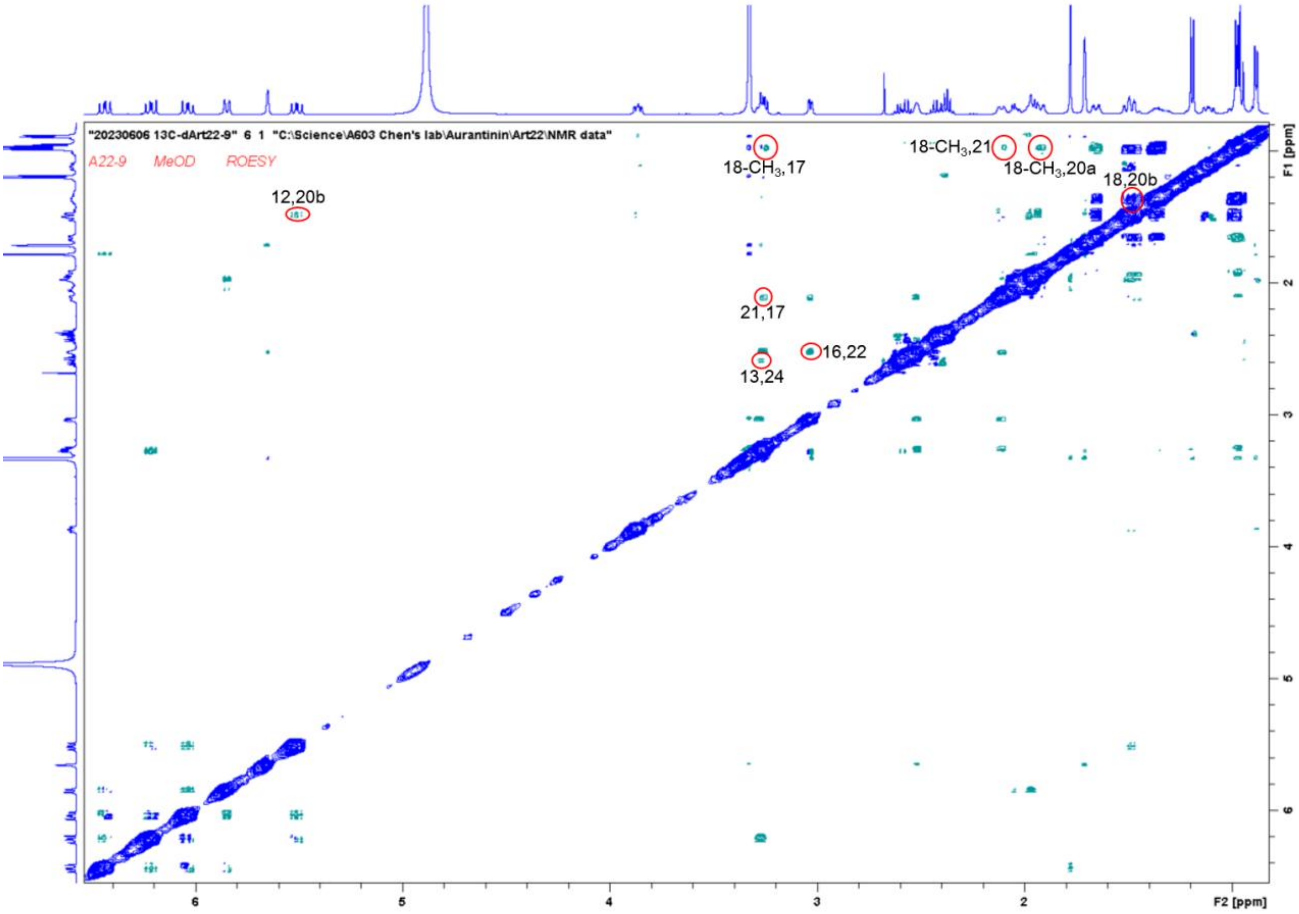

Figure S14. Spectral data of ATA 6 (**10**). (**G**) ^{1}H-^{1}H ROESY spectrum (500 MHz) of **10** in MeOH-*d*4.

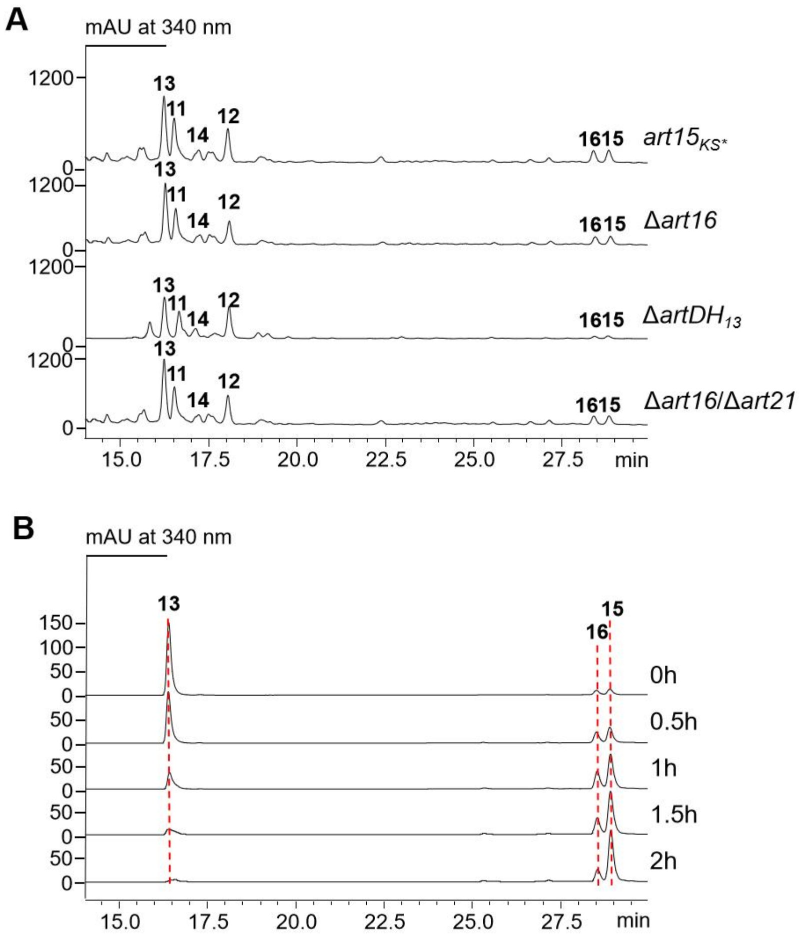

Figure S15. Evaluation of the stability of compound **13**. (**A**) HPLC analysis of *B. Subtilis* mutant strains revealed the production of **15** and **16** during the fermentation process. (**B**) The stability of **13** was checked by time-course analysis in a solution containing 1:1 of H_2O and acetonitrile with 0.5‰ of formic acid. Compound **13** was proposed to undergo spontaneous lactonization to yield **15** and **16**.

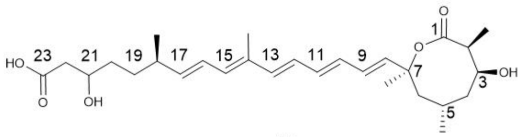

15

A

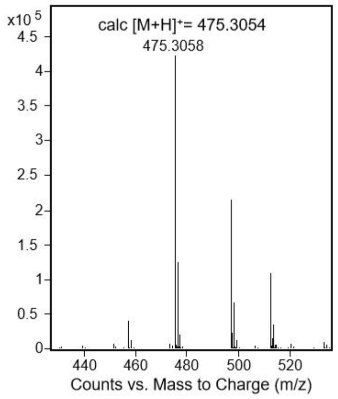

B

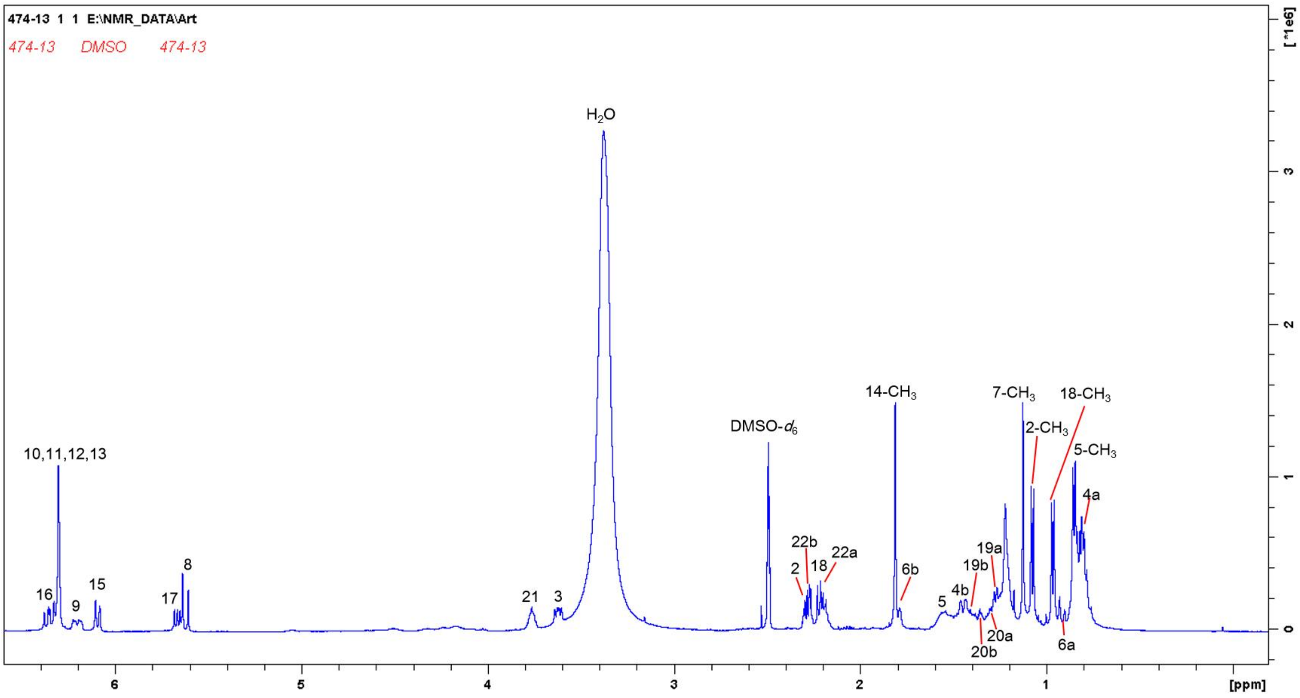

Figure S16. Spectral data of **15**. (**A**) HR-ESI-MS spectrum of compound **15**; (**B**) ^{1}H NMR spectra (500 MHz) of **15** in DMSO-d_6.

C

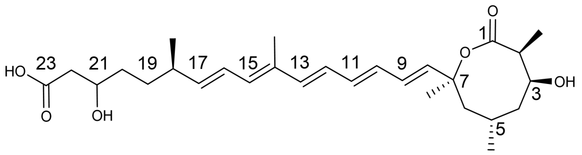

15

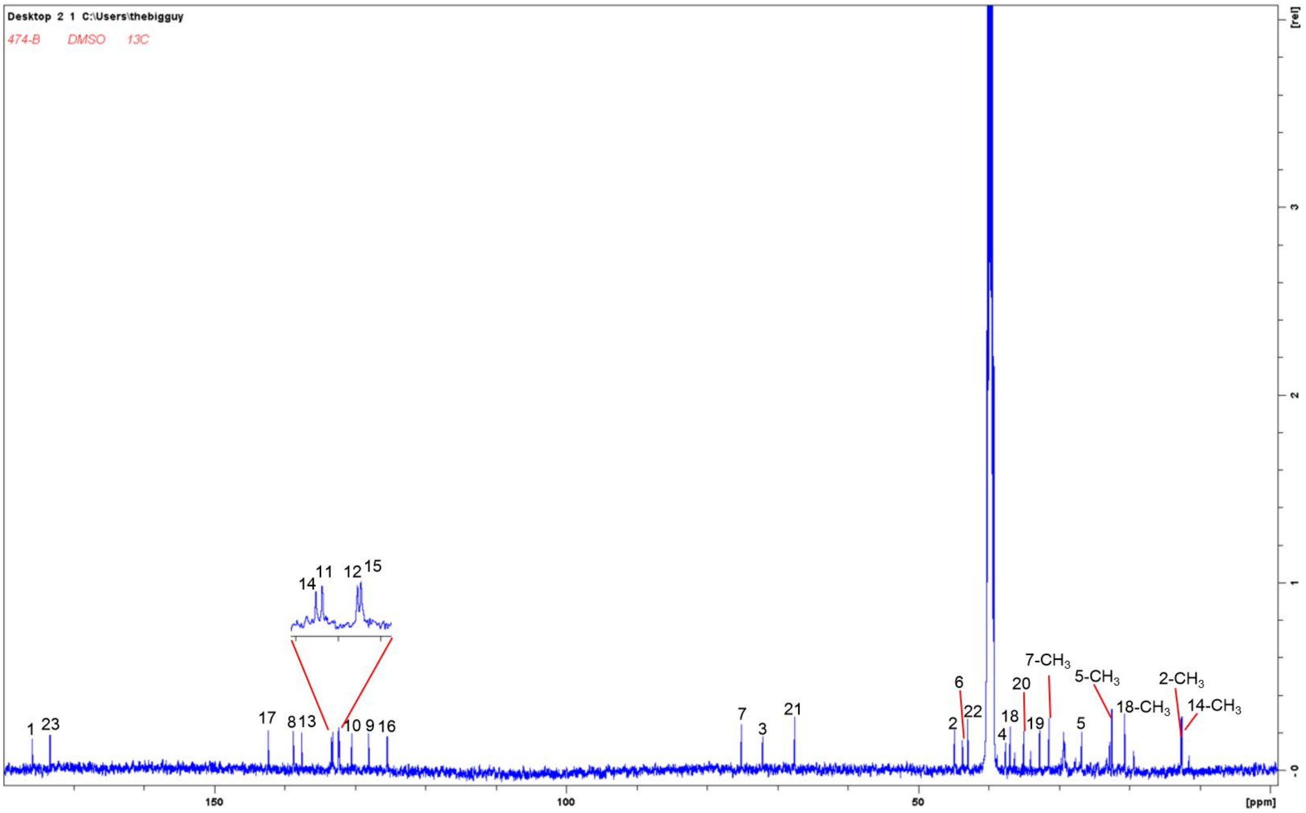

Figure S16. Spectral data of **15**. (**C**) ^{13}C NMR spectrum (125 MHz) of compound **15** in DMSO-d_6.

D

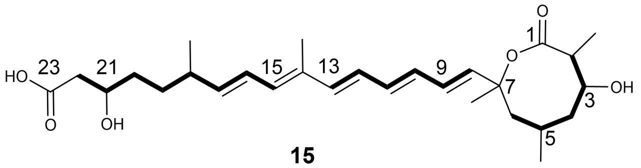

15

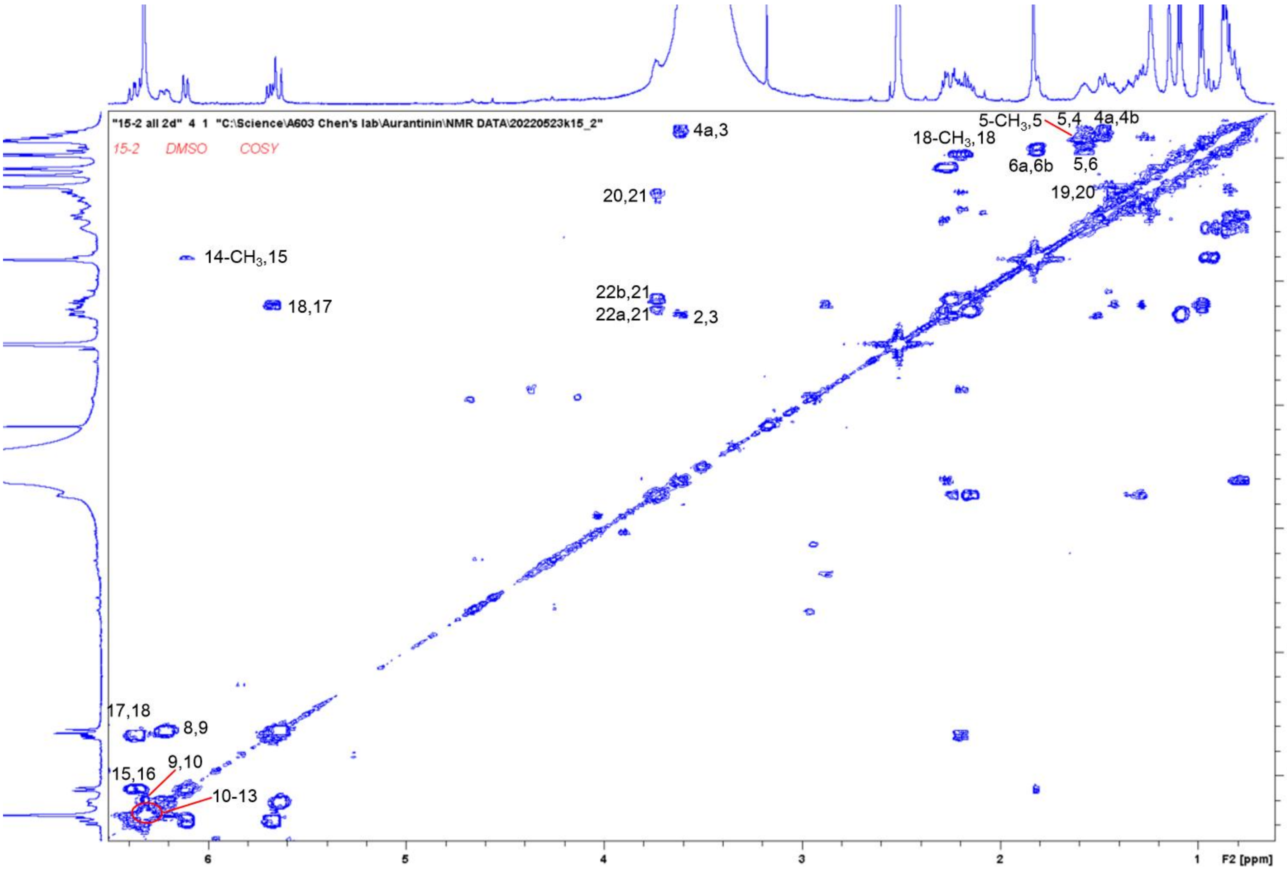

Figure S16. Spectral data of **15**. (**D**) ^{1}H-^{1}H COSY spectrum (500 MHz) of compound **15** in DMSO-d_6.

E

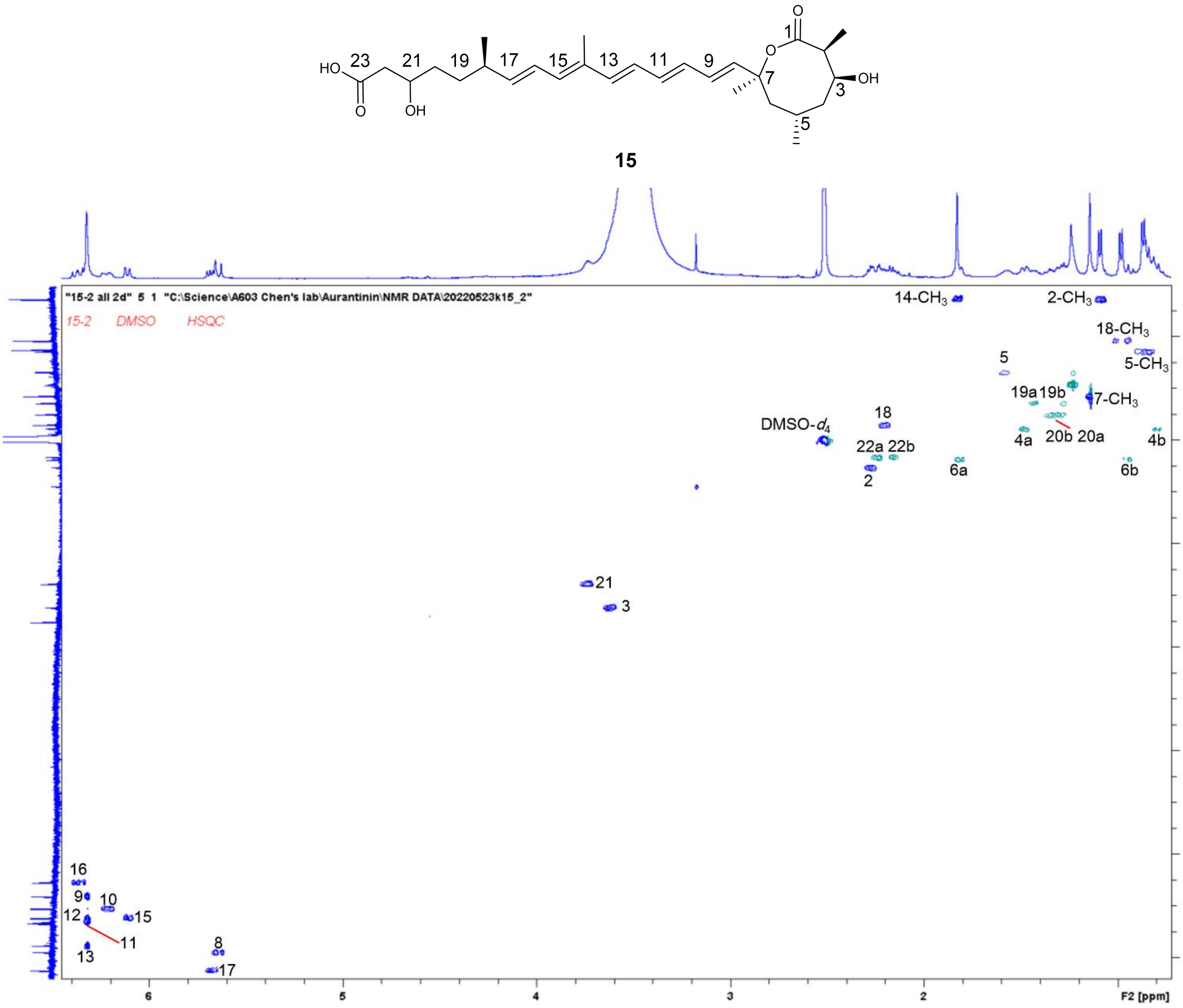

Figure S16. Spectral data of **15**. (**E**) ^{1}H-^{13}C HSQC spectrum (500 MHz) of compound **15** in DMSO-d_6.

F

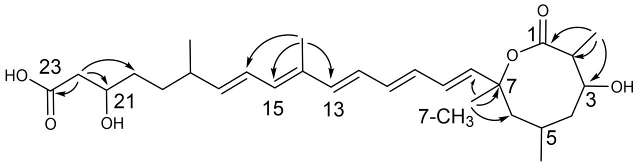

15

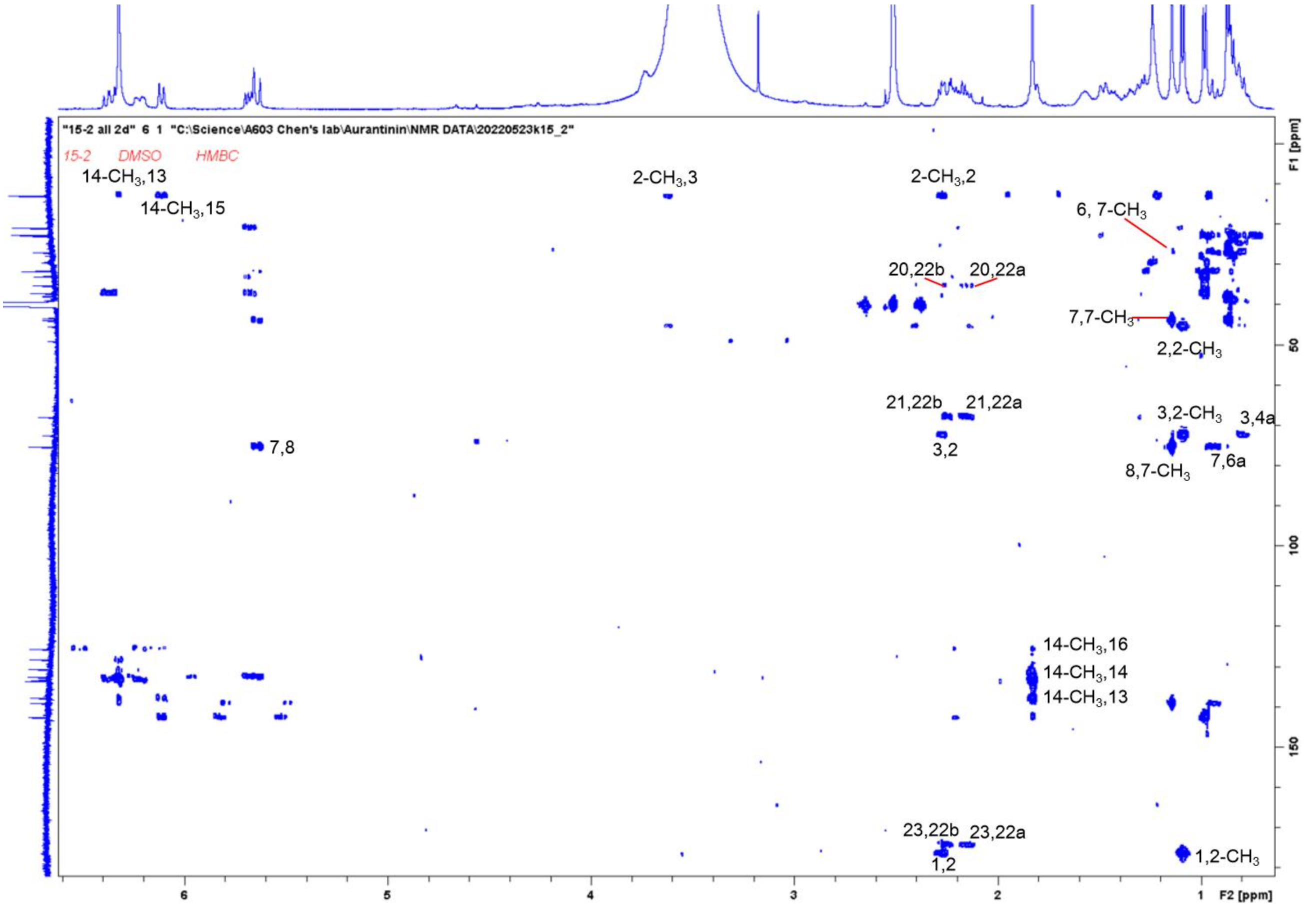

Figure S16. Spectral data of **15**. (**F**) ^{1}H-^{13}C HMBC spectrum (500 MHz) of compound **15** in DMSO-d_6.

G

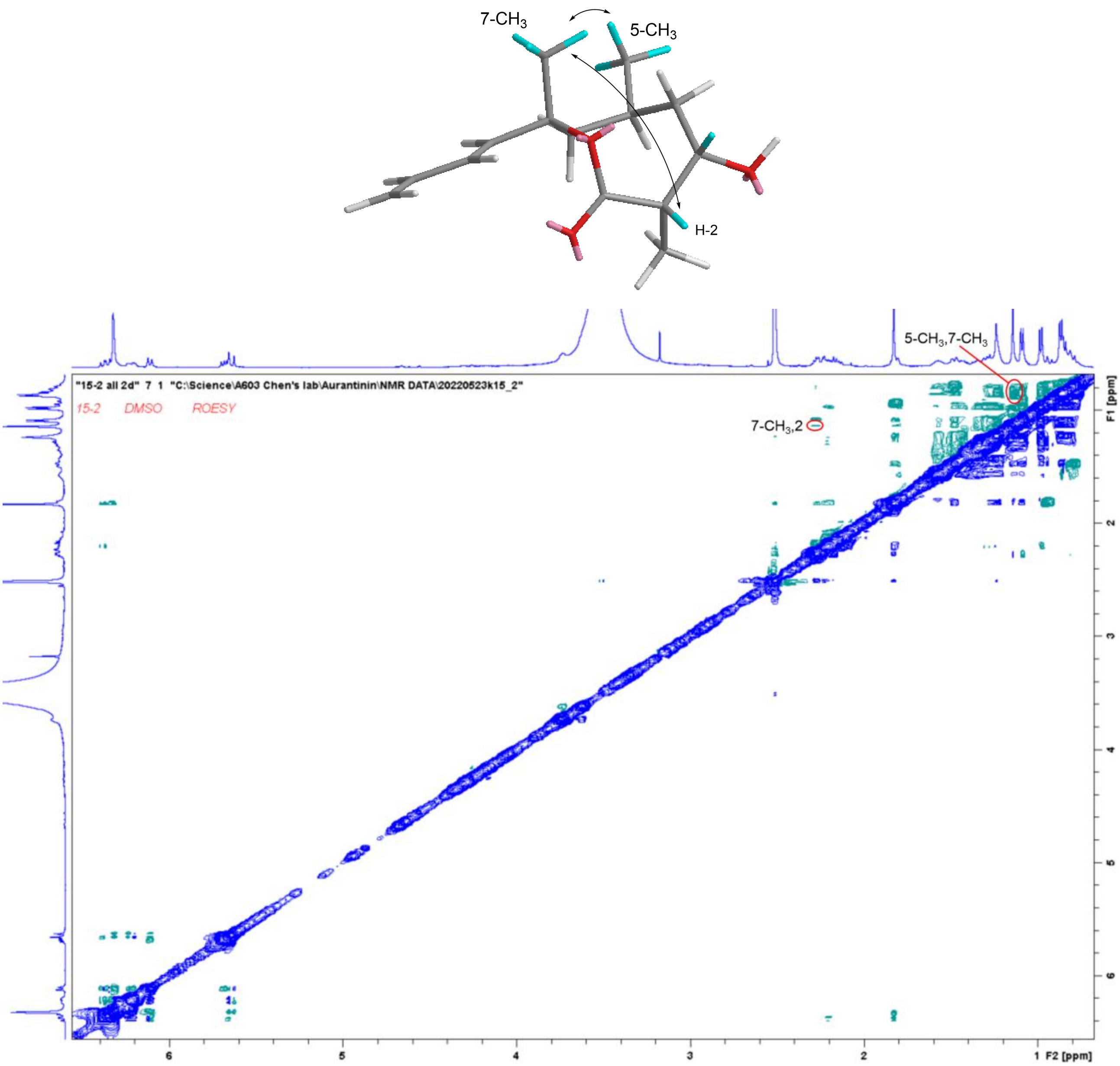

Figure S16. Spectral data of **15**. (**G**) ^{1}H-^{1}H ROESY spectrum (500 MHz) of compound **15** in DMSO-d_6.

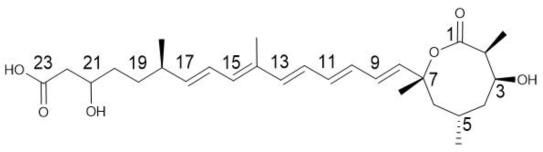

16

A

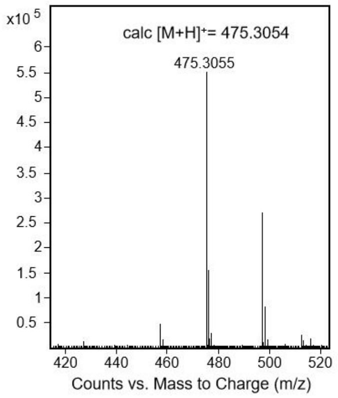

B

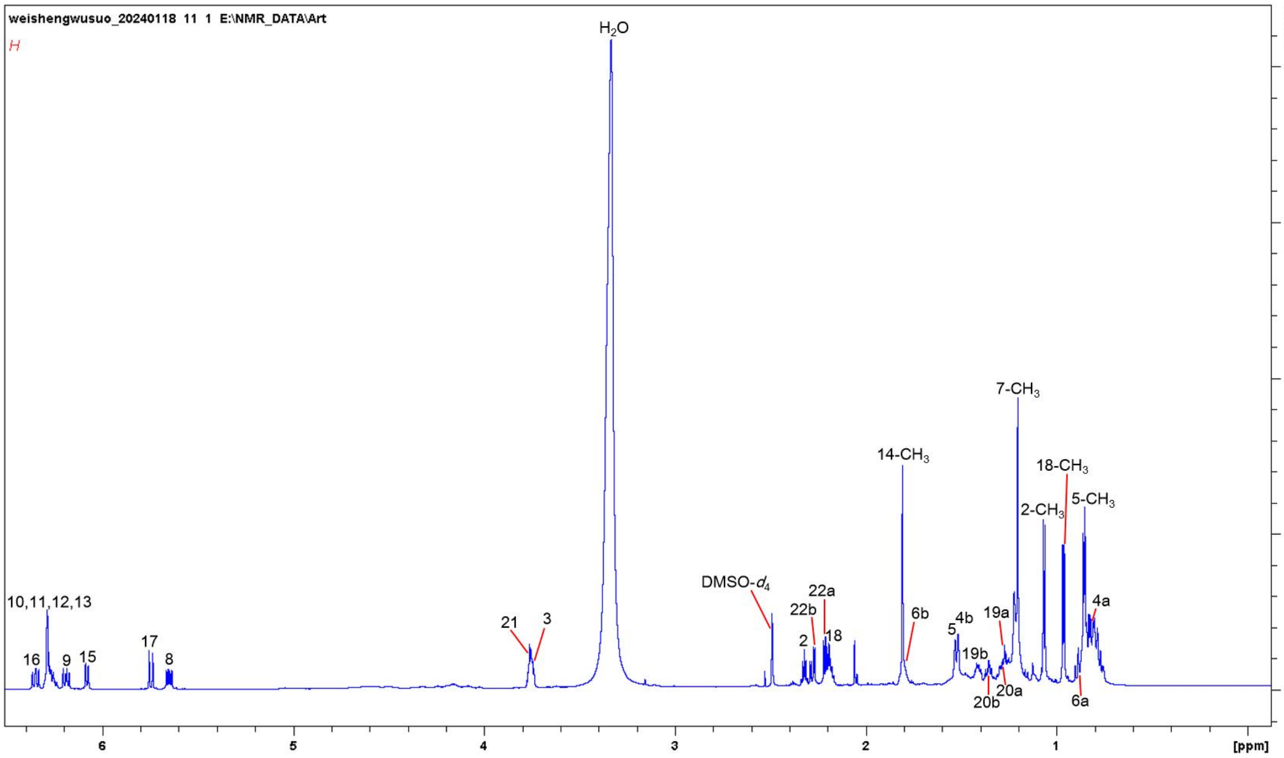

Figure S17. Spectral data of **16**. (**A**) HR-ESI-MS spectrum of compound **16**; (**B**) ^{1}H NMR spectra (800 MHz) of **16** in DMSO-d_6.

C

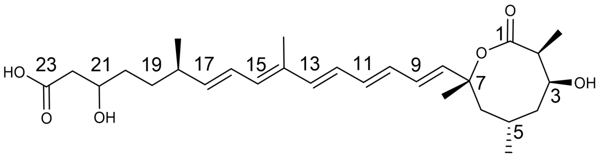

16

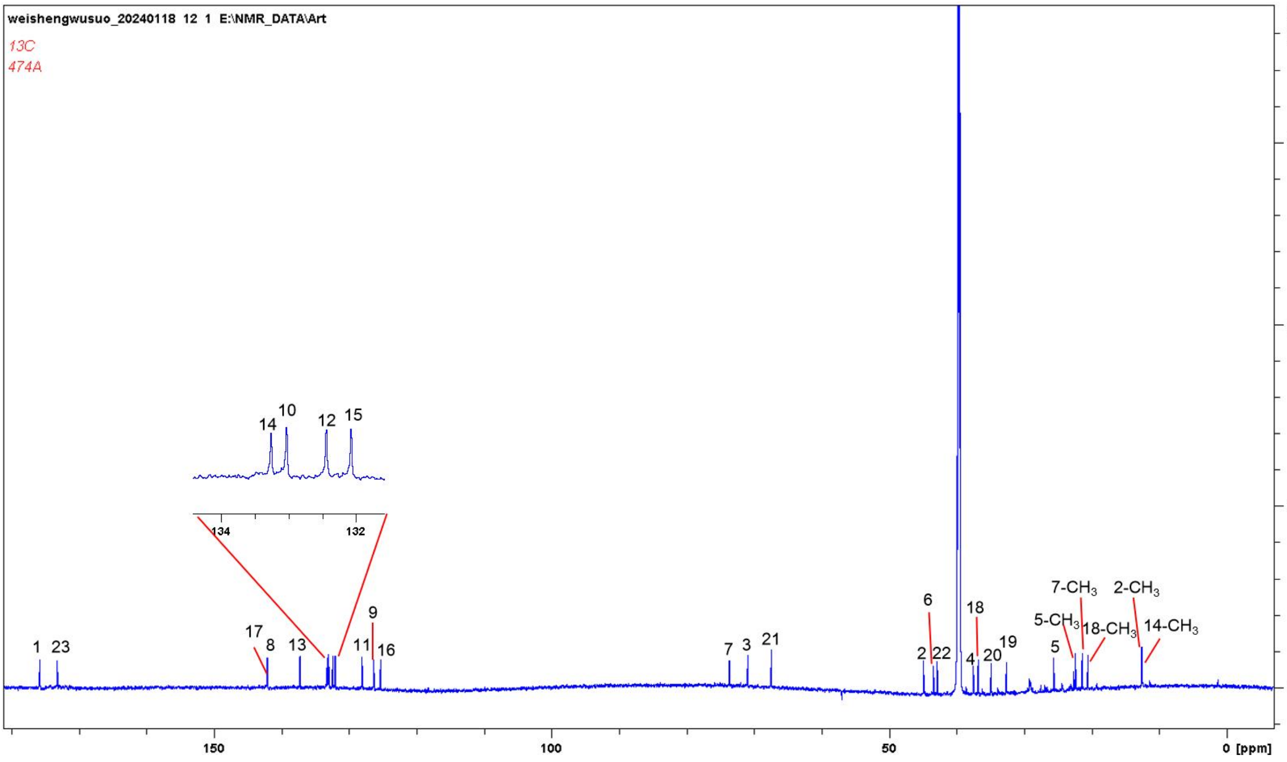

Figure S17. Spectral data of **16**. (**C**) ^{13}C NMR spectrum (200 MHz) of compound **16** in DMSO-d_6.

D

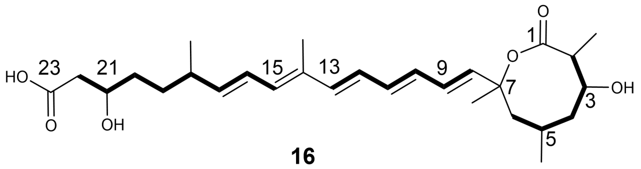

16

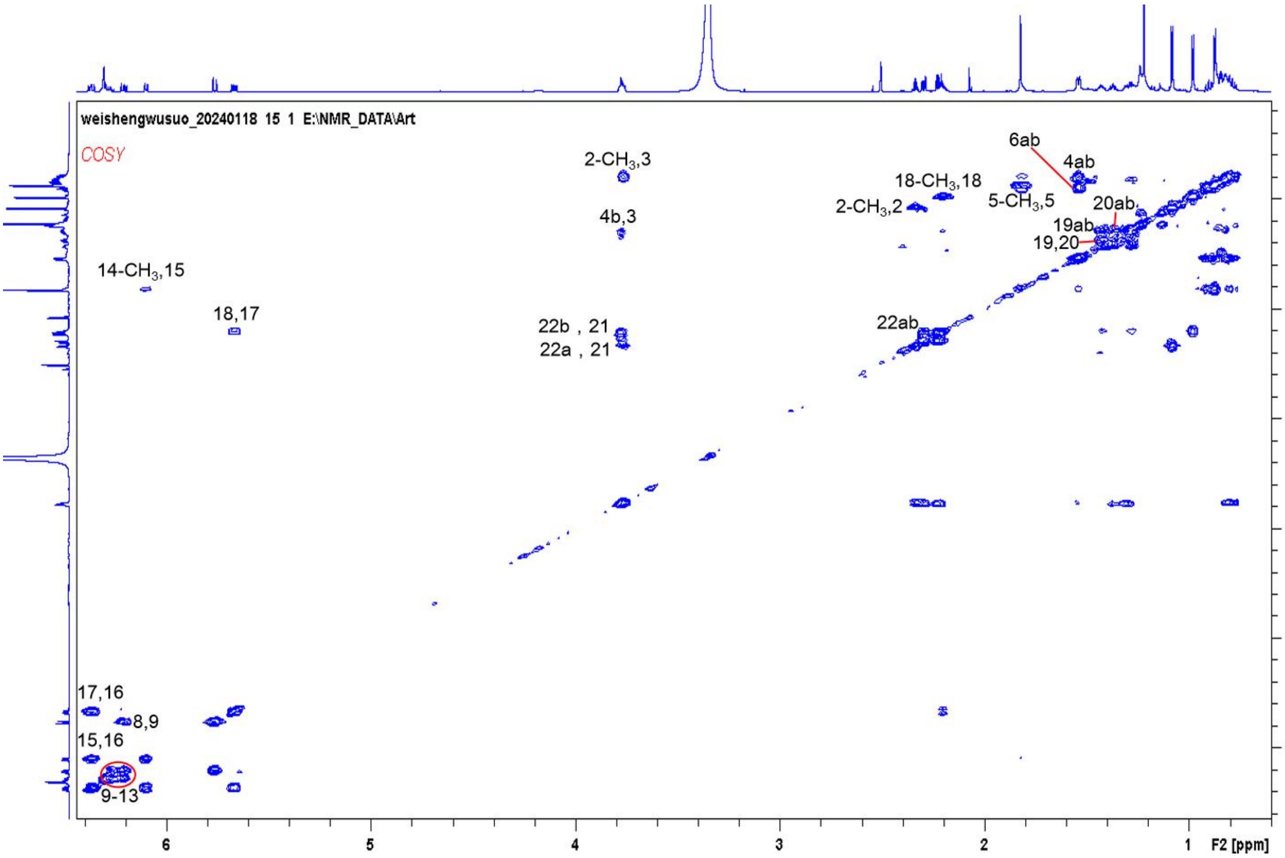

Figure S17. Spectral data of **16**. (**D**) ^{1}H-^{1}H COSY spectrum (800 MHz) of compound **16** in DMSO-d_6.

E

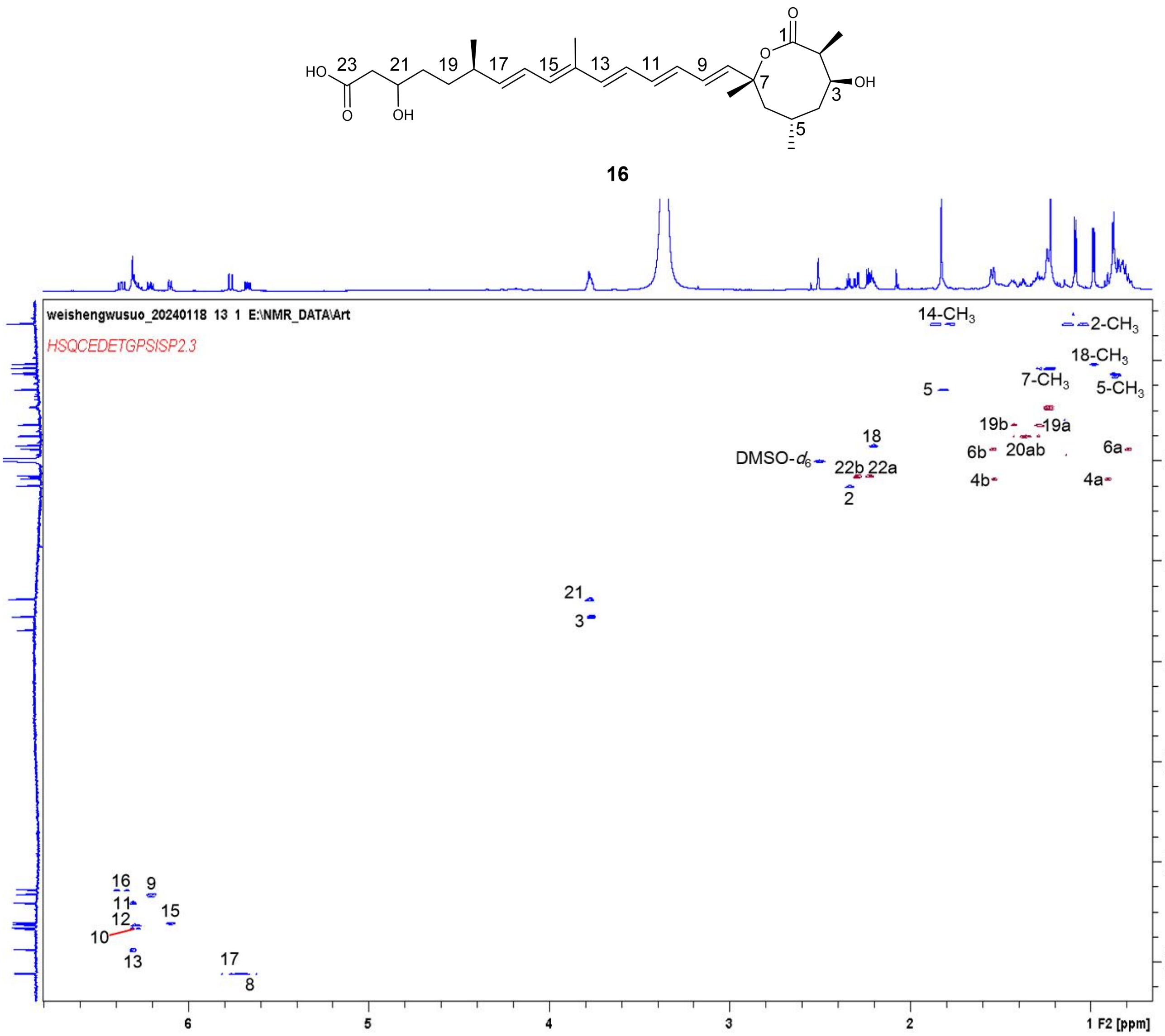

Figure S17. Spectral data of **16**. (**E**) ^{1}H-^{13}C HSQC spectrum (800 MHz) of compound **16** in DMSO-d_6.

F

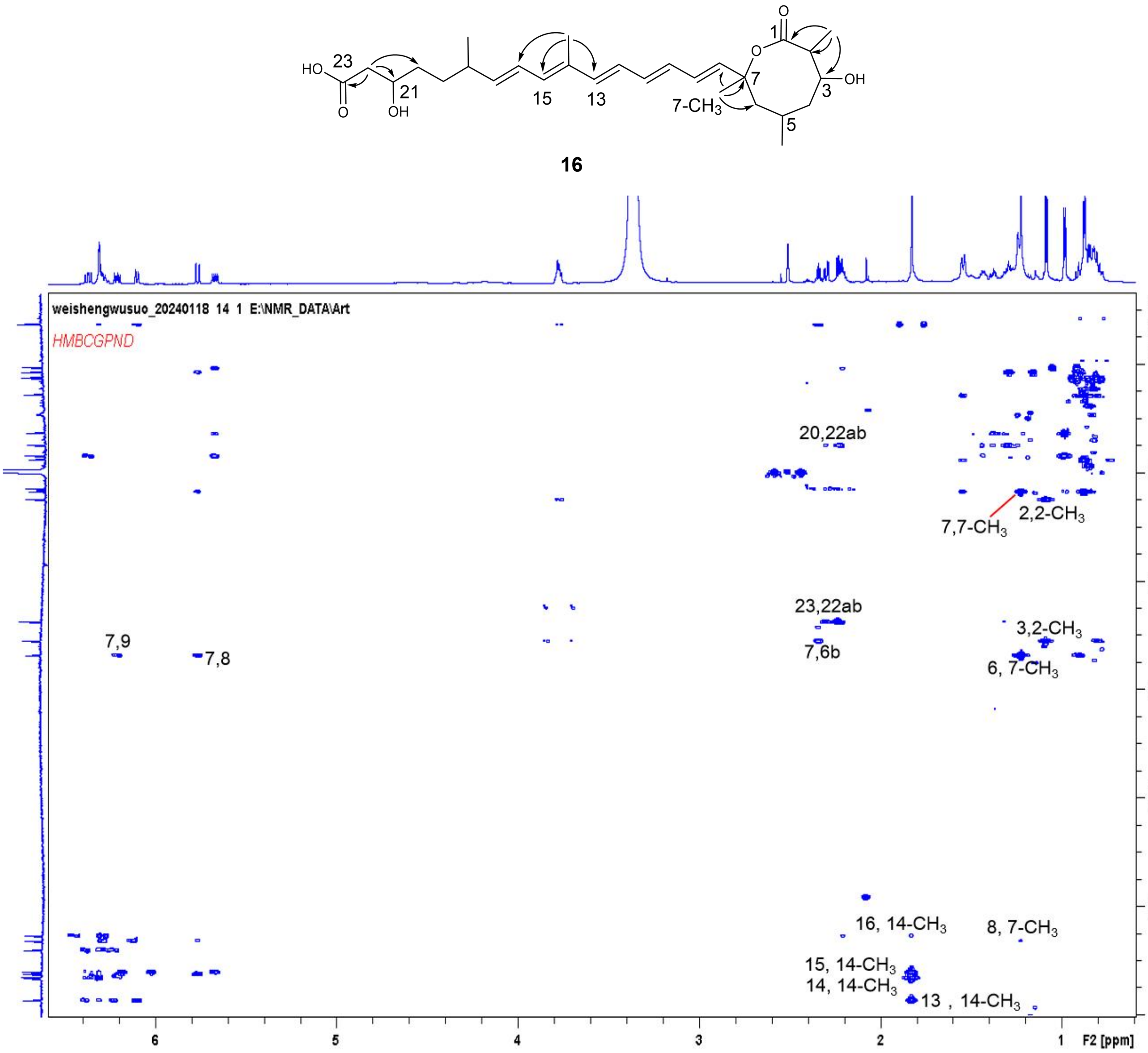

Figure S17. Spectral data of **16**. (**F**) ^{1}H-^{13}C HMBC spectrum (800 MHz) of compound **16** in DMSO-d_6.

G

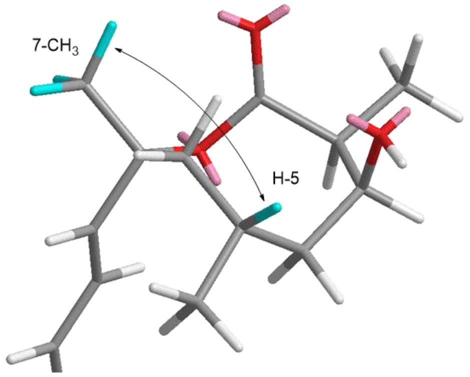

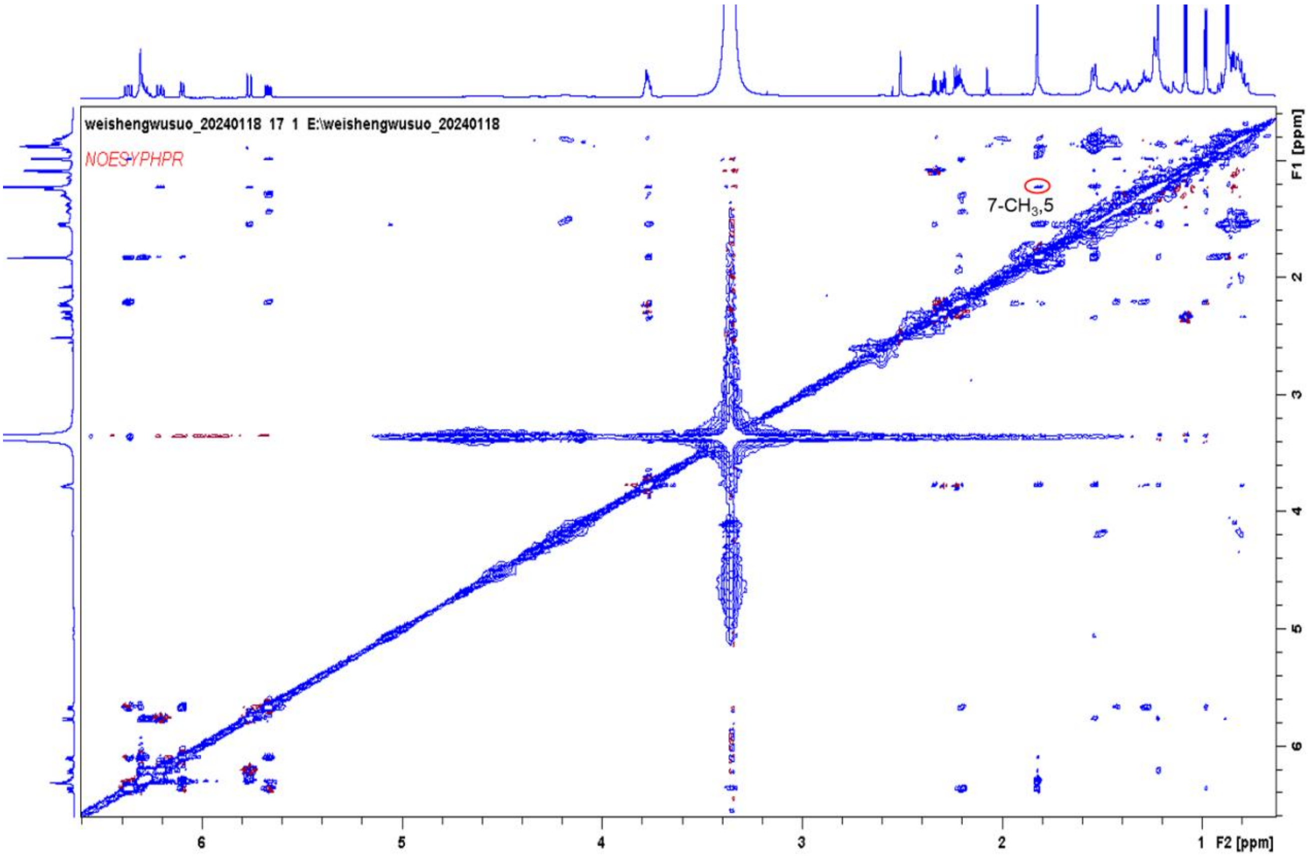

Figure S17. Spectral data of **16**. (**G**) ^{1}H-^{1}H ROESY spectrum (800 MHz) of compound **16** in DMSO-d_6.

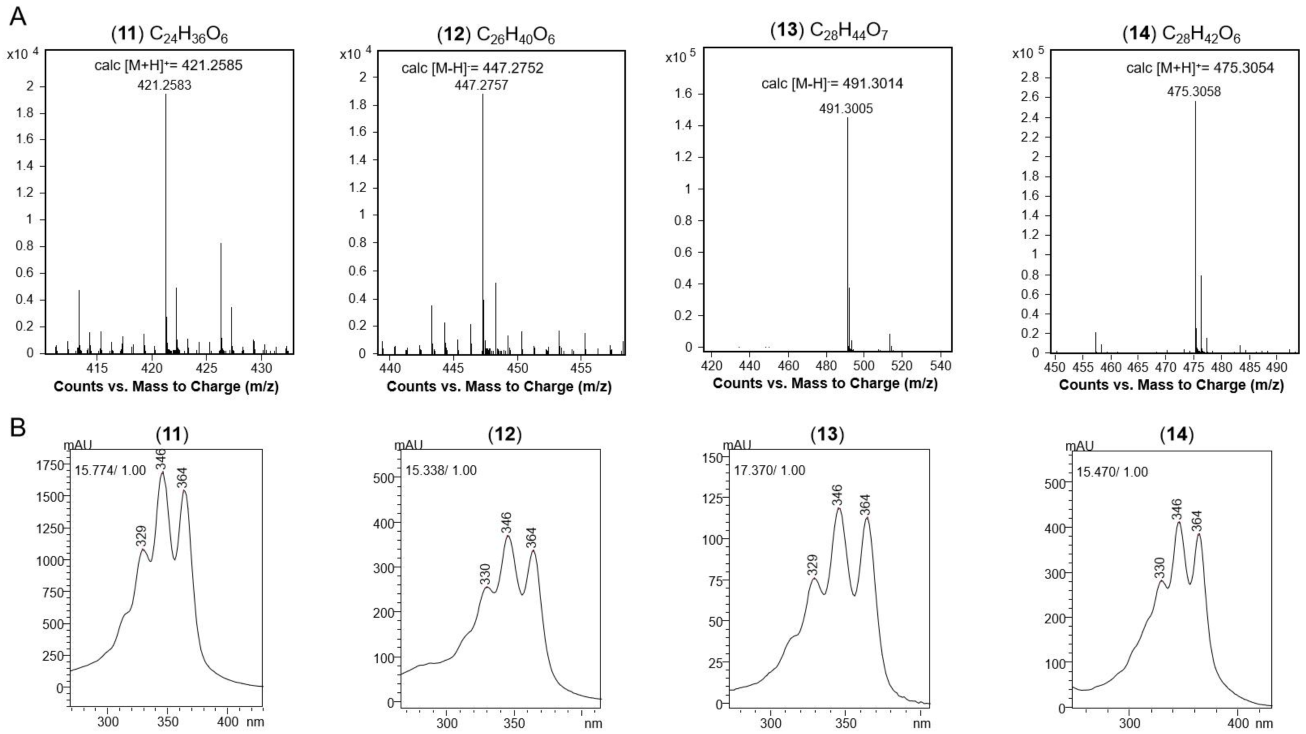

Figure S18. Absorbance and HR-ESI-MS data of compounds **11-14**. (**A**) HR-ESI-MS spectra of compounds **11-14**; (**B**) UV-visible absorptions of the pentaene-containing products **11-14**.

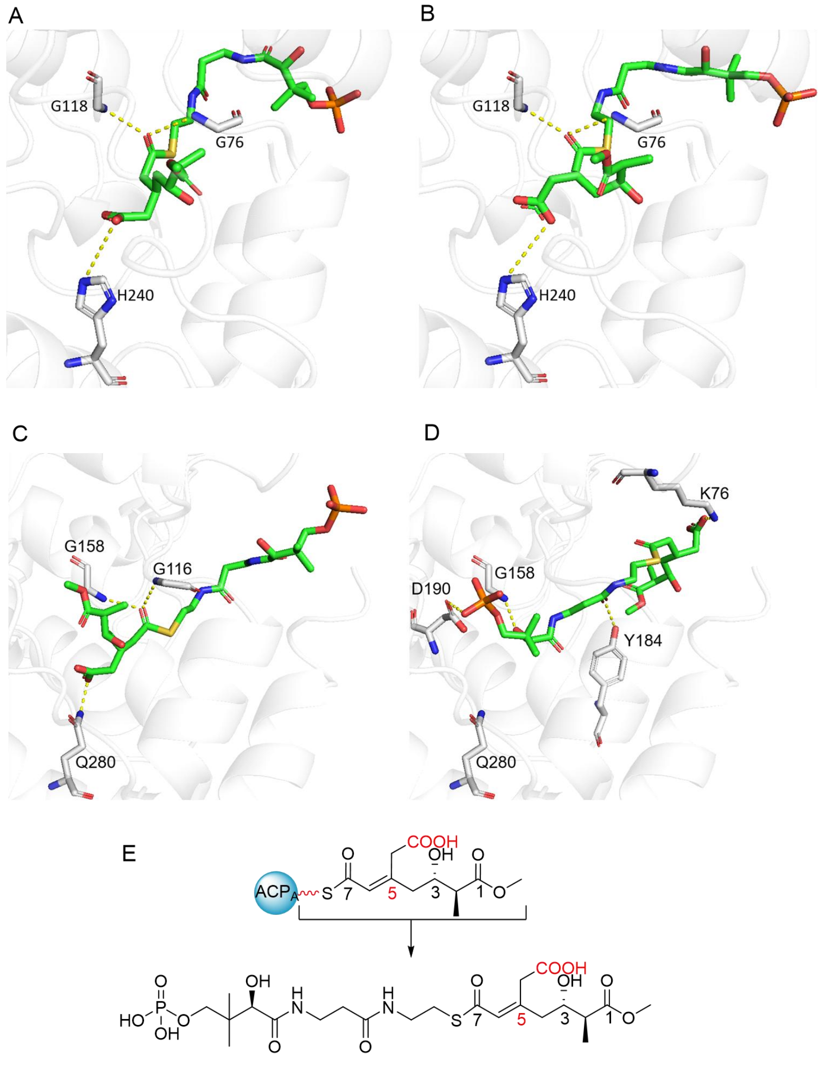

Figure S19. Comparison of the active sites of Art20 and Art21-ECH$_Q$ domain. The predicted binding modes of Art20 ((**A**) constraint, (**B**) unconstraint) and Art21-ECH$_Q$ ((**C**) constraint, (**D**) unconstraint) with a mimic substrate of Art20 (**17**) when β-branching happens on C-5 (**E**). In constraint manner, distance between H^{240} of Art20 or Q^{280} of Art21-ECH to the carboxyl terminal of **17** was limited no more than 5.5 Å. ACP_3 attaching on **17** were replaced by phosphophantetheine for docking. Both constraint (scored as -10.044) and unconstraint (scored as -8.812) docking analysis of Art20 revealed that the two conserved glycine residues (G^{76} and G^{118}) forming oxyanion hole to bind **17**, and the carboxyl terminal of **17** is adjacent to the catalytic H^{240}, the catalytic residue initiating decarboxylation. When Art21-ECH was analyzed in a constraint docking manner, with the distance between Q^{280} and the carboxyl terminal being limited to less than 5.5 Å, the configuration was scored as -7.698. When **17** was docked into Art21-ECH in an unconstraint manner (scored as -8.695), it was flipped with the phosphate terminal instead of the carboxyl terminal setting in the pocket, supporting that Art21-ECH is not an ECH_2 with decarboxylation activity.

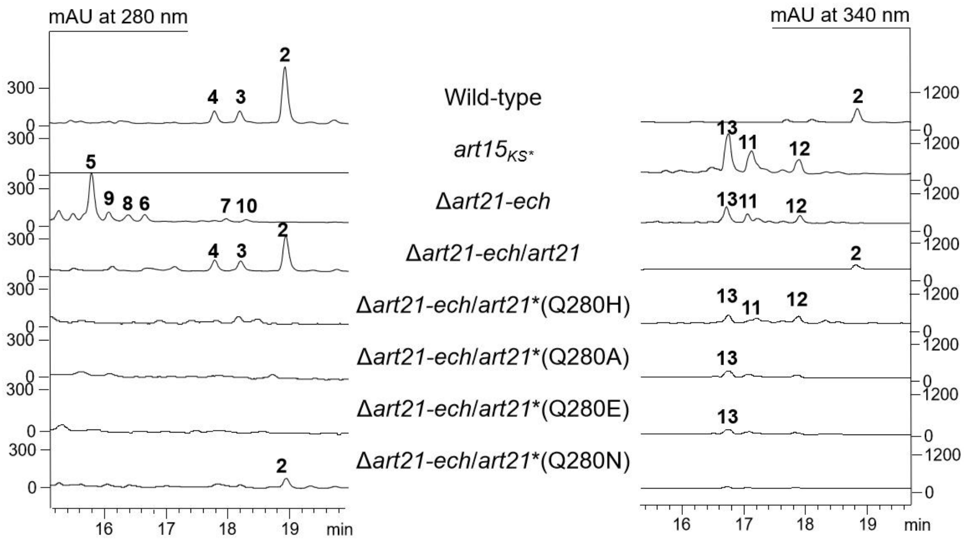

Figure S20. HPLC profiles of the *B. subtilis* wild-type and different mutant strains under both 280 nm and 340 nm.

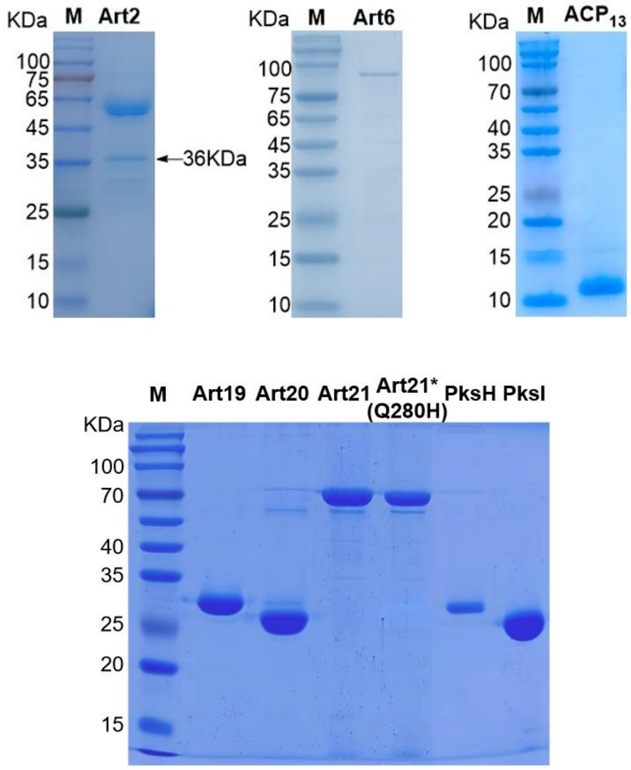

Figure S21. SDS-PAGE analyses of Art2, Art6, ACP_{13}, Art19, Art20, Art21, Art21*(Q280H), PksH and PksI. The band of Art2 was indicated with an arrow. The protein band on its top was previously identified as co-eluted chaperonin, which can help the correct folding of the target protein in *E. coli* Arctic Express (DE3).[6]

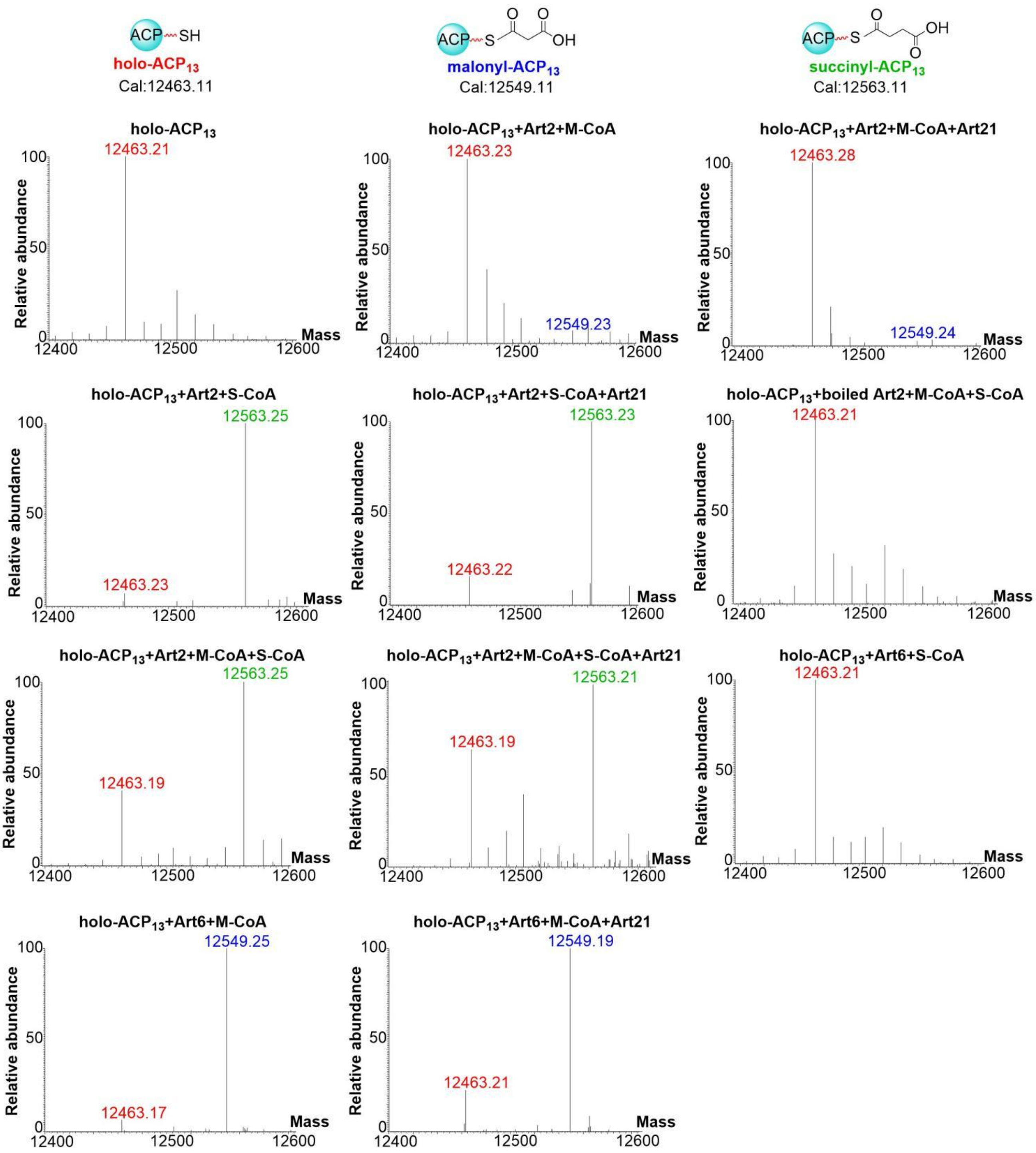

Figure S 22. Intact protein mass spectra data of the enzymatic assays that load different acyl-CoA to holo-ACP$_{13}$ by Art2 and Art6 with or without the presence of Art21.

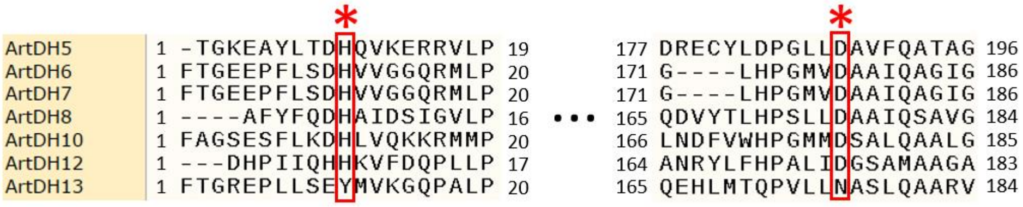

Figure S23. Multiple sequence alignment of Art DH domains. The conserved catalytic residues are indicated with red asterisks. In ArtDH$_{13}$, the histidine-aspartate dyad of typical DH active site is substituted by Tyr^{1068} and Asn^{1223}, respectively.

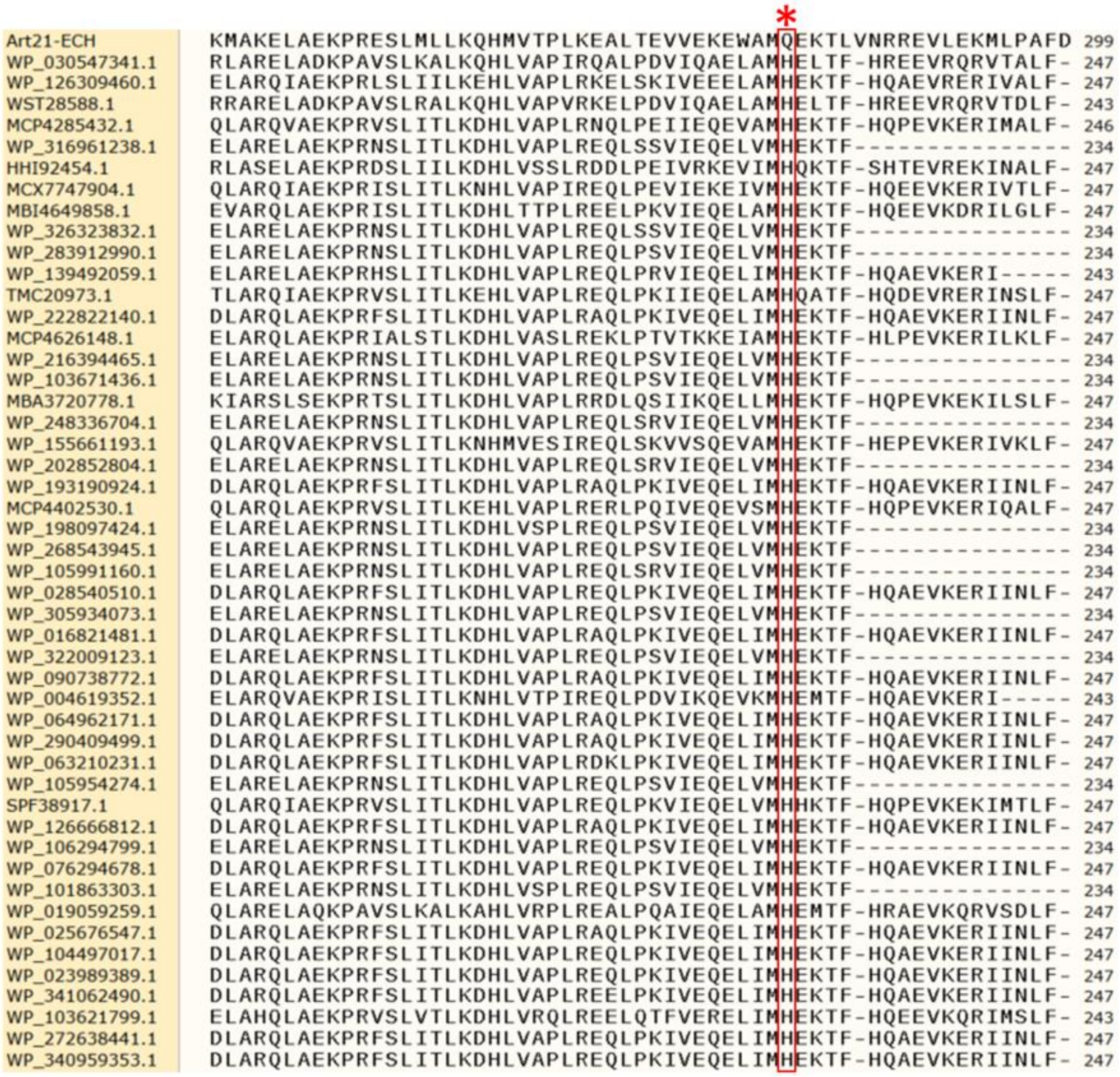

Figure S24. Multiple sequence alignment of the Art21-ECH with single domain ECH proteins. All of the single domain ECH proteins that share high similarities with Art21-ECH possess the catalytic residue H conserved as typical ECH_2.

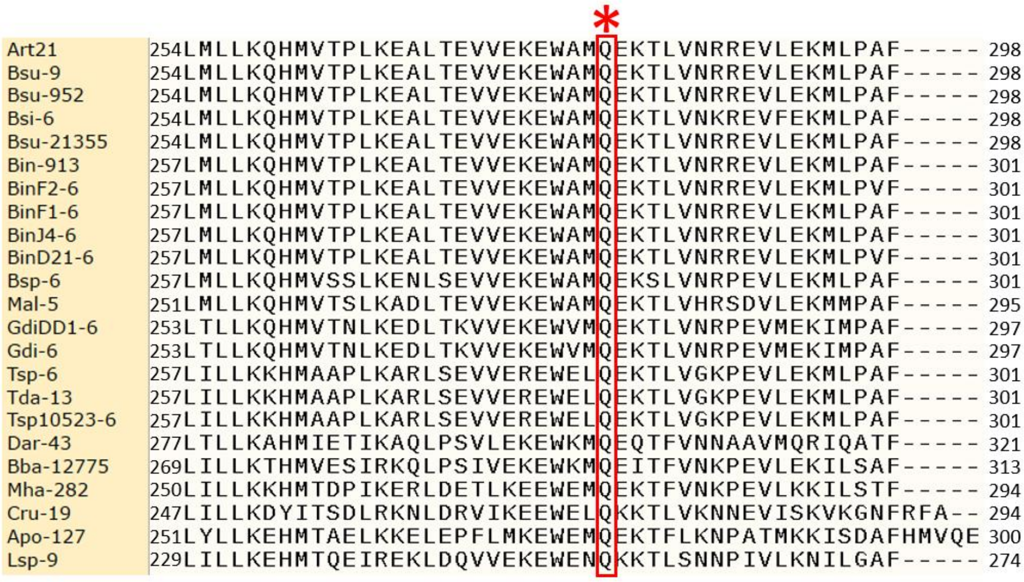

Figure S25. Multiple sequence alignment of the ECH$_Q$-TE proteins. The characteristic Gln residue (Q) was indicated with red asterisk.

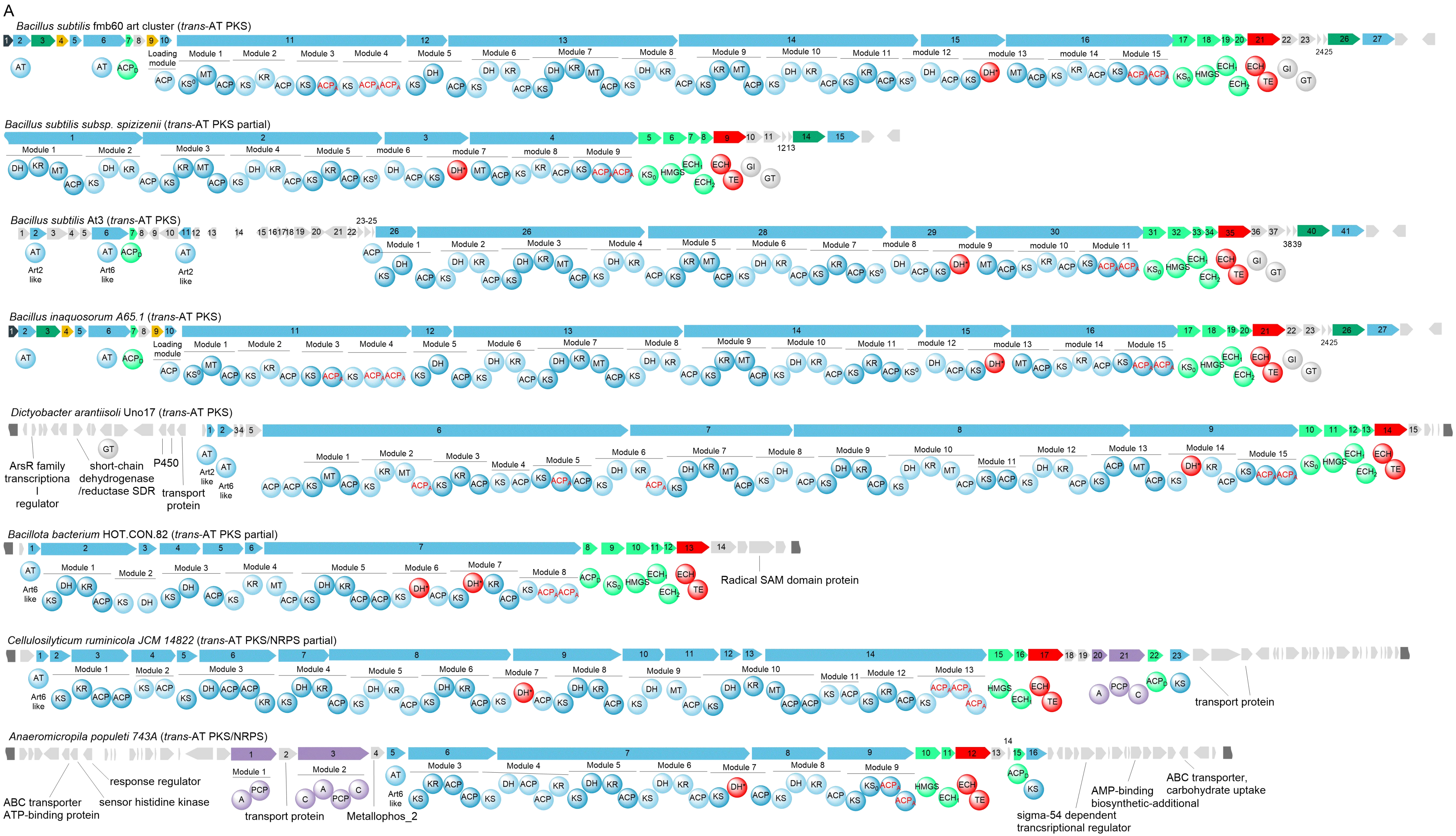

Figure S26. The biosynthetic gene clusters that contain an ECH_Q-TE encoding gene. (**A**) Schematic representatives of the biosynthetic gene clusters containing a gene encoding the ECH_Q-TE protein and genes encoding DH* domains.

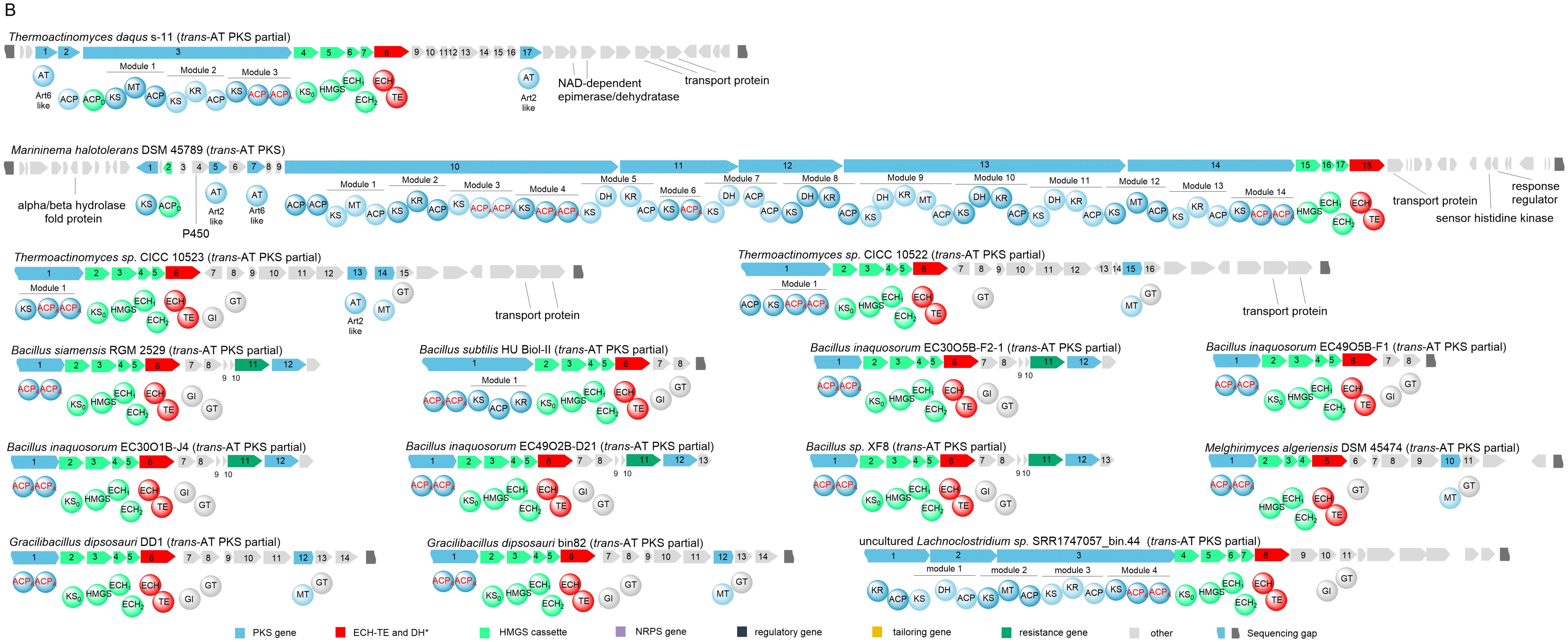

Figure S26. The biosynthetic gene clusters that contain an ECH_Q-TE encoding gene. (**B**) The other incomplete biosynthetic gene clusters that only contain a gene encoding the ECH_Q-TE protein.

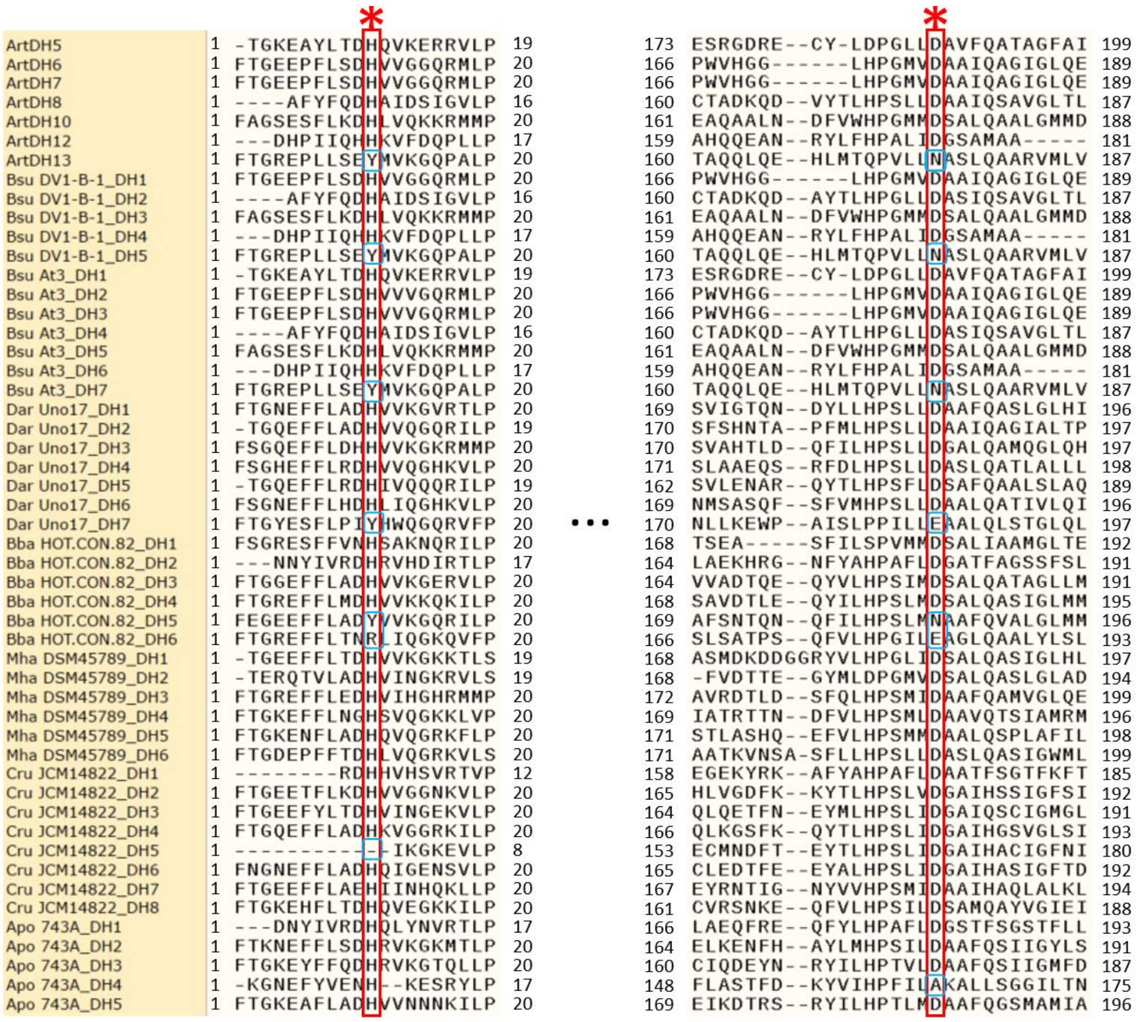

Figure S27. Multiple sequence alignment of the DH domains from the 23 biosynthetic gene clusters with an ECH_Q-TE encoding gene. The conserved His-Asp dyad of DH was indicated with red boxes and the substitution of these two residues were highlighted with blue boxes.

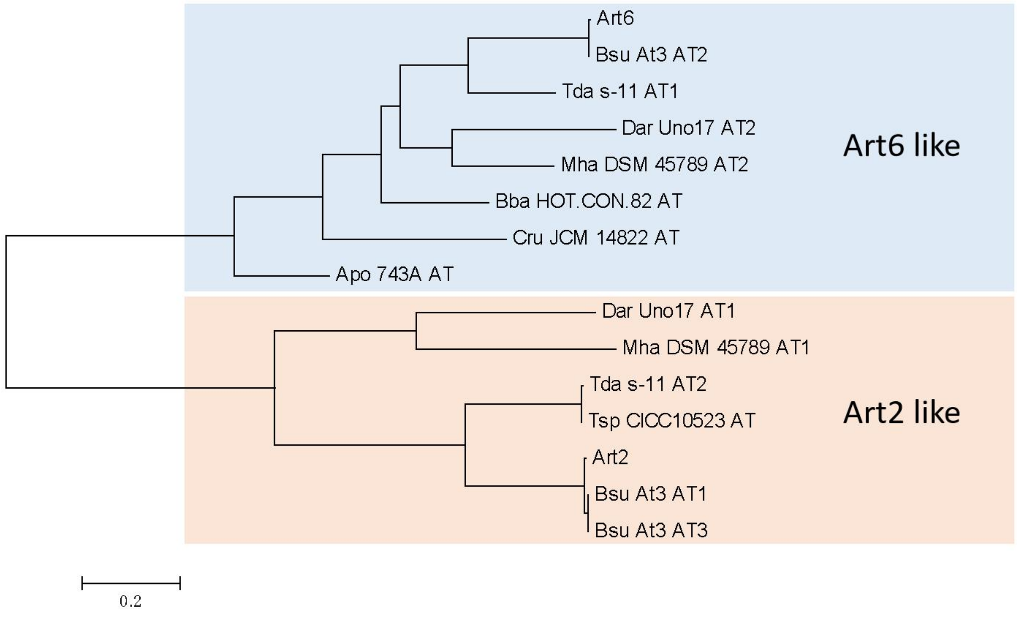

Figure S28. Phylogenetic analysis of the acyltransferases from the 23 biosynthetic gene clusters with an ECH_Q-TE encoding gene. The seven genes encoding Art2-like proteins are from *Dictyobacter arantiisoli* Uno17 (Dar Uno17 AT1), *Marininema halotolerans* DSM 45789 (Mha DSM 45789 AT1), *Thermoactinomyces daqus* s-11 (Tda s-11 AT2), *Thermoactinomyces* sp. CICC 10523 (Tsp CICC 10523 AT), *Bacillus subtilis* fmb60 (Art2), and *Bacillus subtilis* At3 (Bsu At3 AT1 and Bsu At3 AT3).

References

[1] J. Sambrook, D. Russell, Molecular Cloning: A Laboratory Manual, Third Edition; *Cold Spring Harbor Laboratory Press*: Cold Spring Harbor, New York **2001**.

[2] Y. Matsuno, T. Ano, M. Shoda, *J. Biosci. Bioeng.* **1992**, *73*, 261.

[3] A. Haines, X. Dong, Z. Song, *Nat. Chem. Biol*. **2013**, *9*, 685.

[4] T. J. Buchholz, C. M. Rath, N. B. Lopanik, N. P. Gardner, K. Håkansson, D. H. Sherman, *Chem. Biol*. **2010**, *17*, 1092.

[5] P. D. Walker, A. N. M. Weir, C. L. Willis, M. P. Crump, *Nat. Prod. Rep*. **2021**, *38*, 723.

[6] L. Liu, W. Wang, M. Chen, *Appl. Microbiol. Biotechnol.* **2023**, *107*, 2403.
